# Supplementary material for: Investigation of REFINED CNN ensemble learning for anti-cancer drug sensitivity prediction
Source: Bioinformatics. 2021 Jul 12;37(Suppl 1):i42–50. doi: 10.1093/bioinformatics/btab336 (PMC8275339; doi:10.1093/bioinformatics/btab336)
Supplement: btab336_Supplementary_Data [file btab336_supplementary_data.zip › btab336-suppl_data/Pal.29.sup.pdf]

Supplementary information to

Investigation of REFINED CNN ensemble learning for anti-cancer drug sensitivity prediction

Bazgir et al.

Correspondence: ranadip.pal@ttu.edu

1 Theoretical basis

1.1 Appendix: A.1

Recall, we have defined the predictor matrix $\boldsymbol{X} = \{x_{ij}\}, i = 1, 2, ..., n;\ j = 1, 2, ..., p$ with x_{ij} being the value of the jth feature for the ith sample. Let the number of features be p, number of candidate initial projection methods be A and let $d_{jk,a}$ be observed distance between feature j & feature k under ath projection scheme, with $\boldsymbol{d}_a = [d_{jk,a}],\ j, k = 1, 2, ..., p$ be the total number of $m = \binom{p}{2}$ distances obtained under that scheme and $\boldsymbol{d} = [\boldsymbol{d}_1, \boldsymbol{d}_2, ..., \boldsymbol{d}_A]$ be the total number of distances in the dataset. Our goal is to justify the weighted arithmetic mean specification of iREFINED.

We begin with the data model given by $d_{jk,a} \sim N(\delta_{jk}), \sigma_a^2)I(d_{jk,a} > 0)$, where
$\delta_{jk} = \sqrt{\sum_{l=1}^{2}(s_{j,l} - s_{k,l})^2}$ is distance between the feature pair (j, k) in target two dimension with $\boldsymbol{s}_j = \{s_{j1}, s_{j2}\}$ being the coordinate of jth feature in 2D and $\boldsymbol{s} = \{s_1, s_2, \cdots, s_p\}$ be the location of p features in 2D. The location process is assumed to be HPP, i.e $\boldsymbol{s} \overset{iid}{\sim} \mathcal{U}([0, 1]^2)$.

Then for a particular a, the likelihood function of the unknown parameters $\boldsymbol{s}$ and the corresponding scale parameter, σ_a^2, is given by

$$l(\boldsymbol{s}, \sigma_a^2) \propto (\sigma_a^2)^{-m/2} e^{-1/2\sum_{j>k}(\frac{d_{jk,a}-\delta_{jk}}{\sigma_a})^2} - \sum \log \Phi(\frac{\delta_{jk}}{\sigma_a}) \quad (1)$$

Under conditional independence, the full likelihood of $\boldsymbol{s}$ and $\boldsymbol{\sigma^2} = \{\sigma_1^2, \sigma_2^2, \cdots, \sigma_A^2\}$ turns out to be

$$l(\boldsymbol{s}, \boldsymbol{\sigma^2}) \propto (\Pi_a \sigma_a^2)^{-\frac{m}{2}} e^{\frac{-1}{2}\sum_{j>k}(\sum_a(\frac{d_{jk,a}-\delta_{jk}}{\sigma_a})^2)} e^{-\sum_a \sum_{j>k} \log \Phi(\frac{\delta_{jk}}{\sigma_a})} \quad (2)$$

Assuming $\sigma_a^2 \sim IG(\alpha, \beta)$ independently for $a = 1, 2, \cdots, A$, the joint prior for $\boldsymbol{\sigma}^2$ is given by
$\pi(\boldsymbol{\sigma}^2) \propto \prod_{a=1}^{A}\left[(\sigma_a^2)^{-\alpha-1}\ e^{-\frac{\beta}{\sigma_a^2}}\right]$.

The joint posterior of $[\mathbf{s}, \sigma^{\mathbf{2}}]$ turns out to be

$$\pi(\mathbf{s}, \sigma^{\mathbf{2}}|\mathbf{d}) \propto (\mathbf{\Pi_{a=1}^{A}}\sigma_{\mathbf{a}}^{\mathbf{2}})^{-(\frac{\mathbf{m}}{\mathbf{2}}+\alpha+\mathbf{1})} \mathbf{e}^{\frac{-\mathbf{1}}{\mathbf{2}}\sum_{\mathbf{j>k}}(\sum_{\mathbf{a=1}}^{\mathbf{A}}(\frac{\mathbf{d_{jk,a}}-\delta_{\mathbf{jk}}}{\sigma_{\mathbf{a}}})^{\mathbf{2}})} \\ e^{-\sum_{a=1}^{A}\sum_{j>k}\log\Phi(\frac{\delta_{jk}}{\sigma_a})} e^{-\beta\sum_{a=1}^{A}\frac{1}{\sigma_a^2}} \quad (3)$$

To obtain the full conditional of $\boldsymbol{s}$, we expand the quantity $\sum_a(\frac{d_{jk,a}-\delta_{jk}}{\sigma_a})^2$, and defining $V = \sum_{a=1}^{A}\frac{1}{\sigma_a^2}$ and $\bar{d}_{jk0} = \sum_{a=1}^{A}\frac{d_{jka}}{\sigma_a^2}$, we arrive at

$$\sum_a(\frac{d_{jk,a} - \delta_{jk}}{\sigma_a})^2 = \delta_{jk}^2 \times V - 2\delta_{jk}\bar{d}_{jk0} + \sum_a \frac{d_{jk,a}^2}{\sigma_a^2} \quad (4)$$

After the usual completion of squares of equation (**??**), and removing all the terms that do not depend upon $\boldsymbol{s}$, its full conditional is given by

$$\pi(\boldsymbol{s}|D, \underline{\sigma}^2) \propto e^{\frac{-1}{2}\sum_{j>k}V(\delta_{jk}-\frac{\bar{d}_{jk0}}{V})^2}.e^{-\sum_a\sum_{j>k}\log\Phi(\frac{\delta_{jk}}{\sigma_a})} \quad (5)$$

Observe that the full conditional of the location process is centered at $\frac{\bar{d}_{jk0}}{V} = \frac{\sum_a w_a d_{jk,a}}{\sum_a w_a}$, where $w_a = 1/\sigma_a^2$ is a function of the precision associated with the distribution of the observed distances under the projection scheme a. Consequently, the location parameter associated with the conditional posterior distribution of the location process $\boldsymbol{s}$ is a function of the weighted arithmetic mean of all the distances under consideration.

1.2 Appendix A.2

To justify the geometric mean specification in iREFINEd, we specify a log-Normal data model for $d_{jk,a}$, i.e.:
$\log(d_{jka}) \sim N(\log(\delta_{jk}), \sigma_a^2)$
Then, for the ath projection scheme, the likelihood function of $[\boldsymbol{s}, \sigma_a^2]$ is given by

$$l(\boldsymbol{s}, \sigma_a^2) \propto (\sigma_a^2)^{-\frac{m}{2}} \Pi_{j>k} \frac{1}{d_{jk,a}} e^{-\frac{1}{2}\sum_{j>k}(\frac{\log d_{jk,a} - \log \delta_{jk}}{\sigma_a})^2} \tag{6}$$

Assuming independent $IG(\alpha, \beta)$ prior on $\boldsymbol{\sigma^2}$, we get the full conditional of $[\boldsymbol{s}, \boldsymbol{\sigma}^2]$ as

$$\pi(\boldsymbol{s}, \boldsymbol{\sigma}^2 | \boldsymbol{d}) \propto (\Pi_{a=1}^A \sigma_a^2)^{-(\frac{m}{2}+\alpha+1)} e^{-\frac{1}{2}\sum_{j>k}\sum_a (\frac{d^*_{jk,a} - \delta^*_{jk}}{\sigma_a})^2} . e^{-\sum_a \frac{\beta}{\sigma_a^2}}, \tag{7}$$

where $d^*_{jk,a} = \log d_{jk,a}$ and $\delta^*_{jk} = \log \delta_{jk}$
Full conditional of $\boldsymbol{s}$ becomes

$$\pi(\boldsymbol{s}|D, \boldsymbol{\sigma}^2) \propto e^{-\frac{1}{2}\sum_{j>k}\left(\sum_{a=1}^A (\frac{d^*_{jk,a} - \delta^*_{jk}}{\sigma_a})^2\right)} \tag{8}$$

Now consider

$$\sum_{a=1}^A (\frac{d^*_{jk,a} - \delta^*_{jk}}{\sigma_a})^2 = \sum_a \frac{(d^*_{jk,a})^2}{\sigma_a^2} - 2\delta^*_{jk} \sum \frac{d^*_{jk,a}}{\sigma_a^2} + \sum_a \frac{(\delta^{*2}_{jk,a})}{\sigma_a^2} \tag{9}$$

Define $V = \sum \frac{1}{\sigma_a^2}$ and $\bar{d}^*_{jk0} = \sum_a \frac{d^*_{jk,a}}{\sigma_a^2}$ and complete the square with respect to δ^*_{jk}. Then removing all the terms that are not functions of δ^*, we get

$$\pi(\boldsymbol{s}|\boldsymbol{d}, \boldsymbol{\sigma}^2) \propto e^{-\frac{1}{2}\sigma_{j>k} V (\delta^*_{jk} - \frac{\bar{d}^*_{jk0}}{V})^2} \tag{10}$$

Now:

$$\begin{aligned} \frac{\bar{d}^*_{jk0}}{V} &= \frac{\sum \frac{1}{\sigma^a} \log d_{jk,a}}{\sum \frac{1}{\sigma_a^2}} \\ &= \frac{\sum w_a \log d_{jk,a}}{\sum w_a} \\ &= \log \tilde{d}_{jk} \end{aligned} \tag{11}$$

Where $\tilde{d}_{jk}$ is the weighted geometric mean of $(d_{jk,1}, ..., d_{jk,A})$ with the weight being a function of the precision associated with data model for projection schemes. So,

$$\pi(\boldsymbol{s}|\boldsymbol{d}, \boldsymbol{\sigma}^2) \propto e^{-\frac{1}{2}\sum_{j>k} V(\log \delta_{jk} - \log \tilde{d}_{jk})^2} \tag{12}$$

Clearly in log-scale, the distribution of δ_{jk} is centered around the weighted geometric mean of observed distances across different projection schemes.

2 Comparison with the state-of-the-art and baseline models

In this section we briefly explain the state-of-the-art and baseline models, and discuss their similarities/dissimilarities in table , that we used for comparison with REFINED-CNN in this study.

KBMTL: Kernelized Bayesian Multitask Learning (KBMTL) is a novel Bayesian-learning-based technique that integrates kernel-based non-linear dimensionality reduction (manifold learning) and classification or regression to predict drug susceptibility against panel of drugs. Therefore, the state-of-the-art KBMTL was considered as a competitive model to REFINED CNNs for drug sensitivity prediction.

Xie et al 2018: Offered a deep learning-based model for predicting cell line response to a subset of drug pairs in the NCI-ALMANAC database. They combine molecular feature types of the cell lines (gene expression, microRNA, and proteome) with the SMILE chemical descriptors of the drugs to predict the ComboScore. Hence, Xie et al network was utilized for comparison, except that we use PaDEL chemical descriptors instead of SMILE to create REFINED image for each drug. Also, we do not combine omics data with the chemical drug descriptors to avoid substitution error.

DeepSynergy: was developed for identification and prediction of drug responses to the synergistic combination of drugs in cancer treatment. DeepSynergy is a deep learning-based model that is capable of deploying heterogeneous data—genomics and chemical information. Preuer et al used *OpenBabel* to calculate the chemical features of the drugs with additional 3 stages post processing to include 1) 1309 $ECFP\_^6$ 2) 802 physiochemical, 3) 2276 toxicophoric features. They utilized the gene expression profile of the cell lines as another input of the DeepSynergy model. We used the DeepSynergy as a competitive state-of-the-art model to compare its prediction performance with REFINED CNNs on drug combination *"ComboScore"* of the NCI-ALMANAC dataset.

XGBoost: Extreme Gradient Boosting—known as XGBoost—is a supervised learning algorithm based on boosting regularized tree models, that performs aka embedded ensemble learning in the training phase. Hence, we compared the XGBoost, as a baseline, with our REFINED-CNN and their ensemble version.

RF: Random decision forest (RF) is an ensemble supervised learning-based method that construct bootstrap aggregation of multiple decision trees on a training set, and offers the mean of the trees as the ultimate prediction. Because of the generalizable characteristics of the RF, we used it as baseline for comparison of our proposed models.

SVR: Support Vector Regression (SVR) is a supervised learning regression model that aims to fit appropriate hyperplanes in high dimensional space, given the defined acceptable error range (tolerant) and the kernel, on covariates for prediction. Based on the concept of REFINED–a mapping in high dimensional space into 2D space–we used SVR as an alternate baseline model for comparison.

EN: Elastic Net (EN) is a regularized linear regression model that integrates L1 and L2 norm penalties of the lasso and ridge regression. We used EN as a linear model for comparison to elaborate on the complexity of the problem and the data.

Table 1 summarizes characteristics of each competing model.

Table 1: State-of-the-art and baseline models used for comparison with the REFINED-CNN models in summary.

Mdoel	Description	Goal
REFINED-CNN model stacking	Deep Learning, Manifold Learning, Ensemble Learning	General (applied on drug sensitivity prediction here)
REFINED-CNN image stacking	Deep Learning, Manifold Learning, Ensemble Learning	General (applied on drug sensitivity prediction here)
iREFINED-CNN	Deep Learning, Manifold Learning, Ensemble Learning	General (applied on drug sensitivity prediction here)
sREFINED-CNN	Deep Learning, Manifold Learning	General (applied on drug sensitivity prediction here)
Deep Synergy	Deep Learning	General (applied on drug sensitivity prediction here)
Xie et al.	Deep Learning	Drug sensitivity prediction
KBMTL	Statistical Learning, Kernel-based, Bayesian, Manifold Learning	Drug sensitivity prediction
XGBoost	Statistical Learning, Decision Tree, Ensemble Learning	Drug sensitivity prediction
RF	Statistical Learning, Decision Tree, Ensemble Learning	General
SVR	Statistical Learning, Kernel based	General
EN	Statistical Learning, Linear	General

3 CNN hyperparameters

In this section we provide the hyperparameters that we used to train each REFINED-CNN model specifically. The table 2 and 3 specify the hyperparameters for the REFINED-CNN models trained on NCI60 and NCI-ALMANAC datasets, respectively.

Table 2: Hyperparameters of the all REFINED CNN models optimized with the Bayesian optimization process for the NCI60 dataset. Model 1: sREFINED with MDS, Model 2: sREFINED with LLE, Model 3: sREFINED with LE, Model 4: sREFINED with Isomap, Model 5: REFINED-CNN model stacking, Model 6: REFINED-CNN image stacking, Model 7: i-REFINED-CNN-GM, Model 8: iREFINED-CNN-AM.

Hyperparameter	Model 1	Model 2	Model 3	Model 4	Model 5	Model 6	Model 7	Model 8
Learning rate	0.0001	0.0001	0.0001	0.0001	0.0001	0.00071	0.00093	0.00099
Conv1 kernel size	7	7	7	7	7	5	5	5
Conv2 kernel size	7	7	7	7	7	5	5	5
Conv1 #kernel	64	64	64	64	64	48	35	54
Conv2 #kernel	128	128	128	128	128	128	117	49
Dense1 #neurons	256	256	256	256	256	300	540	598
Dense2 #neurons	64	64	64	64	64	40	20	43
Dropout probability	0.7	0.7	0.7	0.7	0.7	0.7	0.7	0.7

Table 3: Hyperparameters of the all REFINED CNN models optimized with the Bayesian optimization process for the NCI-ALMANAC dataset. Model 1: sREFINED with MDS, Model 2: sREFINED with LLE, Model 3: sREFINED with LE, Model 4: sREFINED with Isomap, Model 5: REFINED-CNN model stacking, Model 6: REFINED-CNN image stacking, Model 7: i-REFINED-CNN-GM, Model 8: iREFINED-CNN-AM.

Hyperparameter	Model 1	Model 2	Model 3	Model 4	Model 5	Model 6	Model 7	Model 8
Learning rate	0.0008	0.0008	0.0008	0.0008	0.0008	0.0008	0.00059	0.0008
Conv1 kernel size	5	5	5	5	5	5	5	5
Conv2 kernel size	5	5	5	5	5	5	5	5
Conv1 #kernel	41	41	41	41	41	36	50	37
Conv2 #kernel	87	87	87	87	87	218	74	128
Dense1 #neurons	100	100	100	100	100	180	100	100
Dense2 #neurons	100	100	100	100	100	60	80	100
Dropout probability	0.7	0.7	0.7	0.7	0.7	0.7	0.7	0.7

4 Feature map stacking

In this section, we design the 4 different convolution layers in parallel, where each convolution layer is associated with one of the REFINED images initialized with one of the chosen manifold learning techniques (MDS, Isomap, LE, LLE). Hence the REFINED-CNN model takes 4 different images as the input, convolve them with 4 different set of kernels separately, then the extracted features (feature maps) through the convolution process are concatenated. In other words, we design a first convolution layer to have c_1 filters of size k_1 applied on REFINED MDS, c_2 filters of size k_2 applied on REFINED Isomap, c_3 filters of size k_3 applied on REFINED LE and c_4 filters of size k_4 applied on REFINED LLE. Then, we concatenate the output feature maps of these convolutions layers. The hyper parameters are tuned with the Bayesian optimization process.

We conducted the feature map stacking in two different approaches based on the choice of padding in the convolution block. If we choose *valid* option for padding, then no padding is performed and the output size of the feature map varies based on the kernel size and stride, hence we cannot concatenate feature maps and apply another convolution layer as they have different sizes. However, if we choose the *same* option, then all the feature maps have the same size regardless of the choice of kernel size or stride, therefore the feature map can be concatenated, and another convolution layer be applied on them. This investigation takes the receptive field of the kernels into considerations while we design our REFINED-CNN based predictive model. The table 4 and 5 show the results of REFINED-CNN with feature map stacking in average along with other REFINED-CNN based models introduced in this study for NCI60 and NCI-ALMANAC datasets, respectively. As the results indicate, for both datasets, the REFINED-CNN feature map stacking is under performing other single REFINED-CNNs and ensemble REFINED-CNNs, because it introduces more parameters to the model, which makes the REFINED-CNN feature map stacking prone to over-fitting. The REFINED-CNN feature mapping is less generalizable as compared to the models, according to our optimization process. We optimize each model by searching over prior distribution of hyperparameters using training data of only one cell line. Once we find the hyperparameters using the selected cell line–HCC_2998 for both NCI60 and NCI-ALMANAC datasets– we train and test the REFINED-CNN models on the reported cell lines. As the results indicate, the hyperparameters optimized using the "HCC_2998" cell line is nearly optimal for other cell lines in all the models except for the REFINED-CNN FM stackings.

In tables tables 4 and 5 the "REFINED-CNN FM stacking 1" contains only one convolution layer for each REFINED image with *valid* padding option, then their output features are concatenated as input of a network of dense layers. The "REFINED-CNN FM stacking 2" contains one convolution layer for each REFINED image with *same* padding option in parallel with each other followed by another convolution layer with *valid* padding option prior to the network of dense layers. The "REFINED-CNN FM stacking 1" contains 4 convolution layers in parallel for modeling the NCI60 dataset, while it contains 8 convolu-

tion layers to model the NCI-ALMANAC dataset. The "REFINED-CNN FM stacking 1" has 8 convolution layers as it models response of combination of 2 drugs, and per each drug there is a set of 4 images. The "REFINED-CNN FM stacking 2" contains 9 convolution layers in total where 8 of them, same as the "REFINED-CNN FM stacking 1", carry the 8 input images of the model and then their contcatenated feature maps is the input of the 9th convolution layer.

Table 4: REFINED-CNN feature map (FM) stacking with 1 and 2 convolution layers compared with other REFINED-CNN based models for the NCI60 dataset.

Models	NRMSE	NMAE	PCC	Bias
REFINED-CNN with MDS	0.778	0.709	0.649	0.488
REFINED-CNN with Isomap	0.786	0.716	0.644	0.508
REFINED-CNN with LE	0.788	0.72	0.643	0.504
REFINED-CNN with LLE	0.795	0.759	0.625	0.511
REFINED-CNN image stacking	0.775	0.679	0.655	0.509
REFINED-CNN model stacking	0.702	0.653	0.71	0.489
iREFINED-CNN-AM	0.715	0.63	0.706	0.461
iREFINED-CNN-GM	0.722	0.635	0.705	0.446
REFINED-CNN FM stacking 1	1.295	1.112	0.528	0.579
REFINED-CNN FM stacking 2	0.797	0.736	0.651	0.499

Table 5: REFINED-CNN feature map (FM) stacking with 1 and 2 convolution layers compared with other REFINED-CNN based models for the NCI-ALMANAC dataset.

Models	NRMSE	NMAE	PCC	Bias
REFINED-CNN with MDS	0.514	0.474	0.877	0.259
REFINED-CNN with Isomap	0.508	0.470	0.887	0.227
REFINED-CNN with LE	0.489	0.443	0.884	0.238
REFINED-CNN with LLE	0.522	0.486	0.876	0.292
REFINED-CNN image stacking	0.561	0.524	0.845	0.370
REFINED-CNN model stacking	0.420	0.361	0.907	0.168
iREFINED-CNN-AM	0.480	0.431	0.893	0.275
iREFINED-CNN-GM	0.474	0.427	0.892	0.248
REFINED-CNN FM stacking 1	0.969	0.933	0.712	0.301
REFINED-CNN FM stacking 2	0.764	0.858	0.824	0.302

We provide the complete results of the REFINED-CNN feature map stacking for all the cell lines of the NCI60 and NCI-ALMANAC datasets reported in this study through table tables 6 and 7.

Table 6: REFINED-CNN FM stacking performance for each cell line of the NCI60 dataset reported in this study.

Cell lines	REFINED-CNN FM stacking 1				REFINED-CNN FM stacking 2			
	NRMSE	NMAE	PCC	Bias	NRMSE	NMAE	PCC	Bias
HCC_2998	0.897	0.971	0.618	0.579	0.790	0.862	0.665	0.443
MDA_MB_435	0.815	0.889	0.588	0.618	1.334	1.199	0.618	0.512
SNB_78	2.780	1.714	0.09	0.841	1.311	1.294	0.532	0.610
NCI_ADR_RES	0.850	0.882	0.583	0.59	0.809	0.880	0.642	0.424
DU_145	0.814	0.892	0.597	0.584	0.789	0.881	0.651	0.476
786_0	0.789	0.879	0.63	0.556	0.746	0.845	0.689	0.410
A498	0.876	0.953	0.598	0.570	0.778	0.858	0.651	0.474
A549_ATCC	0.812	0.864	0.642	0.550	0.736	0.831	0.692	0.430
ACHN	0.769	0.866	0.647	0.519	0.726	0.837	0.699	0.429
BT_549	0.826	0.891	0.571	0.63	0.889	0.956	0.630	0.485
CAKI_1	0.831	0.902	0.568	0.636	0.783	0.873	0.656	0.443
DLD_1	3.704	2.193	0.289	0.626	0.995	1.078	0.669	0.472
DMS_114	1.965	1.473	0.370	0.367	0.966	1.000	0.655	0.493
DMS_273	2.827	1.880	0.371	0.518	0.777	0.867	0.689	0.431
CCRF_CEM	0.840	0.904	0.598	0.555	0.789	0.853	0.657	0.462
COLO_205	0.781	0.859	0.635	0.542	0.756	0.846	0.693	0.384
EKVX	0.849	0.896	0.582	0.576	0.828	0.884	0.631	0.479
Average	1.296	1.112	0.528	0.580	0.871	0.932	0.654	0.462

Table 7: REFINED-CNN FM stacking performance for each cell line of the NCI-ALMANAC dataset reported in this study.

Cell lines	REFINED-CNN FM stacking 1				REFINED-CNN FM stacking 2			
	NRMSE	NMAE	PCC	Bias	NRMSE	NMAE	PCC	Bias
786-0	0.743	0.794	0.73	0.343	0.616	0.743	0.818	0.378
A498	0.795	0.915	0.784	0.301	0.606	0.738	0.802	0.302
A549/ATCC	0.660	0.781	0.809	0.32	0.657	0.775	0.846	0.352
ACHN	0.594	0.729	0.839	0.223	0.475	0.643	0.885	0.187
CCRF-CEM	0.583	0.742	0.835	0.228	0.530	0.696	0.853	0.215
COLO 205	1.796	1.192	0.409	0.24	0.738	0.867	0.839	0.228
DU-145	0.713	0.773	0.779	0.344	1.130	1.097	0.828	0.316
EKVX	0.686	0.785	0.775	0.349	0.588	0.728	0.816	0.339
HCC-2998	0.613	0.756	0.808	0.267	0.602	0.750	0.817	0.285
HCT-15	0.559	0.712	0.833	0.298	0.937	0.970	0.827	0.299
HCT-116	1.553	1.296	0.721	0.315	0.595	0.732	0.807	0.340
HL-60(TB)	0.680	0.81	0.808	0.271	0.582	0.734	0.818	0.294
HOP-62	1.127	0.985	0.555	0.328	0.635	0.782	0.846	0.311
HOP-92	0.663	0.773	0.766	0.29	0.536	0.700	0.847	0.246
HT29	0.987	0.998	0.671	0.401	1.065	1.058	0.786	0.347
IGROV1	2.041	1.487	0.574	0.337	0.972	1.012	0.802	0.329
K-562	0.832	0.885	0.744	0.283	0.935	1.017	0.802	0.307
KM12	1.510	1.24	0.549	0.443	0.873	0.948	0.774	0.413
LOX IMVI	0.665	0.773	0.833	0.14	0.648	0.807	0.860	0.255
MALME-3M	1.583	1.23	0.413	0.305	1.559	1.369	0.805	0.296
Average	0.969	0.933	0.712	0.301	0.764	0.858	0.824	0.302

5 Supplementary Results

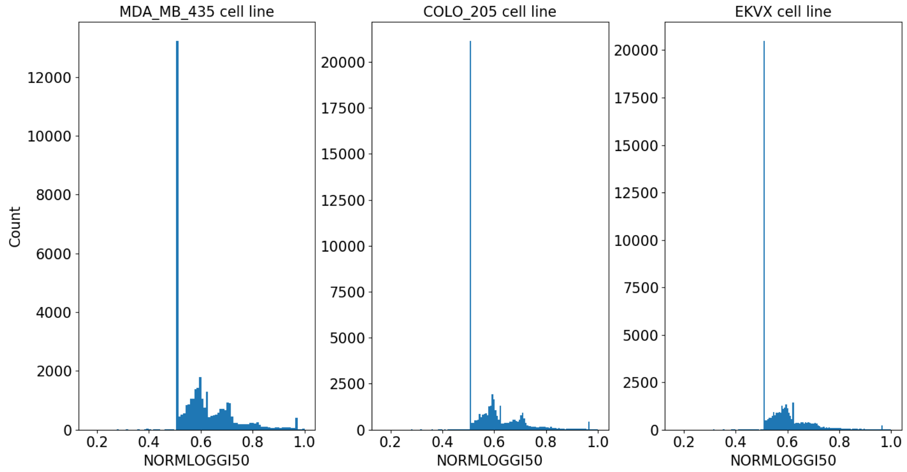

Figure 1: Normalized log of GI50 for three cell lines selected randomly from NCI60 dataset. The existing large peaks indicates the number of drugs that is not sensitive to that cell line.

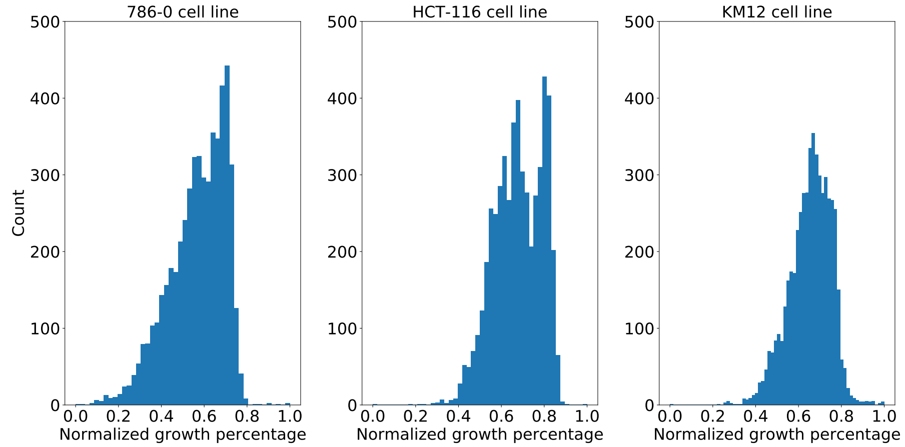

Figure 2: Normalized growth percentage of ComboScore distribution for three cell lines selected randomly from NCI-ALMANAC dataset.

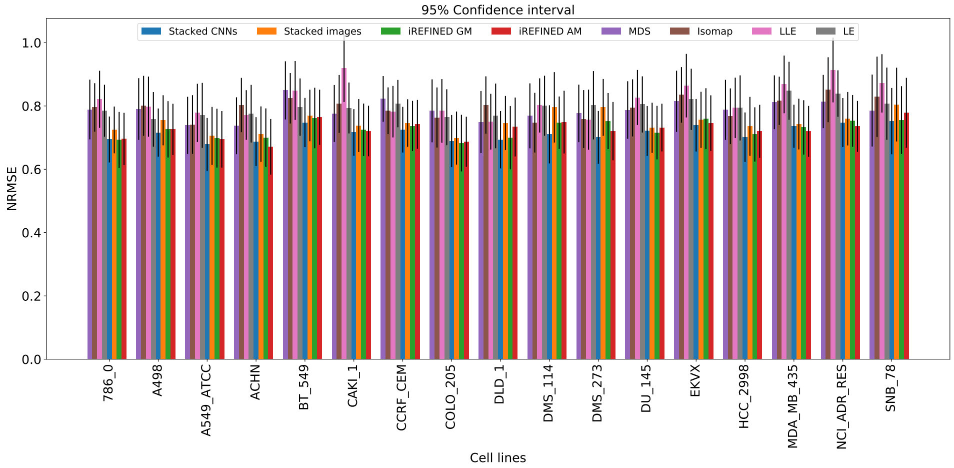

Figure 3: NRMSE with 95 % confidence interval of each model trained on 17 cell lines of NCI60 dataset, reported for each line per model separately.

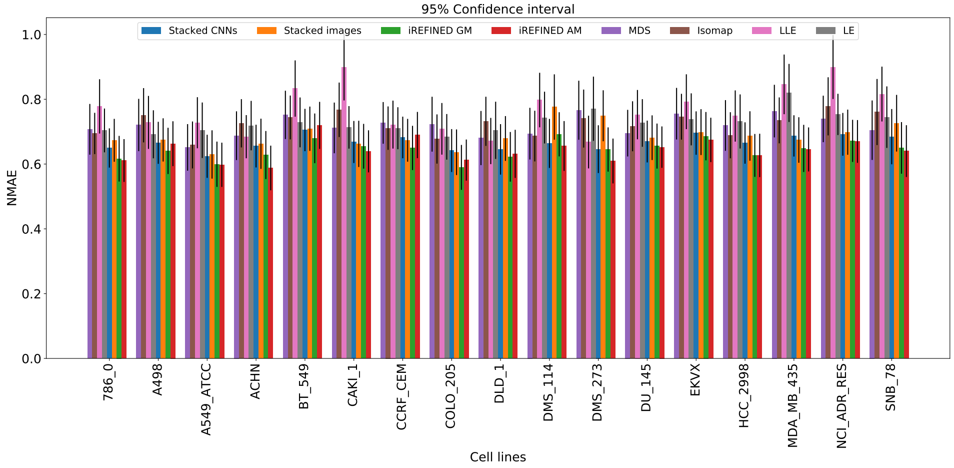

Figure 4: NMAE with 95 % confidence interval of each model trained on 17 cell lines of NCI60 dataset, reported for each line per model separately.

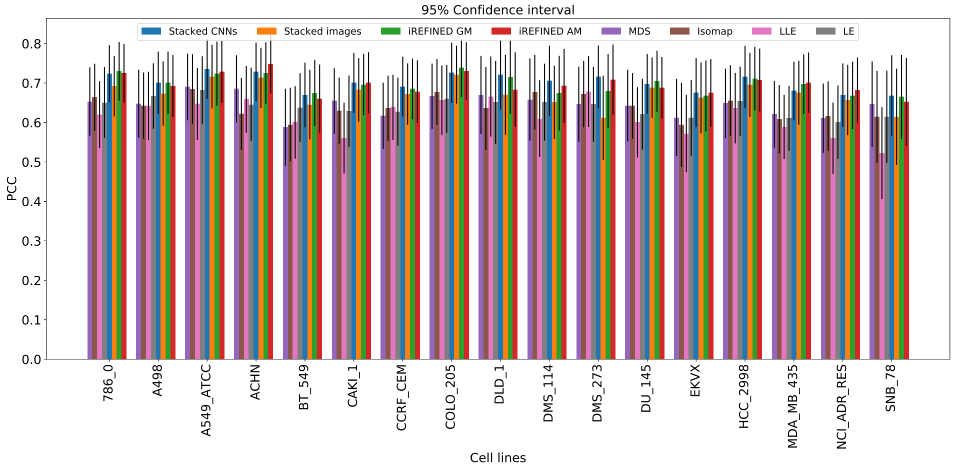

Figure 5: PCC with 95 % confidence interval of each model trained on 17 cell lines of NCI60 dataset, reported for each line per model separately.

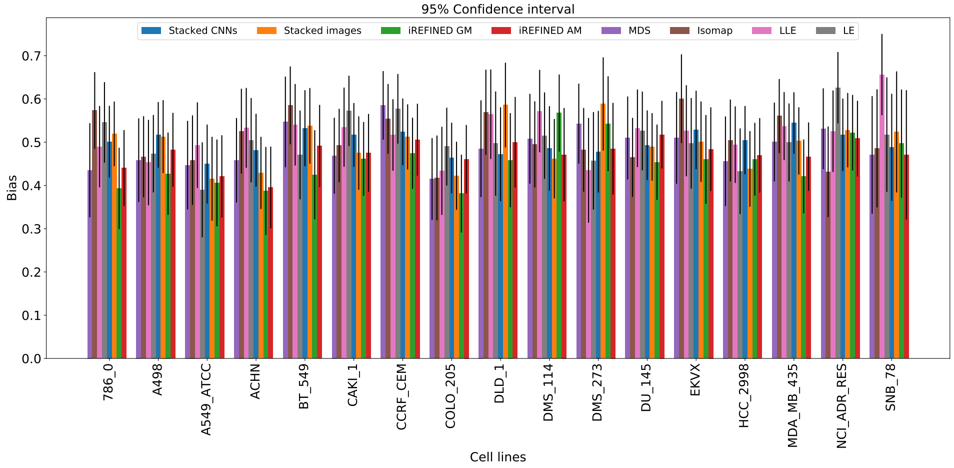

Figure 6: Bias with 95 % confidence interval of each model trained on 17 cell lines of NCI60 dataset, reported for each line per model separately.

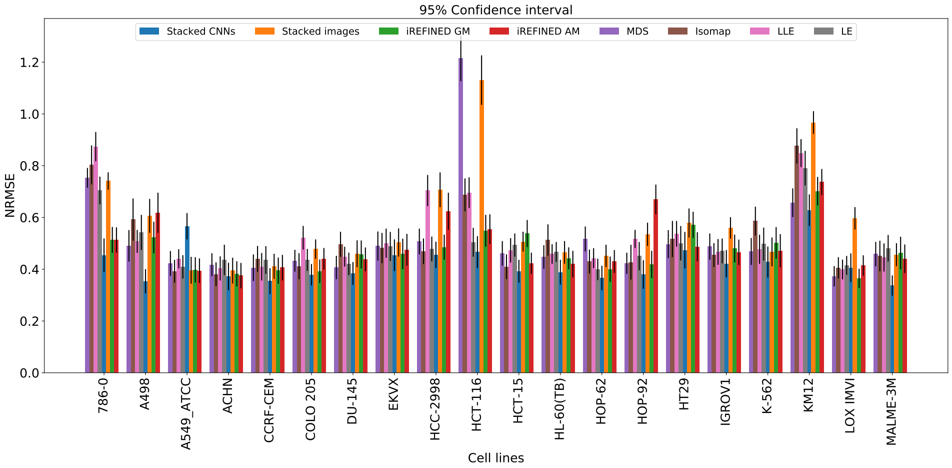

Figure 7: NRMSE with 95 % confidence interval of each model trained on 17 cell lines of NCI-ALMANAC dataset, reported for each line per model separately.

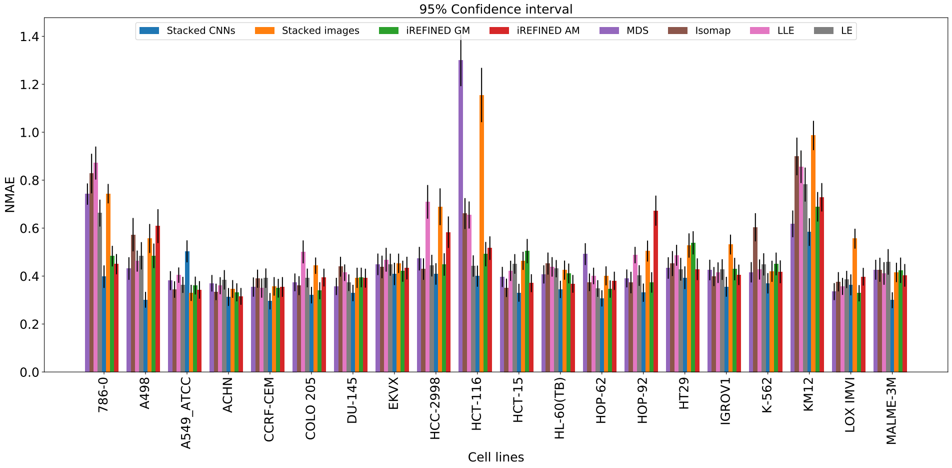

Figure 8: NMAE with 95 % confidence interval of each model trained on 17 cell lines of NCI-ALMANAC dataset, reported for each line per model separately.

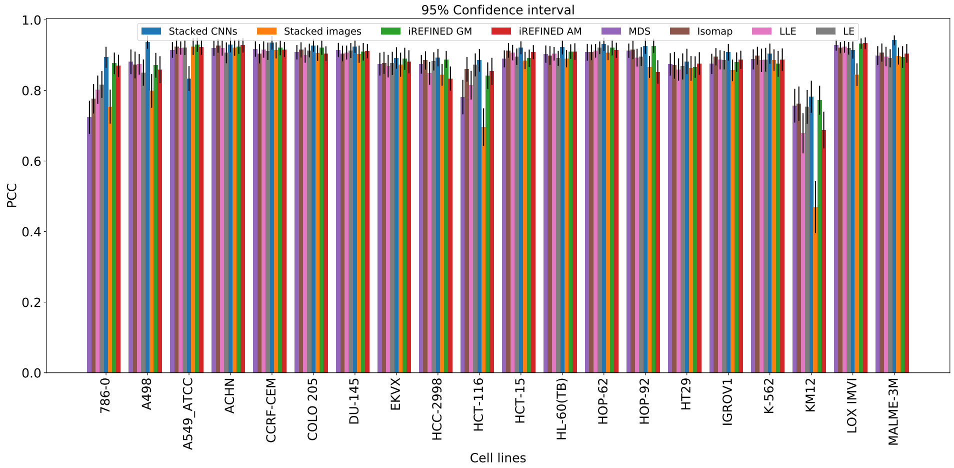

Figure 9: PCC with 95 % confidence interval of each model trained on 17 cell lines of NCI-ALMANAC dataset, reported for each line per model separately.

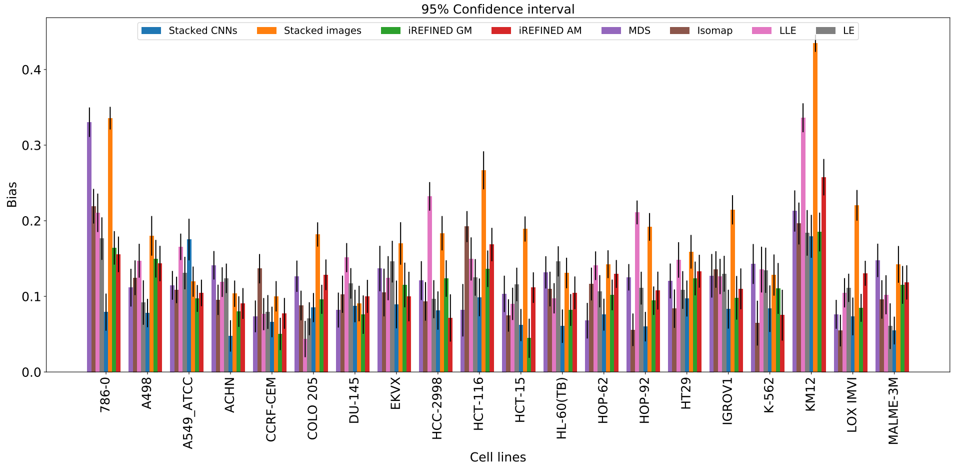

Figure 10: Bias with 95 % confidence interval of each model trained on 17 cell lines of NCI-ALMANAC dataset, reported for each line per model separately.

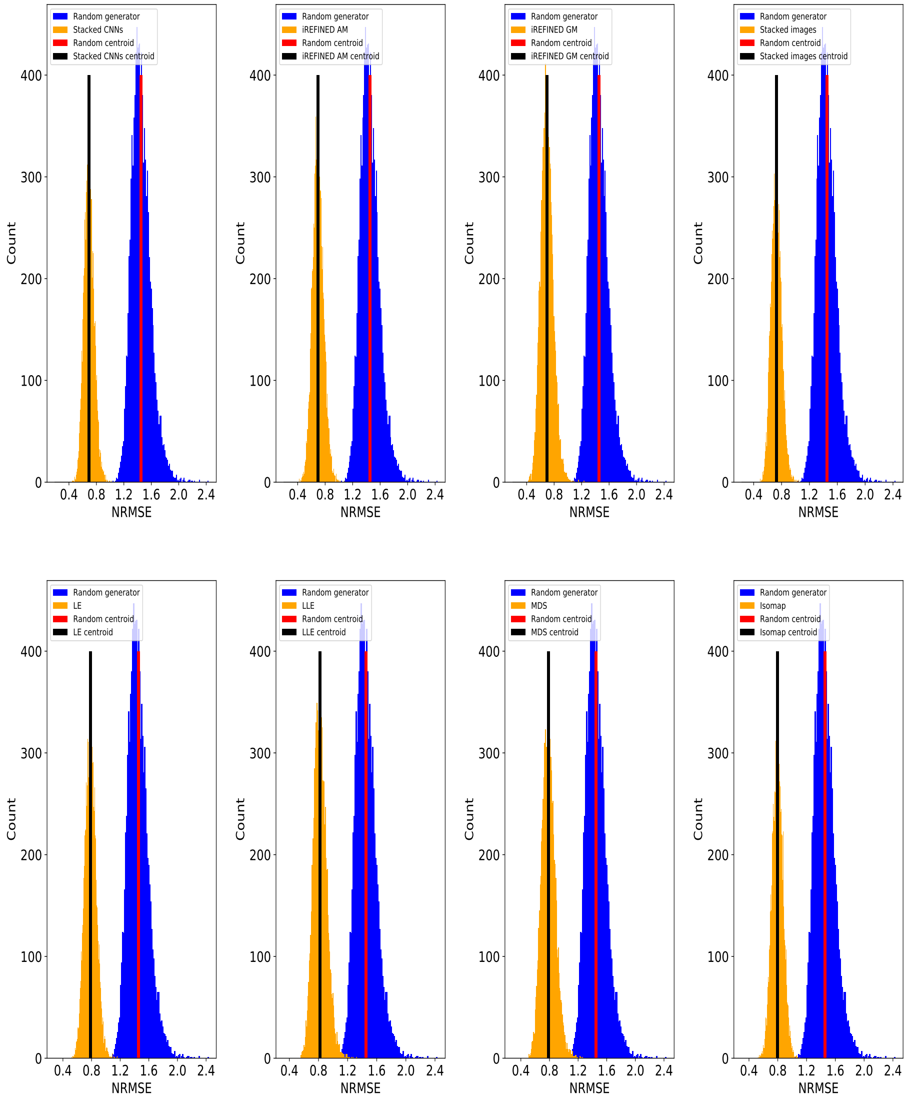

Figure 11: Distribution of NRMSE of all eight models drawn from the Gap statistics test for the 780_0 cell line of the NCI60 dataset. The distributions clustered into two groups and their associated cluster centroids are shown with a vertical bar on the histogram plots.

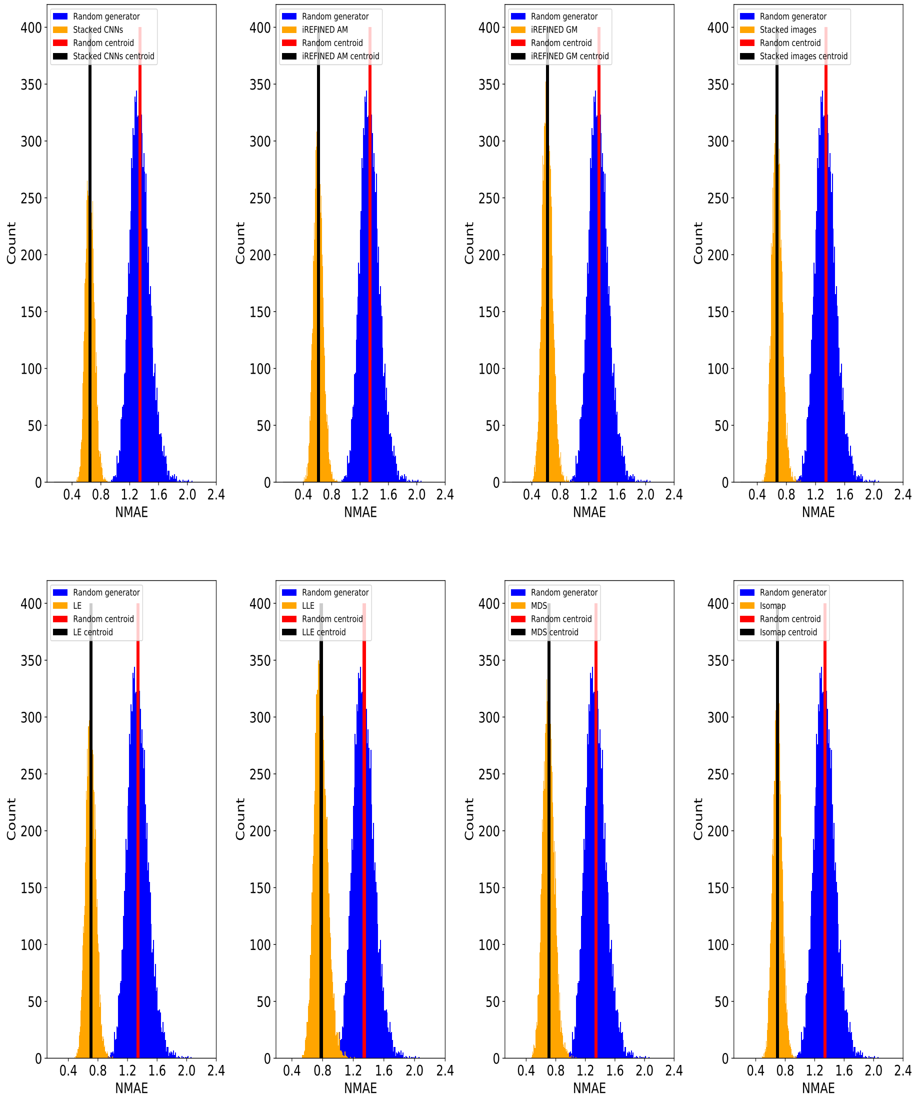

Figure 12: Distribution of NMAE of all eight models drawn from the Gap statistics test for the 780_0 cell line of the NCI60 dataset. The distributions clustered into two groups and their associated cluster centroids are shown with a vertical bar on the histogram plots.

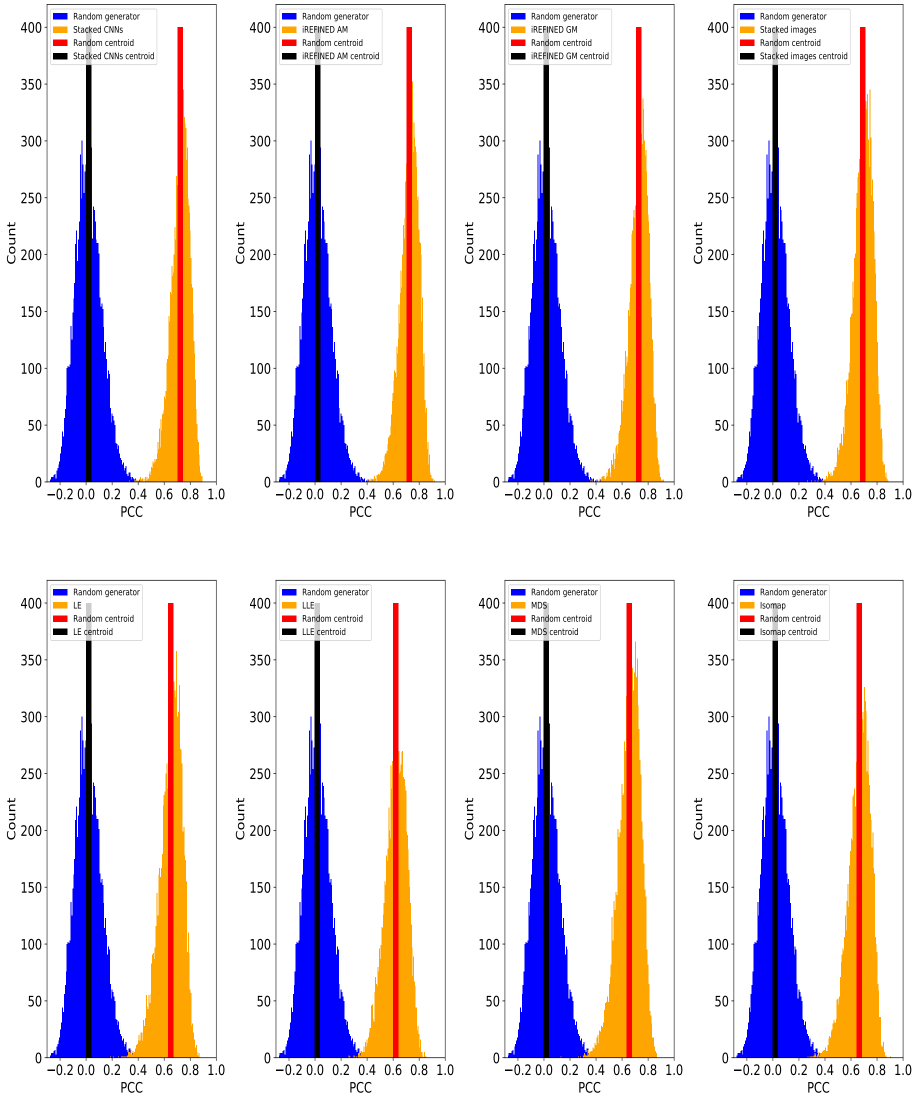

Figure 13: Distribution of PCC of all eight models drawn from the Gap statistics test for the 780_0 cell line of the NCI60 dataset. The distributions clustered into two groups and their associated cluster centroids are shown with a vertical bar on the histogram plots.

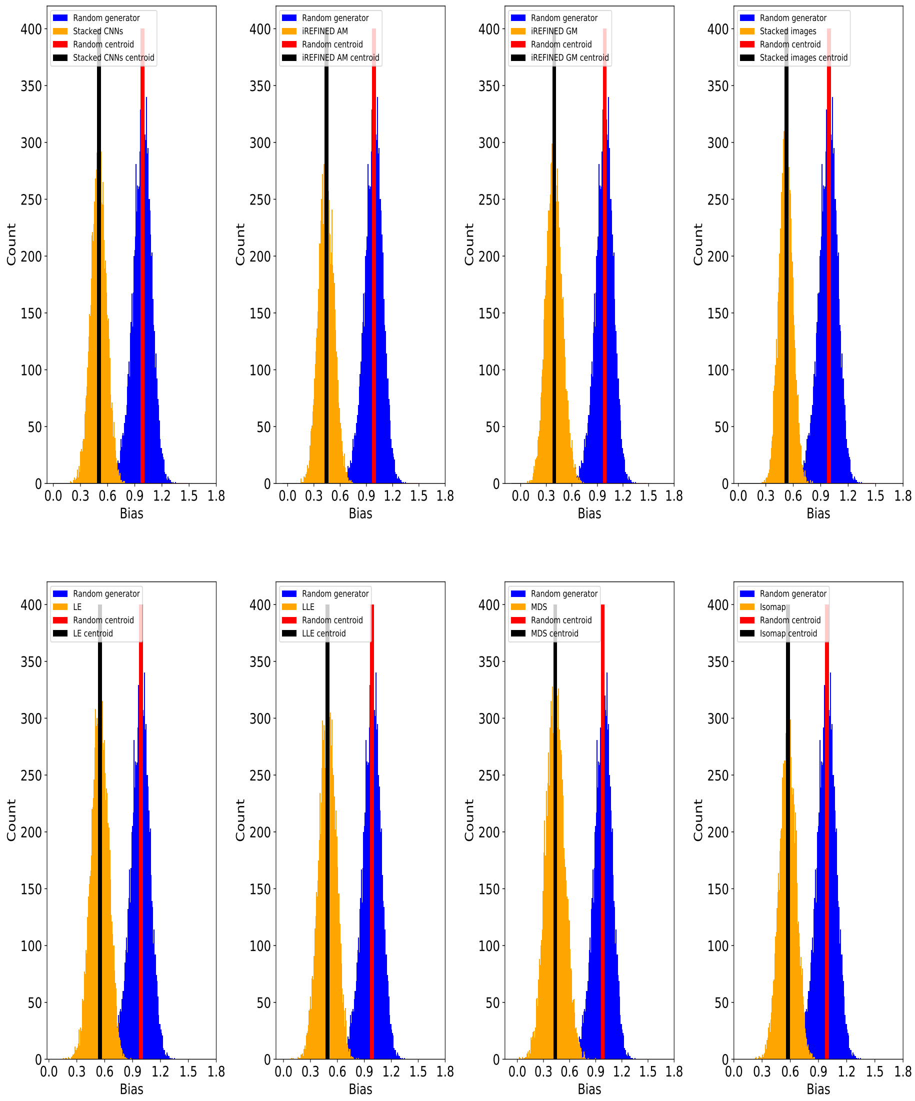

Figure 14: Distribution of Bias of all eight models drawn from the Gap statistics test for the 780_0 cell line of the NCI60 dataset. The distributions clustered into two groups and their associated cluster centroids are shown with a vertical bar on the histogram plots.

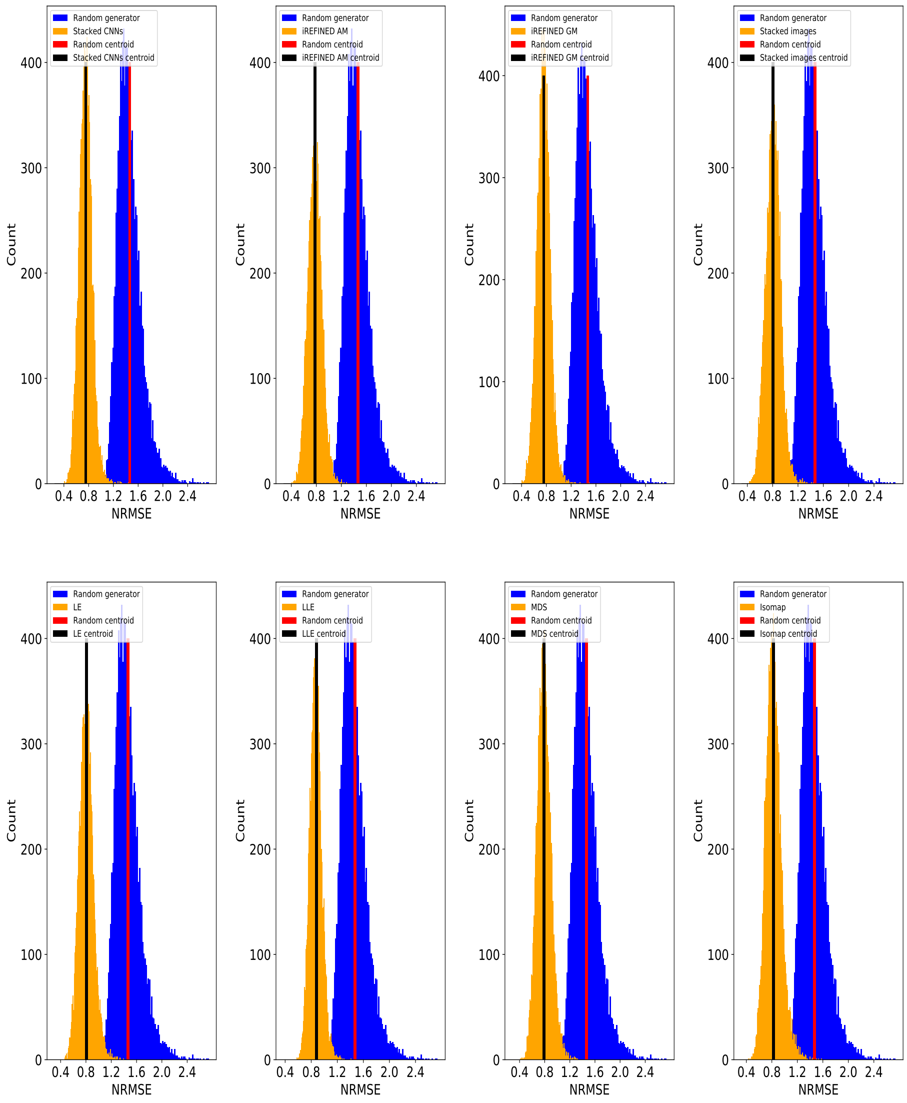

Figure 15: Distribution of NRMSE of all eight models drawn from the Gap statistics test for the SNB_78 cell line of the NCI60 dataset. The distributions clustered into two groups and their associated cluster centroids are shown with a vertical bar on the histogram plots.

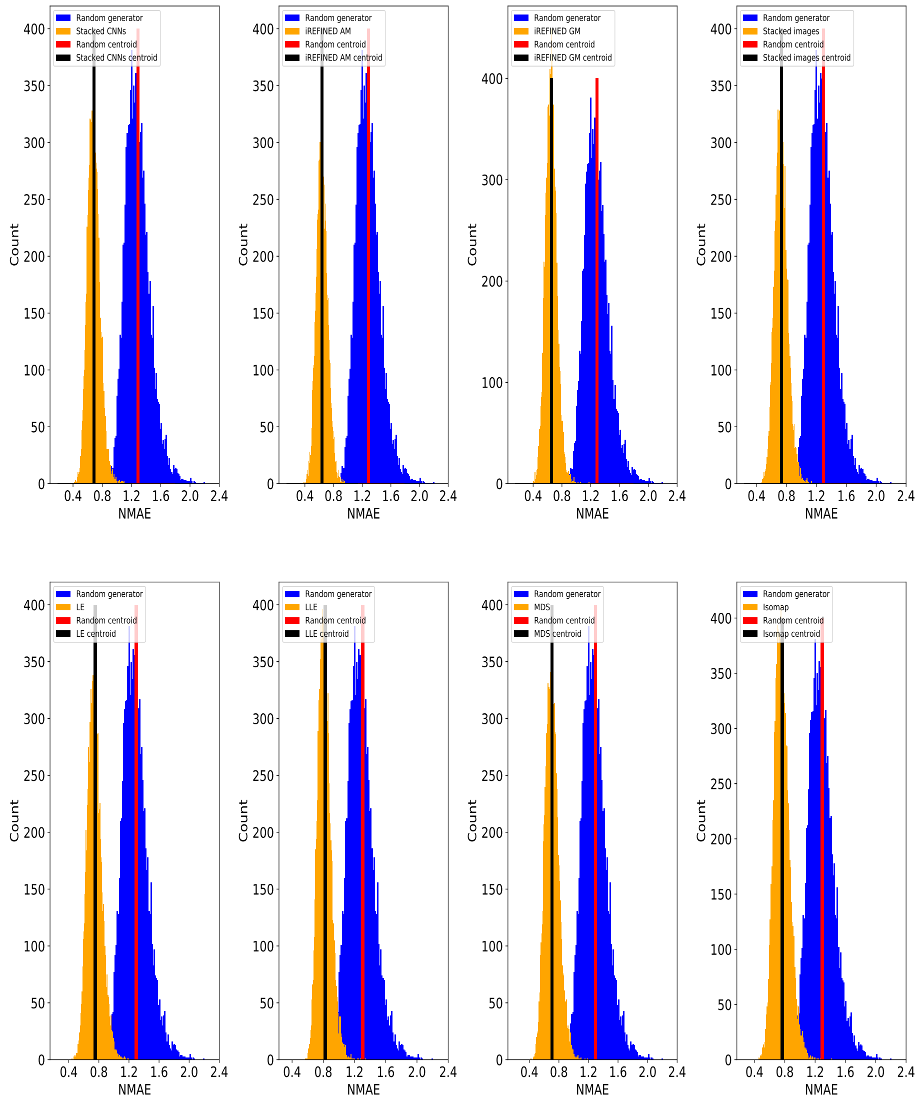

Figure 16: Distribution of NMAE of all eight models drawn from the Gap statistics test for the SNB_78 cell line of the NCI60 dataset. The distributions clustered into two groups and their associated cluster centroids are shown with a vertical bar on the histogram plots.

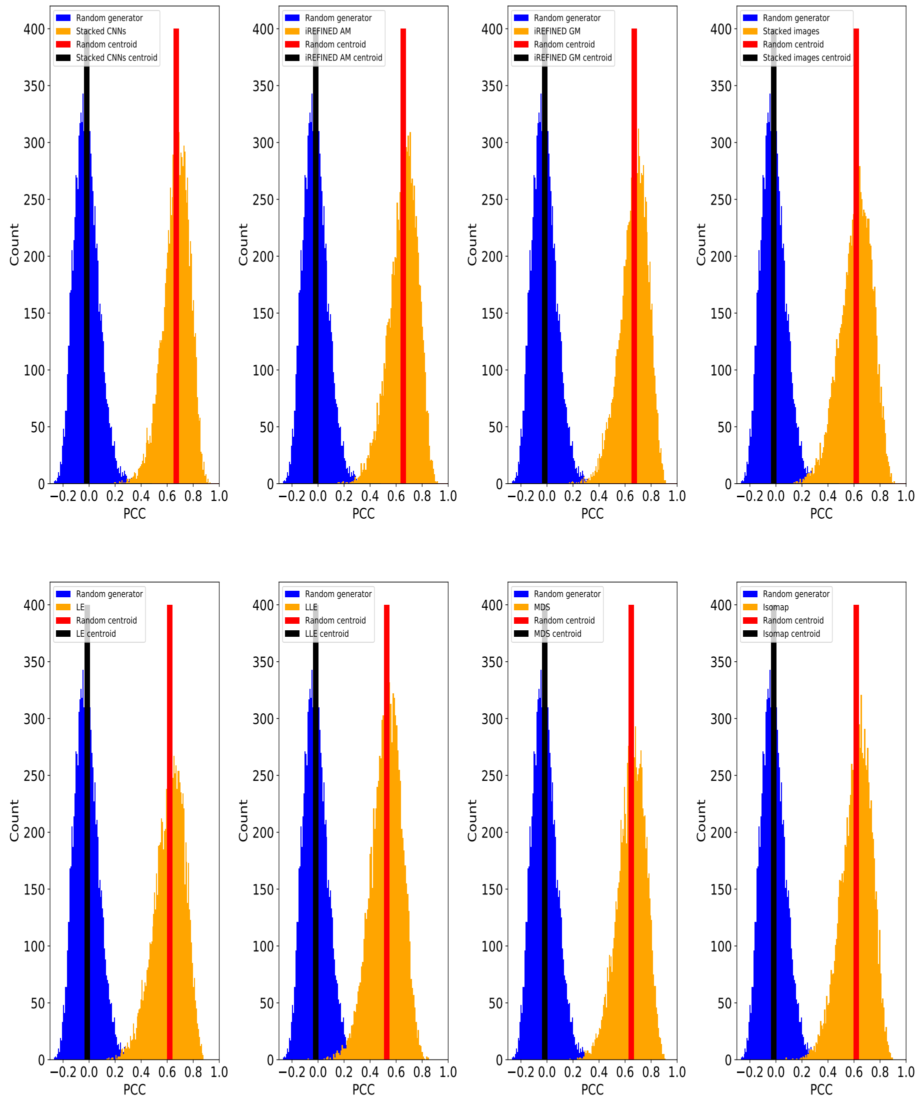

Figure 17: Distribution of PCC of all eight models drawn from the Gap statistics test for the SNB_78 cell line of the NCI60 dataset. The distributions clustered into two groups and their associated cluster centroids are shown with a vertical bar on the histogram plots.

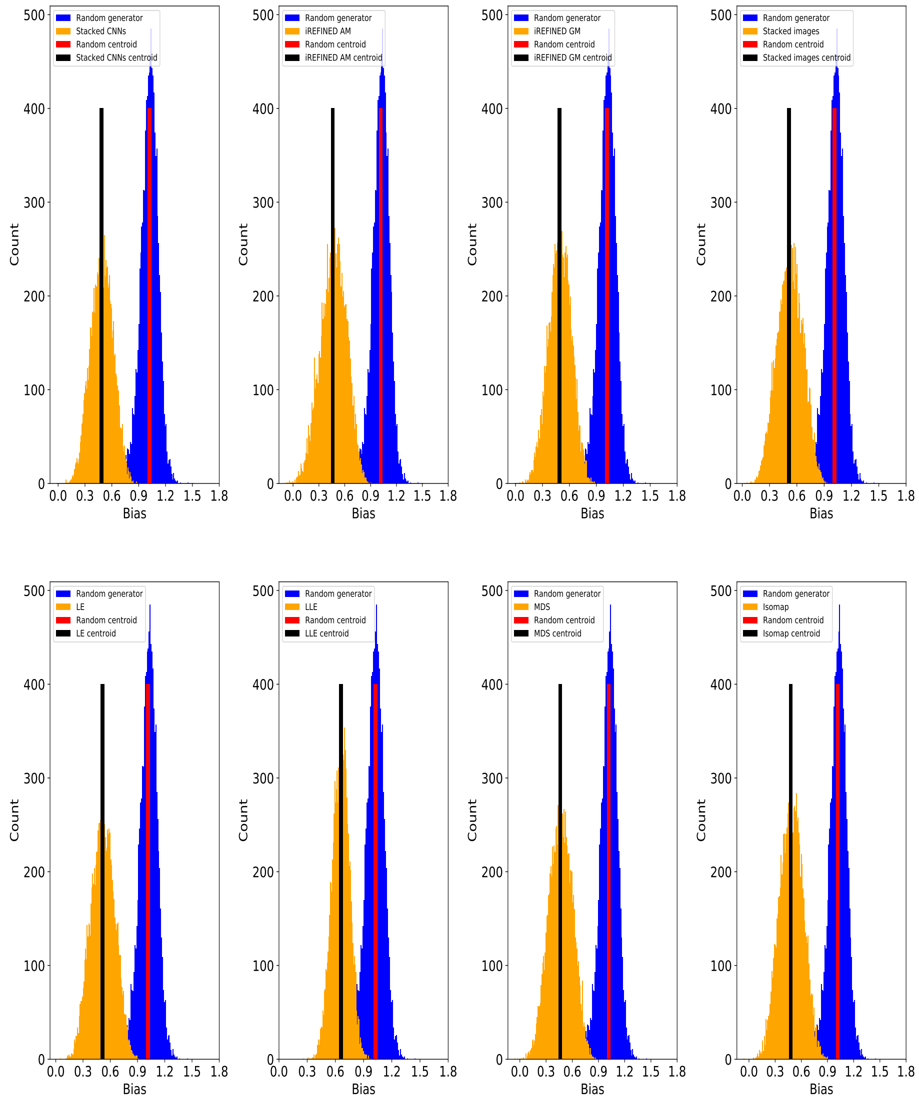

Figure 18: Distribution of Bias of all eight models drawn from the Gap statistics test for the SNB_78 cell line of the NCI60 dataset. The distributions clustered into two groups and their associated cluster centroids are shown with a vertical bar on the histogram plots.

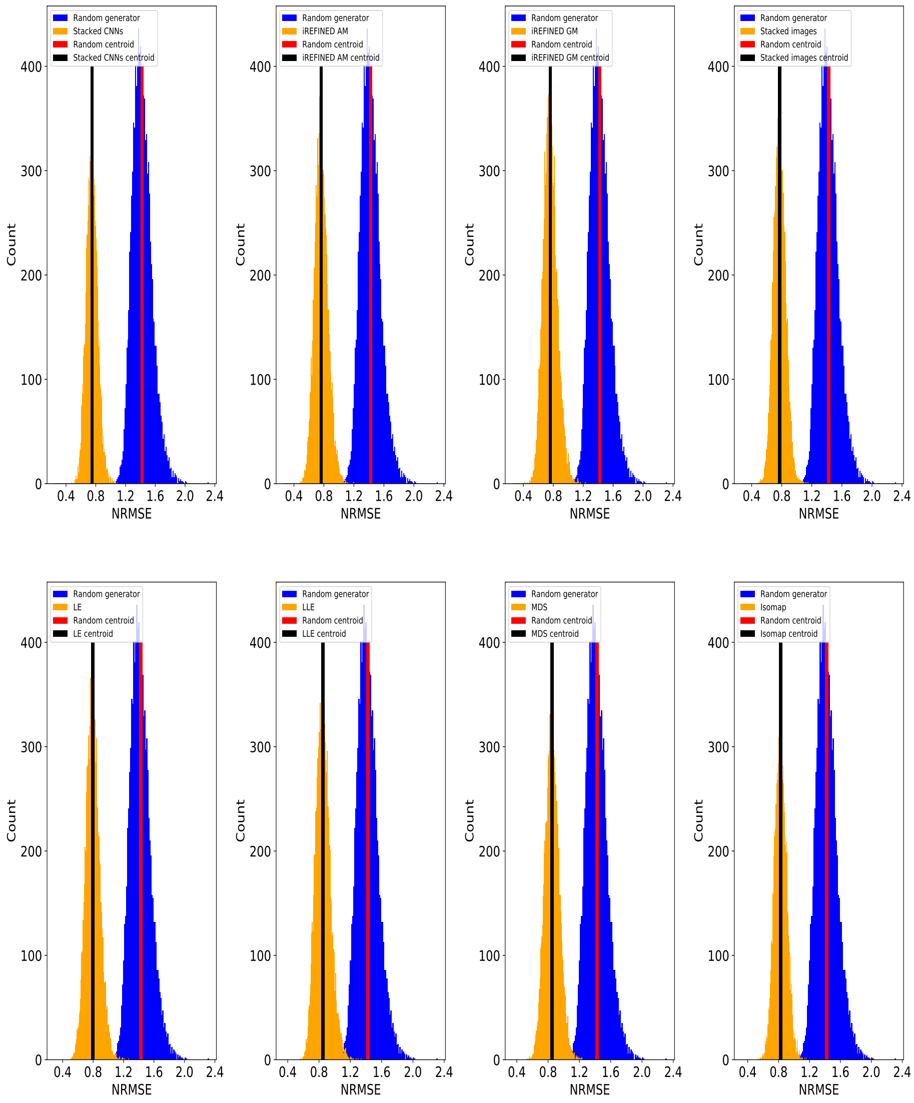

Figure 19: Distribution of NRMSE of all eight models drawn from the Gap statistics test for the BT_549 cell line of the NCI60 dataset. The distributions clustered into two groups and their associated cluster centroids are shown with a vertical bar on the histogram plots.

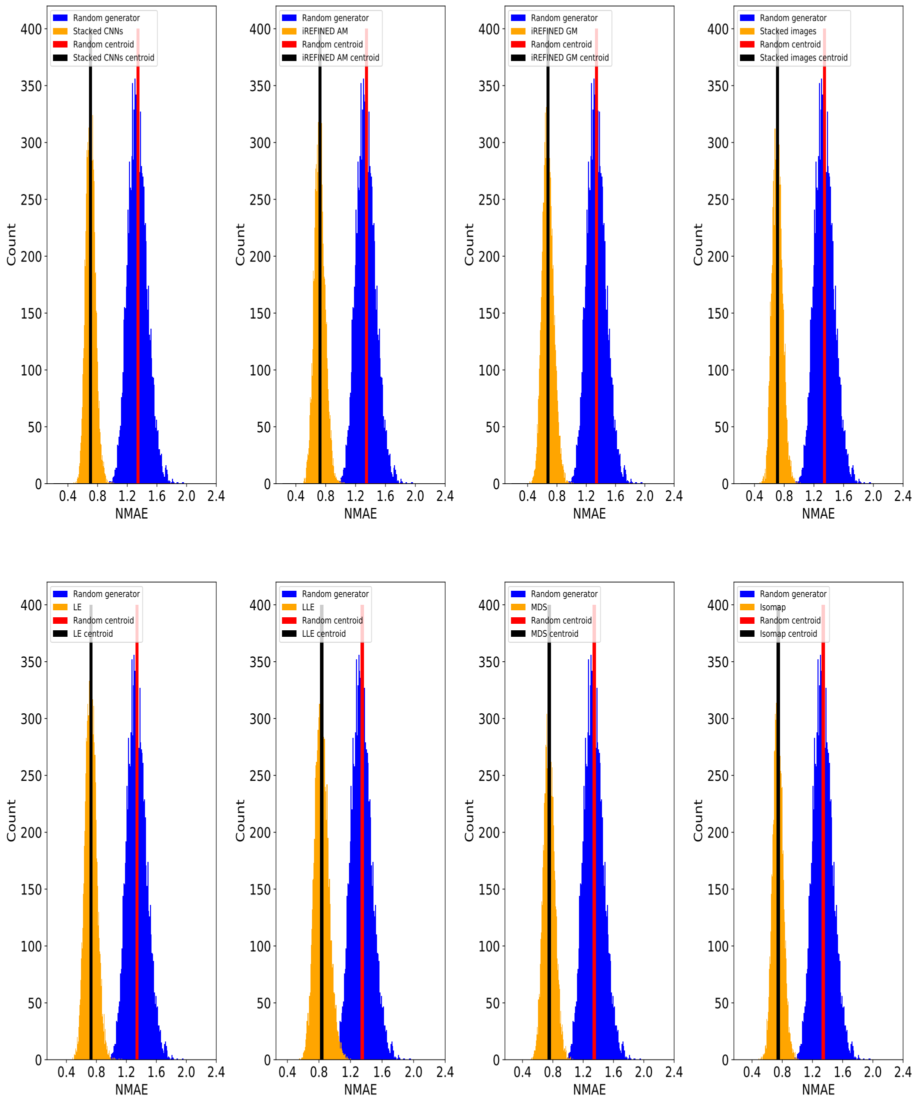

Figure 20: Distribution of NMAE of all eight models drawn from the Gap statistics test for the BT_549 cell line of the NCI60 dataset. The distributions clustered into two groups and their associated cluster centroids are shown with a vertical bar on the histogram plots.

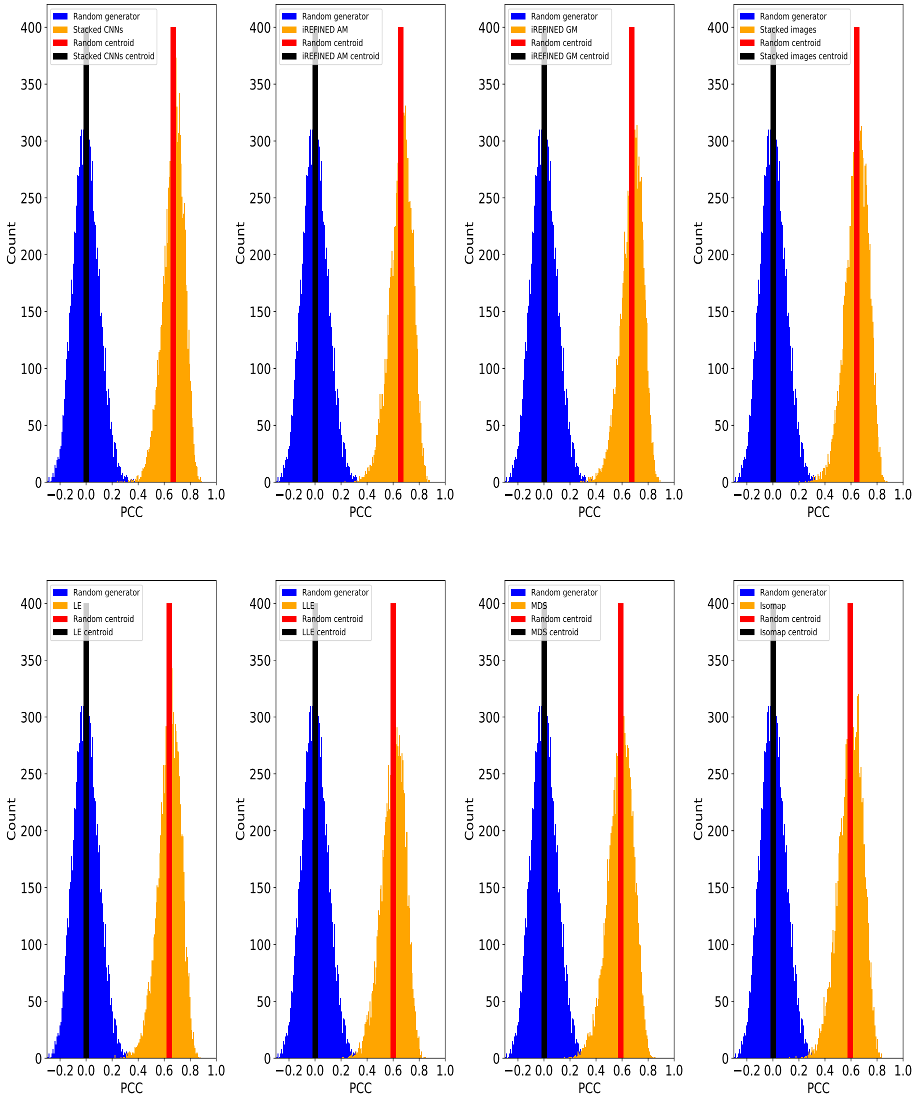

Figure 21: Distribution of PCC of all eight models drawn from the Gap statistics test for the BT_549 cell line of the NCI60 dataset. The distributions clustered into two groups and their associated cluster centroids are shown with a vertical bar on the histogram plots.

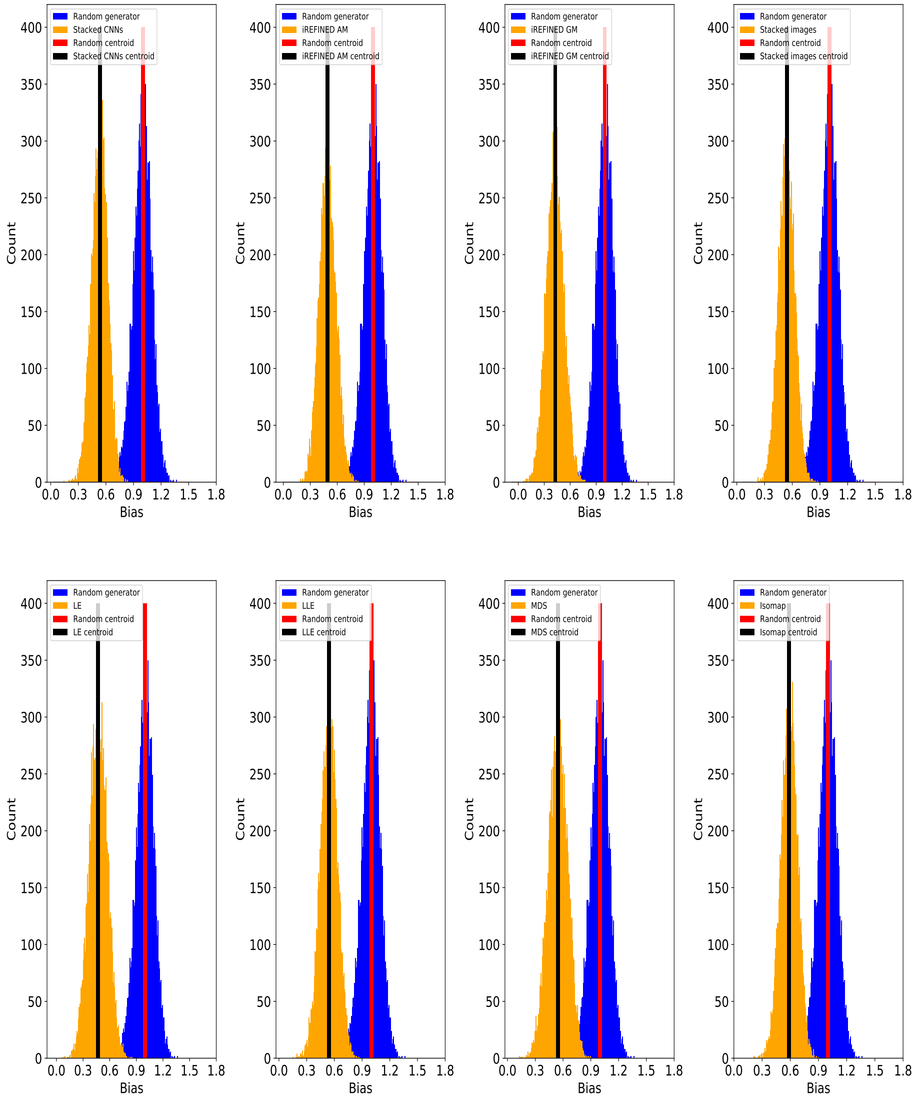

Figure 22: Distribution of Bias of all eight models drawn from the Gap statistics test for the BT_549 cell line of the NCI60 dataset. The distributions clustered into two groups and their associated cluster centroids are shown with a vertical bar on the histogram plots.

Table 8: KL distances between distribution of observed distance matrix and the distance matrix calculated after each projection

Mapping	KL distance (regular scale)	KL distance (log scale)
Isomap	0.1281	0.1617
MDS	0.0180	0.0302
LLE	0.6208	0.3635
LE	0.6336	0.5855

Table 9: NCI60 dataset drug sensitivity prediction using eight single REFINED CNN based regression models. The NRMSE, NMAE, PCC, and bias of each model is used for comparison

Cell lines	sREFINED with Isomap				sREFINED with LE				sREFINED with LLE				sREFINED with MDS			
	NRMSE	NMAE	PCC	Bias	NRMSE	NMAE	PCC	Bias	NRMSE	NMAE	PCC	Bias	NRMSE	NMAE	PCC	Bias
HCC_2998	0.760	0.682	0.660	0.502	0.787	0.725	0.657	0.432	0.773	0.741	0.642	0.490	0.776	0.710	0.656	0.452
MDA_MB_435	0.812	0.729	0.610	0.562	0.839	0.811	0.613	0.499	0.853	0.838	0.591	0.517	0.804	0.756	0.625	0.500
SNB_78	0.814	0.746	0.621	0.489	0.795	0.731	0.624	0.519	0.853	0.798	0.533	0.666	0.776	0.692	0.651	0.474
NCI_ADR_RES	0.842	0.771	0.619	0.433	0.835	0.749	0.603	0.627	0.884	0.889	0.569	0.516	0.808	0.735	0.611	0.533
DU_145	0.784	0.708	0.647	0.464	0.795	0.718	0.626	0.522	0.786	0.747	0.601	0.516	0.780	0.688	0.647	0.506
786_0	0.790	0.689	0.667	0.570	0.779	0.697	0.654	0.543	0.801	0.772	0.620	0.475	0.779	0.698	0.659	0.432
A498	0.790	0.741	0.647	0.466	0.751	0.685	0.671	0.473	0.776	0.707	0.658	0.442	0.783	0.714	0.649	0.460
A549_ATCC	0.735	0.652	0.689	0.457	0.761	0.694	0.687	0.389	0.741	0.717	0.685	0.484	0.732	0.644	0.695	0.445
ACHN	0.794	0.718	0.627	0.524	0.767	0.711	0.652	0.501	0.752	0.679	0.664	0.508	0.725	0.677	0.695	0.452
BT_549	0.819	0.739	0.597	0.586	0.789	0.721	0.642	0.469	0.828	0.813	0.602	0.540	0.844	0.746	0.589	0.545
CAKI_1	0.796	0.759	0.635	0.492	0.786	0.707	0.633	0.571	0.885	0.896	0.599	0.539	0.767	0.703	0.659	0.469
DLD_1	0.795	0.722	0.645	0.567	0.759	0.693	0.660	0.494	0.719	0.651	0.666	0.557	0.739	0.669	0.679	0.483
DMS_114	0.739	0.677	0.684	0.492	0.792	0.734	0.658	0.514	0.773	0.794	0.628	0.549	0.761	0.685	0.665	0.506
DMS_273	0.745	0.726	0.678	0.483	0.789	0.757	0.651	0.456	0.727	0.657	0.700	0.433	0.764	0.750	0.654	0.543
CCRF_CEM	0.780	0.703	0.639	0.552	0.803	0.704	0.631	0.576	0.753	0.720	0.622	0.513	0.818	0.721	0.618	0.584
COLO_205	0.755	0.670	0.682	0.414	0.759	0.678	0.663	0.489	0.764	0.698	0.654	0.425	0.774	0.714	0.672	0.413
EKVX	0.827	0.739	0.602	0.595	0.812	0.730	0.617	0.497	0.841	0.784	0.588	0.522	0.803	0.746	0.621	0.507
Average	0.787	0.716	0.644	0.509	0.788	0.720	0.644	0.504	0.795	0.759	0.625	0.511	0.778	0.709	0.650	0.488

Table 10: NCI60 dataset drug sensitivity prediction using proposed ensemble learning REFINED CNN based regression models. The NRMSE, NMAE, PCC, and bias of each model is used for comparison

Cell lines	REFINED-CNN image stacking				REFI NED-CNN model stacking				iREFINED-CNN-AM				iREFINED-CNN-GM			
	NRMSE	NMAE	PCC	Bias	NRMSE	NMAE	PCC	Bias	NRMSE	NMAE	PCC	Bias	NRMSE	NMAE	PCC	Bias
HCC_2998	0.763	0.678	0.663	0.489	0.683	0.655	0.724	0.510	0.715	0.603	0.711	0.455	0.708	0.623	0.716	0.447
MDA_MB_435	0.795	0.667	0.635	0.561	0.731	0.673	0.685	0.525	0.696	0.633	0.700	0.459	0.726	0.640	0.696	0.419
SNB_78	0.759	0.714	0.656	0.522	0.735	0.662	0.631	0.467	0.760	0.630	0.668	0.447	0.746	0.645	0.675	0.488
NCI_ADR_RES	0.838	0.692	0.588	0.614	0.743	0.681	0.662	0.505	0.731	0.651	0.693	0.490	0.749	0.666	0.679	0.516
DU_145	0.793	0.674	0.628	0.566	0.688	0.657	0.677	0.471	0.725	0.633	0.695	0.505	0.710	0.644	0.708	0.450
786_0	0.730	0.666	0.688	0.512	0.698	0.642	0.763	0.503	0.694	0.600	0.724	0.443	0.691	0.609	0.739	0.389
A498	0.765	0.668	0.668	0.437	0.708	0.657	0.694	0.520	0.714	0.659	0.705	0.462	0.718	0.625	0.704	0.415
A549_ATCC	0.727	0.621	0.694	0.467	0.664	0.607	0.751	0.417	0.693	0.577	0.746	0.418	0.692	0.591	0.730	0.394
ACHN	0.733	0.654	0.693	0.529	0.670	0.645	0.762	0.446	0.659	0.593	0.749	0.383	0.693	0.614	0.732	0.379
BT_549	0.844	0.703	0.615	0.475	0.723	0.691	0.692	0.520	0.745	0.707	0.667	0.489	0.761	0.669	0.680	0.418
CAKI_1	0.780	0.654	0.639	0.511	0.707	0.650	0.716	0.504	0.703	0.636	0.714	0.453	0.720	0.645	0.703	0.446
DLD_1	0.788	0.669	0.656	0.581	0.682	0.631	0.739	0.475	0.730	0.623	0.703	0.496	0.700	0.611	0.727	0.448
DMS_114	0.771	0.764	0.667	0.430	0.684	0.646	0.730	0.465	0.740	0.653	0.695	0.467	0.750	0.680	0.683	0.558
DMS_273	0.797	0.740	0.661	0.547	0.705	0.619	0.707	0.493	0.717	0.593	0.713	0.458	0.751	0.628	0.692	0.526
CCRF_CEM	0.772	0.665	0.652	0.492	0.725	0.669	0.697	0.521	0.727	0.667	0.686	0.491	0.734	0.644	0.683	0.470
COLO_205	0.745	0.630	0.680	0.446	0.671	0.630	0.733	0.466	0.675	0.599	0.742	0.449	0.676	0.579	0.756	0.367
EKVX	0.777	0.691	0.649	0.475	0.717	0.679	0.713	0.507	0.738	0.647	0.682	0.475	0.751	0.680	0.682	0.458
Average	0.775	0.679	0.655	0.509	0.702	0.653	0.710	0.489	0.715	0.630	0.706	0.461	0.722	0.635	0.705	0.446

Table 11: NCI60 dataset drug sensitivity prediction using state-of-the-art models. The NRMSE, NMAE, PCC, and bias of each model is used for comparison.

Cell lines	RF				SVR				EN				XGBoost				KBMTL			
	NRMSE	NMAE	PCC	Bias	NRMSE	NMAE	PCC	Bias	NRMSE	NMAE	PCC	Bias	NRMSE	NMAE	PCC	Bias	NRMSE	NMAE	PCC	Bias
CCRF_CEM	0.893	0.857	0.465	0.839	0.874	0.810	0.521	0.750	0.978	0.943	0.259	0.973	0.856	0.818	0.492	0.804	0.861	0.772	0.543	0.729
COLO_205	0.892	0.856	0.467	0.837	0.867	0.804	0.535	0.746	0.974	0.939	0.288	0.968	0.855	0.817	0.495	0.802	0.854	0.765	0.556	0.724
DU_145	0.903	0.864	0.434	0.838	0.882	0.816	0.507	0.773	0.976	0.941	0.268	0.970	0.866	0.826	0.462	0.803	0.869	0.779	0.529	0.752
EKVX	0.904	0.865	0.433	0.842	0.881	0.815	0.503	0.769	0.978	0.943	0.252	0.972	0.867	0.827	0.461	0.807	0.867	0.778	0.524	0.748
HCC_2998	0.880	0.846	0.488	0.815	0.858	0.796	0.542	0.740	0.968	0.934	0.312	0.961	0.842	0.806	0.515	0.780	0.844	0.757	0.563	0.719
MDA_MB_435	0.912	0.872	0.412	0.849	0.897	0.828	0.477	0.781	0.982	0.946	0.234	0.977	0.875	0.834	0.439	0.814	0.884	0.792	0.498	0.759
SNB_78	0.842	0.816	0.558	0.769	0.847	0.788	0.555	0.745	0.964	0.931	0.352	0.958	0.805	0.775	0.585	0.734	0.833	0.747	0.577	0.723
NCI_ADR_RES	0.908	0.868	0.434	0.860	0.903	0.833	0.476	0.799	0.984	0.948	0.275	0.981	0.871	0.830	0.462	0.825	0.889	0.797	0.497	0.777
786_0	0.887	0.851	0.481	0.832	0.878	0.813	0.521	0.767	0.974	0.939	0.289	0.968	0.849	0.812	0.509	0.796	0.865	0.775	0.542	0.746
A498	0.890	0.854	0.465	0.827	0.859	0.798	0.551	0.750	0.972	0.938	0.312	0.967	0.853	0.815	0.493	0.791	0.846	0.758	0.572	0.729
A549_ATCC	0.870	0.838	0.511	0.806	0.857	0.796	0.555	0.743	0.969	0.935	0.308	0.962	0.832	0.798	0.539	0.771	0.843	0.756	0.577	0.722
ACHN	0.880	0.846	0.494	0.822	0.868	0.805	0.529	0.759	0.975	0.940	0.284	0.970	0.842	0.806	0.521	0.787	0.855	0.766	0.550	0.737
BT_549	0.888	0.852	0.478	0.832	0.870	0.807	0.514	0.755	0.977	0.942	0.279	0.972	0.850	0.813	0.506	0.797	0.857	0.768	0.536	0.734
CAKI_1	0.901	0.863	0.444	0.845	0.885	0.818	0.507	0.773	0.982	0.946	0.241	0.977	0.864	0.825	0.471	0.810	0.872	0.781	0.529	0.751
DLD_1	0.847	0.820	0.557	0.783	0.867	0.804	0.529	0.763	0.975	0.941	0.270	0.968	0.810	0.779	0.585	0.747	0.854	0.765	0.551	0.741
DMS_114	0.832	0.808	0.568	0.746	0.834	0.778	0.571	0.689	0.985	0.950	0.332	0.958	0.795	0.766	0.595	0.711	0.821	0.736	0.592	0.668
DMS_273	0.829	0.805	0.568	0.735	0.860	0.798	0.538	0.728	0.984	0.949	0.318	0.955	0.792	0.763	0.596	0.700	0.846	0.759	0.560	0.707
Average	0.880	0.846	0.486	0.816	0.870	0.806	0.525	0.755	0.976	0.942	0.287	0.968	0.842	0.806	0.513	0.781	0.856	0.768	0.547	0.733

Table 12: NCI-ALMANAC dataset drug sensitivity prediction using single REFINED CNN based regression models. The NRMSE, NMAE, PCC, and bias of each model is used for comparison.

Cell lines	sREFINED with LE				sREFINED with LLE				sREFINED with MDS				sREFINED with Isomap			
	NRMSE	NMAE	PCC	Bias	NRMSE	NMAE	PCC	Bias	NRMSE	NMAE	PCC	Bias	NRMSE	NMAE	PCC	Bias
786-0	0.700	0.657	0.817	0.356	0.864	0.859	0.816	0.416	0.749	0.736	0.724	0.662	0.794	0.818	0.777	0.440
A498	0.539	0.479	0.851	0.187	0.500	0.451	0.881	0.300	0.488	0.427	0.881	0.224	0.585	0.564	0.872	0.250
A549/ATCC	0.408	0.360	0.921	0.262	0.442	0.394	0.933	0.316	0.420	0.377	0.915	0.230	0.391	0.341	0.924	0.217
ACHN	0.434	0.380	0.908	0.246	0.397	0.347	0.930	0.225	0.416	0.367	0.920	0.282	0.380	0.331	0.927	0.191
CCRF-CEM	0.436	0.388	0.911	0.160	0.406	0.347	0.925	0.135	0.405	0.351	0.918	0.148	0.438	0.385	0.905	0.274
COLO205	0.434	0.389	0.913	0.143	0.515	0.499	0.909	0.078	0.432	0.371	0.908	0.256	0.411	0.356	0.915	0.176
DU-145	0.420	0.369	0.913	0.235	0.436	0.408	0.917	0.293	0.406	0.354	0.914	0.166	0.495	0.435	0.905	0.207
EKVX	0.488	0.443	0.876	0.297	0.494	0.452	0.874	0.251	0.489	0.444	0.874	0.276	0.481	0.432	0.877	0.216
HCC-2998	0.475	0.438	0.883	0.194	0.692	0.704	0.854	0.469	0.504	0.468	0.874	0.244	0.467	0.425	0.885	0.188
HCT-15	0.491	0.447	0.896	0.232	0.464	0.418	0.922	0.173	0.459	0.392	0.890	0.207	0.408	0.347	0.914	0.149
HCT-116	0.502	0.438	0.873	0.250	0.694	0.649	0.818	0.293	1.204	1.287	0.782	0.165	0.681	0.653	0.861	0.384
HL-60(TB)	0.465	0.428	0.891	0.294	0.460	0.431	0.904	0.200	0.447	0.403	0.903	0.264	0.512	0.448	0.899	0.220
HOP-62	0.398	0.345	0.921	0.214	0.438	0.396	0.913	0.271	0.515	0.487	0.908	0.137	0.429	0.371	0.908	0.233
HOP-92	0.447	0.398	0.897	0.222	0.512	0.480	0.896	0.412	0.421	0.387	0.913	0.252	0.427	0.370	0.915	0.112
HT29	0.499	0.424	0.868	0.217	0.522	0.480	0.864	0.289	0.496	0.431	0.873	0.243	0.514	0.447	0.872	0.167
IGROV1	0.469	0.423	0.885	0.262	0.459	0.409	0.885	0.237	0.487	0.421	0.875	0.258	0.454	0.394	0.895	0.275
K-562	0.496	0.444	0.887	0.271	0.479	0.416	0.900	0.269	0.468	0.413	0.888	0.288	0.582	0.599	0.897	0.134
KM12	0.785	0.774	0.753	0.370	0.840	0.839	0.696	0.666	0.654	0.612	0.757	0.428	0.869	0.890	0.762	0.395
LOXIMVI	0.413	0.382	0.920	0.223	0.395	0.343	0.933	0.191	0.371	0.331	0.929	0.153	0.403	0.373	0.921	0.110
MALME-3M	0.477	0.453	0.892	0.124	0.439	0.408	0.906	0.189	0.457	0.422	0.899	0.297	0.448	0.421	0.908	0.194
Average	0.489	0.443	0.884	0.238	0.522	0.486	0.884	0.284	0.514	0.474	0.877	0.259	0.508	0.470	0.887	0.227

Table 13: NCI-ALMANAC dataset drug sensitivity prediction using proposed ensemble learning REFINED CNN based regression models. The NRMSE, NMAE, PCC, and bias of each model is used for comparison.

Cell lines	REFINED-CNN model stacking				iREFINED-CNN-GM				iREFINED-CNN-AM				REFINED-CNN image stacking			
	NRMSE	NMAE	PCC	Bias	NRMSE	NMAE	PCC	Bias	NRMSE	NMAE	PCC	Bias	NRMSE	NMAE	PCC	Bias
786-0	0.566	0.501	0.835	0.341	0.446	0.483	0.873	0.253	0.691	0.422	0.797	0.562	0.741	0.735	0.775	0.671
A498	0.451	0.386	0.899	0.151	0.404	0.484	0.894	0.257	0.476	0.586	0.893	0.200	0.601	0.552	0.807	0.360
A549/ATCC	0.356	0.292	0.940	0.156	0.445	0.360	0.939	0.219	0.382	0.330	0.927	0.216	0.394	0.328	0.949	0.245
ACHN	0.362	0.301	0.935	0.123	0.398	0.331	0.917	0.177	0.398	0.289	0.935	0.237	0.378	0.341	0.925	0.187
CCRF-CEM	0.374	0.301	0.944	0.088	0.429	0.349	0.929	0.136	0.384	0.340	0.938	0.202	0.383	0.345	0.920	0.191
COLO205	0.369	0.315	0.933	0.164	0.537	0.336	0.922	0.226	0.411	0.372	0.929	0.216	0.469	0.444	0.913	0.347
DU-145	0.390	0.327	0.927	0.170	0.514	0.393	0.888	0.270	0.412	0.370	0.928	0.244	0.446	0.387	0.911	0.164
EKVX	0.450	0.401	0.893	0.171	0.520	0.420	0.869	0.303	0.580	0.423	0.886	0.264	0.502	0.444	0.880	0.338
HCC-2998	0.461	0.396	0.895	0.149	0.399	0.439	0.867	0.369	0.472	0.570	0.882	0.194	0.705	0.660	0.856	0.364
HCT-15	0.457	0.321	0.889	0.190	0.535	0.502	0.922	0.207	0.424	0.360	0.931	0.183	1.121	1.149	0.887	0.376
HCT-116	0.384	0.391	0.925	0.120	0.486	0.493	0.869	0.313	0.646	0.503	0.791	0.423	0.485	0.438	0.702	0.525
HL-60(TB)	0.389	0.342	0.923	0.118	0.399	0.411	0.884	0.171	0.492	0.349	0.893	0.334	0.453	0.419	0.904	0.261
HOP-62	0.367	0.290	0.926	0.141	0.439	0.344	0.922	0.191	0.418	0.373	0.928	0.266	0.452	0.386	0.911	0.283
HOP-92	0.377	0.329	0.934	0.113	0.503	0.369	0.911	0.130	0.428	0.663	0.937	0.301	0.515	0.508	0.871	0.382
HT29	0.471	0.389	0.884	0.190	0.479	0.531	0.873	0.279	0.503	0.416	0.871	0.299	0.563	0.534	0.881	0.310
IGROV1	0.422	0.351	0.916	0.177	0.505	0.425	0.892	0.242	0.489	0.385	0.891	0.215	0.553	0.504	0.870	0.420
K-562	0.419	0.370	0.912	0.181	0.691	0.451	0.880	0.315	0.463	0.408	0.903	0.193	0.477	0.400	0.913	0.243
KM12	0.613	0.572	0.782	0.359	0.427	0.682	0.750	0.551	0.689	0.712	0.750	0.506	0.940	0.965	0.480	0.864
LOXIMVI	0.328	0.291	0.942	0.113	0.451	0.322	0.931	0.137	0.401	0.376	0.932	0.280	0.583	0.531	0.853	0.430
MALME-3M	0.401	0.354	0.913	0.146	0.478	0.424	0.900	0.218	0.428	0.381	0.910	0.173	0.450	0.406	0.915	0.282
Average	0.420	0.361	0.907	0.168	0.474	0.427	0.892	0.248	0.479	0.431	0.893	0.275	0.561	0.524	0.856	0.362

Table 14: NCI-ALMANAC dataset drug sensitivity prediction using state-of-the-art models. The NRMSE, NMAE, PCC, and bias of each model is used for comparison.

Cell lines	Deep Synergy				Gradient Boosting				Xie et al.				RF				SVR				EN			
	NRMSE	NMAE	PCC	Bias	NRMSE	NMAE	PCC	Bias	NRMSE	NMAE	PCC	Bias	NRMSE	MAE	PCC	Bias	NRMSE	MAE	PCC	Bias	NRMSE	MAE	PCC	Bias
786-0	1.031	1.02	0.095	0.967	0.552	0.687	0.842	0.373	1.287	1.179	0.41	0.991	0.545	0.689	0.84	0.322	0.612	0.684	0.797	0.342	0.653	0.777	0.771	0.484
A498	1.057	1.025	0.117	0.944	0.501	0.673	0.868	0.3	1.488	1.293	0.511	0.991	0.508	0.670	0.861	0.241	0.575	0.684	0.825	0.236	0.647	0.786	0.763	0.435
A549/ATCC	1.084	1.047	0.112	0.959	0.534	0.691	0.852	0.361	1.822	1.399	0.475	0.993	0.511	0.657	0.86	0.28	0.556	0.660	0.831	0.301	0.602	0.746	0.806	0.439
ACHN	1.029	1.024	0.234	0.916	0.482	0.654	0.884	0.313	1.519	1.258	0.542	0.986	0.493	0.668	0.874	0.306	0.472	0.620	0.883	0.193	0.553	0.715	0.838	0.368
CCRF-CEM	0.985	0.992	0.221	0.923	0.474	0.646	0.883	0.263	1.486	1.238	0.55	0.985	0.495	0.655	0.869	0.249	0.487	0.647	0.875	0.192	0.584	0.731	0.813	0.374
COLO 205	1.009	1.003	0.136	0.957	0.509	0.672	0.862	0.295	1.444	1.213	0.529	0.984	0.498	0.660	0.867	0.259	0.536	0.662	0.849	0.205	0.619	0.751	0.786	0.412
DU-145	0.97	0.983	0.299	0.903	0.518	0.674	0.858	0.321	1.466	1.222	0.563	0.99	0.541	0.678	0.842	0.31	0.566	0.663	0.829	0.253	0.616	0.747	0.791	0.42
EKVX	1.01	1.017	0.328	0.836	0.577	0.718	0.819	0.382	1.42	1.26	0.435	0.995	0.574	0.717	0.819	0.343	0.594	0.710	0.806	0.314	0.634	0.762	0.777	0.457
HCC-2998	1.274	1.151	0.011	0.994	0.556	0.711	0.839	0.33	1.387	1.211	0.403	0.992	0.554	0.702	0.834	0.287	0.564	0.697	0.832	0.226	0.634	0.773	0.776	0.431
HCT-15	1.342	1.155	0.078	0.972	0.514	0.67	0.866	0.351	1.457	1.22	0.472	0.993	0.504	0.681	0.865	0.293	0.53	0.675	0.849	0.249	0.639	0.763	0.773	0.466
HCT-116	1.254	1.118	0.154	0.904	0.513	0.663	0.864	0.326	1.913	1.43	0.398	0.993	0.552	0.662	0.834	0.315	0.582	0.648	0.819	0.253	0.632	0.766	0.779	0.443
HL-60(TB)	1.008	0.999	0.144	0.951	0.562	0.698	0.828	0.34	1.718	1.326	0.504	0.987	0.59	0.719	0.808	0.323	0.619	0.710	0.793	0.287	0.623	0.766	0.783	0.411
HOP-62	0.99	0.985	0.206	0.935	0.473	0.646	0.888	0.309	1.548	1.266	0.52	0.992	0.485	0.653	0.877	0.291	0.517	0.658	0.856	0.256	0.578	0.729	0.825	0.414
HOP-92	1.139	1.083	0.208	0.932	0.49	0.672	0.877	0.308	1.846	1.419	0.313	0.996	0.481	0.662	0.877	0.243	0.509	0.657	0.863	0.212	0.579	0.740	0.822	0.409
HT29	1.022	1.019	0.167	0.936	0.534	0.696	0.852	0.342	1.896	1.431	0.165	0.998	0.537	0.690	0.844	0.312	0.573	0.684	0.822	0.276	0.623	0.770	0.785	0.423
IGROV1	1.194	1.119	0.137	0.955	0.549	0.699	0.839	0.332	1.635	1.331	0.537	0.989	0.55	0.670	0.837	0.285	0.664	0.697	0.767	0.285	0.661	0.759	0.753	0.415
K-562	1.338	1.207	0.292	0.857	0.539	0.696	0.845	0.347	1.367	1.213	0.543	0.991	0.547	0.699	0.837	0.307	0.533	0.680	0.847	0.255	0.623	0.768	0.789	0.457
KM12	1.161	1.079	0.193	0.897	0.558	0.728	0.834	0.361	1.899	1.456	-0.147	1.002	0.566	0.724	0.824	0.32	0.664	0.739	0.763	0.302	0.688	0.815	0.727	0.496
LOX IMVI	1.219	1.118	0.269	0.893	0.436	0.64	0.908	0.278	1.442	1.238	0.489	0.988	0.463	0.647	0.887	0.253	0.481	0.640	0.88	0.167	0.54	0.723	0.845	0.349
MALME-3M	1.072	1.008	0.113	0.955	0.491	0.671	0.875	0.303	1.445	1.29	0.479	0.992	0.515	0.681	0.858	0.27	0.578	0.681	0.818	0.286	0.636	0.771	0.775	0.449
Average	1.109	1.058	0.176	0.929	0.518	0.68	0.859	0.327	1.574	1.295	0.435	0.991	0.525	0.679	0.851	0.29	0.561	0.675	0.83	0.255	0.618	0.758	0.789	0.428

Table 15: Robustness analysis to compare iREFINED-AM-CNN (integrated REFINED CNN with arithmetic mean) with 7 other competing models per NRMSE and each cell line of the NCI60 dataset. Each cell of the table represents the percentage for which iREFINED-CNN-AM outperforms the paired competing model.

Cell lines	MDS	Isomap	LLE	LE	REFINED-CNN model stacking	iREFINED-CNN-GM	REFINED-CNN image stacking
786_0	79.15	84.77	86.89	80.40	51.73	50.09	63.07
A498	70.47	74.31	73.55	61.90	48.84	51.80	65.24
A549_ATCC	65.40	65.83	76.54	72.53	47.05	51.99	59.77
ACHN	71.72	88.38	82.18	82.69	58.82	61.96	59.70
BT_549	78.71	72.40	78.05	62.77	46.08	50.98	59.73
CAKI_1	68.95	77.29	96.51	76.33	49.82	53.39	59.64
CCRF_CEM	79.16	67.24	66.43	75.85	45.17	49.87	60.75
COLO_205	79.55	75.29	79.39	76.48	51.85	50.79	54.46
DLD_1	56.95	72.28	57.39	60.58	38.88	42.59	68.48
DMS_114	56.92	52.65	68.08	66.67	40.95	51.42	75.52
DMS_273	69.06	62.59	63.16	73.94	46.08	63.06	80.90
DU_145	69.93	72.12	81.26	75.65	47.31	46.62	55.16
EKVX	72.86	78.44	83.39	73.36	48.97	55.48	57.62
HCC_2998	71.94	67.37	73.75	73.26	46.00	48.48	61.05
MDA_MB_435	80.20	82.83	91.04	88.73	58.52	55.95	54.17
NCI_ADR_RES	76.97	84.13	93.70	85.35	57.31	58.09	55.45
SNB_78	54.36	64.20	75.97	59.09	45.00	45.17	64.60
average	70.72	73.07	78.07	73.27	48.73	52.22	62.08

Table 16: Robustness analysis to compare iREFINED-CNN-AM (integrated REFINED CNN with arithmetic mean) with 7 other competing models per NMAE and each cell line of the NCI60 dataset. Each cell of the table represents the percentage for which iREFINED-CNN-AM outperforms the paired competing model.

Cell line	MDS	Isomap	LLE	LE	REFINED-CNN model stacking	iREFINED-CNN-GM	REFINED-CNN image stacking
786_0	85.32	84.60	96.61	84.87	68.56	55.02	61.45
A498	72.83	82.35	75.09	61.79	51.79	43.21	55.54
A549_ATCC	72.95	76.92	90.74	85.30	63.24	54.15	53.93
ACHN	85.38	94.03	87.35	92.91	78.58	64.22	54.05
BT_549	65.56	62.18	88.21	56.91	46.21	36.94	53.76
CAKI_1	76.72	90.76	101.05	82.06	65.11	57.41	48.87
CCRF_CEM	68.03	60.26	66.69	59.76	49.07	37.10	48.49
COLO_205	87.52	76.71	85.40	80.04	64.50	43.80	48.69
DLD_1	67.52	88.05	68.52	76.37	58.48	45.79	65.49
DMS_114	65.56	60.26	92.40	80.57	56.15	65.82	83.98
DMS_273	92.98	89.22	75.32	93.61	65.59	66.95	84.24
DU_145	69.60	77.28	88.42	78.24	60.49	51.45	55.60
EKVX	77.97	80.73	89.55	73.93	61.13	57.33	53.88
HCC_2998	84.06	75.85	89.39	87.17	67.77	52.01	59.81
MDA_MB_435	88.90	84.36	98.30	97.09	68.15	52.58	44.94
NCI_ADR_RES	79.02	87.45	98.70	83.85	62.24	52.77	55.06
SNB_78	72.95	84.35	95.41	82.83	65.52	56.76	64.99
Average	77.23	79.73	87.48	79.84	61.92	52.55	58.40

Table 17: Robustness analysis to compare iREFINED-CNN-AM (integrated REFINED CNN with arithmetic mean) with 7 other competing models per PCC and each cell line of the NCI60 dataset. Each cell of the table represents the percentage for which iREFINED-CNN-AM outperforms the paired competing model.

Cell lines	MDS	Isomap	LLE	LE	REFINED-CNN model stacking	iREFINED-CNN-GM	REFINED-CNN image stacking
786_0	73.76	70.40	82.78	74.21	51.62	48.00	62.38
A498	66.07	67.70	66.54	60.42	46.22	46.42	57.90
A549_ATCC	63.51	63.25	75.09	65.51	48.66	50.24	58.90
ACHN	72.50	85.78	80.42	81.80	59.53	59.51	57.91
BT_549	71.57	69.83	68.16	59.67	48.33	45.39	58.76
CAKI_1	64.80	74.61	88.62	74.01	52.53	52.04	57.49
CCRF_CEM	72.47	65.19	64.63	67.74	45.15	47.94	58.29
COLO_205	72.58	71.47	76.18	74.80	51.91	48.69	52.23
DLD_1	54.31	62.81	55.86	61.19	38.19	41.04	64.73
DMS_114	60.40	56.03	75.00	61.30	46.11	55.19	67.74
DMS_273	69.87	63.92	59.44	69.45	50.09	59.96	80.22
DU_145	65.20	66.03	78.57	70.73	48.25	43.38	52.90
EKVX	69.52	73.33	79.56	70.00	48.94	52.19	53.35
HCC_2998	70.52	67.12	73.16	68.92	47.62	51.16	59.24
MDA_MB_435	77.34	79.07	85.14	80.79	60.55	52.43	52.50
NCI_ADR_RES	73.46	72.45	85.10	75.36	55.19	54.89	53.20
SNB_78	52.05	60.60	81.62	60.96	46.47	48.03	64.08
Average	67.64	68.80	75.05	69.23	49.73	50.38	59.52

Table 18: Robustness analysis to compare iREFINED-CNN-AM (integrated REFINED CNN with arithmetic mean) with 7 other competing models per Bias and each cell line of the NCI60 dataset. Each cell of the table represents the percentage for which iREFINED-CNN-AM outperforms the paired competing model.

Cell line	MDS	Isomap	LLE	LE	REFINED-CNN model stacking	iREFINED-CNN-GM	REFINED-CNN image stacking
786_0	51.84	83.03	68.65	77.34	70.61	34.44	56.27
A498	45.89	45.98	41.36	49.18	63.24	37.90	49.00
A549_ATCC	61.11	59.87	74.19	38.23	59.39	40.53	37.49
ACHN	72.73	82.38	87.90	76.37	72.19	52.27	32.39
BT_549	61.52	76.40	65.69	43.58	63.93	34.21	50.81
CAKI_1	49.70	56.42	71.33	80.22	67.16	41.81	43.75
CCRF_CEM	78.33	67.90	54.28	65.79	55.55	43.96	46.82
COLO_205	34.18	31.94	42.10	58.12	51.02	27.03	33.18
DLD_1	46.11	66.01	68.93	52.33	50.34	38.69	76.10
DMS_114	57.66	55.96	75.91	64.20	54.02	77.79	43.27
DMS_273	65.53	50.86	39.44	47.07	44.78	61.41	83.02
DU_145	51.27	31.82	52.79	57.11	40.06	29.47	49.50
EKVX	59.21	82.47	62.74	49.21	63.05	45.96	40.10
HCC_2998	47.11	56.72	55.79	35.57	61.48	43.64	24.20
MDA_MB_435	59.46	82.60	73.33	61.25	86.57	38.93	37.50
NCI_ADR_RES	52.59	26.64	54.15	83.72	50.29	57.95	56.33
SNB_78	51.25	50.15	84.60	66.34	51.76	53.36	58.86
Average	55.62	59.24	63.13	59.15	59.14	44.67	48.15

Table 19: Robustness analysis to compare iREFINED-CNN-GM (integrated REFINED CNN with geometric mean) with 7 other competing models per NRMSE and each cell line of the NCI60 dataset. Each cell of the table represents the percentage for which iREFINED-CNN-GM outperforms the paired competing model.

Cell lines	MDS	Isomap	LLE	LE	iREFINED-CNN-AM	REFINED-CNN model stacking	REFINED-CNN image stacking
786_0	78.61	84.29	86.20	77.39	51.27	52.83	62.11
A498	68.43	71.60	71.99	62.12	51.05	48.98	58.94
A549_ATCC	62.79	63.82	75.64	70.43	49.64	46.07	52.15
ACHN	63.03	81.24	74.90	70.91	42.42	46.12	55.53
BT_549	75.02	70.96	75.13	61.51	53.61	48.44	53.32
CAKI_1	65.15	76.42	93.55	74.30	49.76	47.17	56.33
CCRF_CEM	78.58	68.20	66.14	74.82	52.84	46.57	51.22
COLO_205	79.52	74.55	78.91	74.80	52.02	54.38	57.50
DLD_1	66.23	78.83	66.85	71.14	59.54	49.26	64.95
DMS_114	57.17	51.09	68.01	66.89	50.66	38.15	64.60
DMS_273	58.64	51.51	51.73	63.07	40.92	32.08	63.34
DU_145	72.84	76.22	81.30	76.99	58.50	55.05	55.56
EKVX	65.47	72.87	78.63	67.93	46.81	44.39	50.54
HCC_2998	74.50	69.40	74.77	74.44	54.98	47.87	58.41
MDA_MB_435	75.48	76.55	87.57	82.53	46.85	53.25	57.05
NCI_ADR_RES	70.80	75.57	90.72	77.78	44.52	48.93	53.62
SNB_78	59.29	68.35	81.21	63.62	55.72	50.05	65.21
Average	68.92	71.26	76.66	71.22	50.65	47.62	57.67

Table 20: Robustness analysis to compare iREFINED-CNN-GM (integrated REFINED CNN with geometric mean) with 7 other competing models per NMAE and each cell line of the NCI60 dataset. Each cell of the table represents the percentage for which iREFINED-CNN-GM outperforms the paired competing model.

Cell lines	MDS	Isomap	LLE	LE	iREFINED-CNN-AM	REFINED-CNN model stacking	REFINED-CNN image stacking
786_0	81.10	80.03	93.78	82.13	47.72	64.33	72.82
A498	77.68	84.49	79.94	69.68	59.10	60.95	64.11
A549_ATCC	70.75	72.54	89.06	83.66	50.02	60.58	62.11
ACHN	71.86	83.29	72.35	80.74	34.80	61.62	63.39
BT_549	75.78	74.38	91.70	68.12	65.45	60.98	62.22
CAKI_1	70.88	85.51	98.85	73.37	44.47	55.85	53.86
CCRF_CEM	80.12	73.92	76.86	74.75	65.63	63.78	60.49
COLO_205	89.59	80.74	87.32	83.20	59.97	71.32	68.82
DLD_1	70.06	85.31	69.15	76.59	53.90	59.00	71.47
DMS_114	50.52	48.19	84.10	69.20	36.68	39.02	76.17
DMS_273	86.66	80.91	58.43	86.16	35.86	50.20	84.88
DU_145	65.99	72.54	83.25	76.46	48.93	56.49	60.75
EKVX	74.73	73.10	83.50	68.94	46.13	54.48	55.15
HCC_2998	81.99	74.18	88.34	84.60	50.52	67.78	72.62
MDA_MB_435	86.18	80.91	96.24	93.93	49.78	66.27	60.90
NCI_ADR_RES	76.18	83.91	98.11	82.27	49.83	59.53	61.60
SNB_78	68.11	81.46	92.82	78.68	46.54	61.68	74.67
Average	75.19	77.38	84.93	78.38	49.73	59.64	66.24

Table 21: Robustness analysis to compare iREFINED-CNN-GM (integrated REFINED CNN with geometric mean) with 7 other competing models per PCC and each cell line of the NCI60 dataset. Each cell of the table represents the percentage for which iREFINED-CNN-AM outperforms the paired competing model.

Cell lines	MDS	Isomap	LLE	LE	iREFINED-CNN-AM	REFINED-CNN model stacking	REFINED-CNN image stacking
786_0	76.96	70.33	82.54	74.03	53.58	53.38	66.65
A498	68.78	69.63	70.31	61.12	53.89	50.46	60.87
A549_ATCC	63.00	62.40	75.75	62.95	48.29	45.89	52.92
ACHN	62.43	80.67	72.22	75.46	41.62	48.46	55.54
BT_549	74.12	75.01	74.17	63.26	55.45	52.86	60.78
CAKI_1	65.25	71.71	87.03	72.51	47.48	48.69	57.88
CCRF_CEM	75.20	66.22	67.08	67.45	52.89	48.39	54.30
COLO_205	75.99	73.30	77.83	77.17	54.21	55.52	58.14
DLD_1	64.51	71.62	63.82	68.08	60.88	48.37	63.90
DMS_114	53.48	51.33	68.83	58.21	45.78	40.64	57.82
DMS_273	59.87	54.18	51.37	60.48	40.39	38.84	67.65
DU_145	69.71	71.13	82.27	78.84	55.44	53.49	55.74
EKVX	65.54	67.97	77.02	67.65	48.24	47.76	51.24
HCC_2998	70.88	69.36	76.49	69.92	52.12	48.69	56.40
MDA_MB_435	74.95	79.39	82.76	77.73	50.75	56.04	58.03
NCI_ADR_RES	69.29	67.83	79.59	70.94	48.43	50.60	54.70
SNB_78	56.18	62.22	81.32	61.94	54.34	49.36	62.26
Average	67.42	68.49	74.73	68.69	50.81	49.26	58.52

Table 22: Robustness analysis to compare iREFINED-CNN-GM (integrated REFINED CNN with geometric mean) with 7 other competing models per Bias and each cell line of the NCI60 dataset. Each cell of the table represents the percentage for which iREFINED-CNN-GM outperforms the paired competing model.

Cell lines	MDS	Isomap	LLE	LE	iREFINED-CNN-AM	REFINED-CNN model stacking	REFINED-CNN image stacking
786_0	64.75	96.14	75.53	85.67	66.09	80.24	84.33
A498	63.70	63.62	56.40	67.82	67.41	78.16	77.75
A549_ATCC	65.07	69.04	75.65	47.50	55.85	69.31	49.45
ACHN	69.15	85.26	87.41	81.58	53.90	77.84	65.44
BT_549	79.11	92.38	79.10	64.33	68.07	83.33	81.99
CAKI_1	57.27	64.33	77.20	83.51	59.43	69.59	60.07
CCRF_CEM	86.52	78.65	61.89	83.83	65.09	70.14	66.51
COLO_205	62.90	64.11	67.23	83.70	78.56	80.44	68.16
DLD_1	57.49	83.50	76.64	60.77	60.49	54.91	82.82
DMS_114	34.43	31.30	53.51	36.67	28.20	26.16	24.35
DMS_273	51.05	36.32	28.90	27.73	36.85	32.34	65.98
DU_145	66.54	55.83	72.09	73.78	71.78	66.02	62.44
EKVX	65.73	86.41	67.89	61.54	59.28	70.65	62.63
HCC_2998	50.55	68.05	62.24	45.97	59.69	69.02	42.72
MDA_MB_435	73.95	86.62	80.55	76.97	70.03	86.71	77.40
NCI_ADR_RES	53.77	27.44	53.04	78.92	50.70	51.94	50.32
SNB_78	47.51	49.78	87.26	58.23	47.94	51.50	53.38
Average	61.73	66.99	68.38	65.80	58.79	65.78	63.28

Table 23: Robustness analysis to compare REFINED-CNN model stacking with 7 other competing models per NRMSE and each cell line of the NCI60 dataset. Each cell of the table represents the percentage for which REFINED-CNN model stacking outperforms the paired competing model.

Cell lines	MDS	Isomap	LLE	LE	iREFINED-CNN-AM	iREFINED-CNN-GM	REFINED-CNN image stacking
786_0	79.50	84.75	87.78	80.29	51.09	48.63	61.68
A498	74.88	78.73	75.21	65.44	55.97	54.72	65.88
A549_ATCC	68.38	68.30	80.38	77.88	54.61	57.07	60.43
ACHN	66.88	84.66	77.77	78.76	46.80	54.53	59.11
BT_549	81.55	77.40	82.23	67.25	56.44	56.59	57.93
CAKI_1	71.96	78.18	95.74	76.13	52.28	54.62	58.34
CCRF_CEM	83.14	72.54	70.97	80.10	55.59	55.70	59.01
COLO_205	79.80	72.97	78.61	75.30	48.83	48.05	54.35
DLD_1	67.42	82.29	66.86	72.77	63.25	53.11	67.81
DMS_114	67.78	62.29	77.86	77.09	61.89	64.24	74.60
DMS_273	73.80	68.56	67.64	78.87	56.49	67.28	79.04
DU_145	72.17	72.20	83.12	77.31	55.70	48.40	55.82
EKVX	74.11	79.77	84.91	73.68	51.51	56.49	57.46
HCC_2998	76.47	72.92	77.82	76.74	55.49	53.89	62.21
MDA_MB_435	78.17	78.22	88.81	84.49	44.39	48.11	52.26
NCI_ADR_RES	73.94	78.71	92.43	79.39	46.81	54.55	53.88
SNB_78	60.34	69.02	80.52	65.54	58.72	51.30	65.12
Average	73.55	75.38	80.51	75.71	53.87	54.55	61.47

Table 24: Robustness analysis to compare REFINED-CNN model stacking with 7 other competing models per NMAE and each cell line of the NCI60 dataset. Each cell of the table represents the percentage for which REFINED-CNN model stacking outperforms the paired competing model.

Cell lines	MDS	Isomap	LLE	LE	iREFINED-CNN-AM	iREFINED-CNN-GM	REFINED-CNN image stacking
7786_0	74.39	70.72	90.84	74.80	34.88	36.99	61.05
A498	72.49	80.39	74.26	61.68	50.66	40.42	54.69
A549_ATCC	61.86	64.58	85.77	79.10	41.28	38.91	53.22
ACHN	62.30	77.11	63.56	74.96	25.34	40.40	52.34
BT_549	70.29	69.01	91.14	60.75	58.43	40.98	50.54
CAKI_1	65.93	83.36	97.60	68.98	39.39	45.41	47.66
CCRF_CEM	69.90	64.28	65.09	60.82	53.41	37.60	46.24
COLO_205	78.76	65.19	76.31	70.37	38.17	29.05	47.19
DLD_1	65.36	80.69	61.10	68.72	45.51	42.03	63.84
DMS_114	60.24	59.06	91.40	77.89	47.55	62.53	83.09
DMS_273	88.00	78.04	58.80	85.84	36.41	49.97	84.44
DU_145	60.07	67.45	80.34	72.32	45.76	44.12	55.46
EKVX	72.22	72.24	83.05	66.67	41.69	47.40	52.19
HCC_2998	70.97	61.12	81.79	74.65	35.23	33.73	60.87
MDA_MB_435	78.35	70.72	93.28	90.44	34.84	34.84	43.00
NCI_ADR_RES	69.96	77.08	97.04	74.71	40.06	41.70	54.47
SNB_78	57.66	72.29	88.19	69.58	37.06	38.02	65.09
Average	69.34	71.37	81.15	72.49	41.51	41.42	57.38

Table 25: Robustness analysis to compare REFINED-CNN model stacking with 7 other competing models per PCC and each cell line of the NCI60 dataset. Each cell of the table represents the percentage for which REFINED-CNN model stacking outperforms the paired competing model.

Cell lines	MDS	Isomap	LLE	LE	iREIFNED-CNN-AM	iREFINED-CNN-GM	REFINED-CNN image stacking
786_0	74.24	70.68	85.35	74.03	49.89	48.49	62.31
A498	69.17	71.21	71.45	63.34	53.65	51.09	60.94
A549_ATCC	64.30	69.08	79.72	69.32	52.12	55.30	57.35
ACHN	66.25	83.43	75.30	77.07	43.54	51.28	56.11
BT_549	74.04	74.10	71.94	59.45	54.81	49.47	57.50
CAKI_1	68.07	74.65	89.53	74.31	49.47	52.45	57.13
CCRF_CEM	74.24	68.02	69.15	72.05	54.62	52.71	57.26
COLO_205	71.76	67.32	72.03	73.10	49.60	47.18	51.92
DLD_1	66.56	74.62	66.09	70.00	61.87	53.69	65.62
DMS_114	64.60	58.15	77.72	67.15	53.20	60.74	66.08
DMS_273	70.88	65.49	61.40	71.29	50.46	61.90	79.35
DU_145	68.26	69.87	79.35	74.42	53.32	48.03	53.94
EKVX	68.52	72.77	79.69	68.26	50.35	54.61	55.40
HCC_2998	73.48	70.79	76.75	71.59	53.96	51.04	57.52
MDA_MB_435	69.41	75.42	80.22	73.21	42.94	43.66	51.56
NCI_ADR_RES	69.52	70.06	81.70	73.00	45.06	50.71	53.35
SNB_78	55.71	64.73	82.05	62.45	53.75	51.67	63.60
Average	68.76	70.61	76.44	70.24	51.33	52.00	59.23

Table 26: Robustness analysis to compare REFINED-CNN model stacking with 7 other competing models per Bias and each cell line of the NCI60 dataset. Each cell of the table represents the percentage for which REFINED-CNN model stacking outperforms the paired competing model.

Cell lines	MDS	Isomap	LLE	LE	iREFINED-CNN-AM	iREFINED-CNN-GM	REFINED-CNN model stacking
786_0	32.11	70.03	45.72	62.82	31.75	20.29	58.96
A498	33.13	35.91	31.97	34.77	42.82	28.45	48.83
A549_ATCC	52.08	56.38	61.77	39.24	40.15	37.47	40.14
ACHN	44.00	63.36	66.60	56.02	27.54	22.36	32.57
BT_549	58.37	67.02	54.54	33.32	35.69	23.42	55.20
CAKI_1	34.50	42.20	58.22	69.06	36.96	32.49	38.84
CCRF_CEM	71.38	63.78	50.97	68.68	42.49	34.92	45.89
COLO_205	33.82	38.64	43.05	60.90	55.07	29.65	34.98
DLD_1	51.07	75.06	74.31	57.30	56.58	48.94	81.51
DMS_114	59.41	55.18	74.77	59.82	46.77	74.83	48.77
DMS_273	66.87	53.28	39.19	46.09	53.02	70.73	77.21
DU_145	55.84	41.08	64.30	59.47	57.24	40.66	52.35
EKVX	46.13	74.81	47.80	38.56	41.25	31.69	40.32
HCC_2998	37.91	52.40	48.46	28.53	42.29	37.09	27.67
MDA_MB_435	37.17	55.32	47.51	39.03	23.36	14.93	31.71
NCI_ADR_RES	56.77	25.45	51.75	81.01	49.84	49.66	51.86
SNB_78	46.70	48.35	89.46	57.18	47.59	54.83	56.61
Average	48.07	54.01	55.90	52.46	42.96	38.38	48.44

Table 27: Gap statistics analysis to compare REFINED-CNN model stacking, iREFINED-CNN GM/AM, and REFINED-CNN image stacking models with 4 other single REFINED-CNN models in terms of NRMSE per each cell line paired with the null model. The wider (larger) Gap value indicates better performance.

Cell lines	Isomap	MDS	LLE	LE	iREFINED -CNN-AM	iREFINED -CNN-GM	REFINED-CNN image stacking	REFINED-CNN model stacking
786_0	0.703	0.715	0.678	0.714	0.803	0.804	0.775	0.807
A498	0.700	0.708	0.703	0.742	0.774	0.774	0.744	0.783
A549_ATCC	0.758	0.761	0.723	0.730	0.805	0.803	0.795	0.822
ACHN	0.697	0.764	0.727	0.723	0.828	0.799	0.788	0.812
BT_549	0.675	0.650	0.650	0.703	0.735	0.740	0.729	0.751
CAKI_1	0.692	0.723	0.584	0.707	0.779	0.776	0.763	0.784
CCRF_CEM	0.714	0.677	0.717	0.692	0.757	0.762	0.754	0.774
COLO_205	0.737	0.715	0.717	0.734	0.812	0.818	0.801	0.811
DLD_1	0.695	0.750	0.749	0.731	0.766	0.799	0.755	0.807
DMS_114	0.754	0.733	0.699	0.698	0.751	0.753	0.707	0.790
DMS_273	0.742	0.726	0.744	0.698	0.779	0.748	0.702	0.799
DU_145	0.707	0.713	0.675	0.694	0.769	0.783	0.768	0.777
EKVX	0.664	0.686	0.637	0.678	0.755	0.740	0.743	0.761
HCC_2998	0.732	0.714	0.707	0.705	0.778	0.790	0.763	0.799
MDA_MB_435	0.684	0.688	0.633	0.652	0.779	0.766	0.759	0.764
NCI_ADR_RES	0.649	0.685	0.590	0.658	0.765	0.745	0.740	0.753
SNB_78	0.675	0.711	0.623	0.693	0.722	0.744	0.691	0.748
Average	0.705	0.713	0.680	0.703	0.774	0.773	0.752	0.785

Table 28: Gap statistics analysis to compare REFINED-CNN model stacking, iREFINED-CNN GM/AM, and REFINED-CNN image stacking models with 4 other single REFINED-CNN models in terms of NMAE per each cell line paired with the null model. The wider (larger) Gap value indicates better performance.

Cell lines	Isomap	MDS	LLE	LE	iREFINED -CNN-AM	iREFINED -CNN-GM	REFINED-CNN image stacking	REFINED-CNN model stacking
786_0	0.653	0.644	0.569	0.645	0.738	0.731	0.676	0.701
A498	0.598	0.626	0.620	0.658	0.686	0.709	0.674	0.683
A549_ATCC	0.690	0.698	0.621	0.644	0.751	0.750	0.720	0.726
ACHN	0.623	0.662	0.663	0.629	0.761	0.721	0.687	0.694
BT_549	0.605	0.597	0.513	0.621	0.629	0.671	0.640	0.644
CAKI_1	0.579	0.637	0.450	0.636	0.711	0.695	0.689	0.681
CCRF_CEM	0.638	0.621	0.625	0.638	0.659	0.699	0.677	0.667
COLO_205	0.672	0.625	0.640	0.664	0.737	0.760	0.713	0.707
DLD_1	0.612	0.668	0.676	0.644	0.718	0.725	0.669	0.704
DMS_114	0.664	0.656	0.549	0.603	0.693	0.657	0.573	0.685
DMS_273	0.606	0.582	0.680	0.574	0.740	0.704	0.595	0.702
DU_145	0.633	0.654	0.597	0.621	0.696	0.692	0.667	0.679
EKVX	0.602	0.593	0.556	0.611	0.675	0.662	0.650	0.654
HCC_2998	0.660	0.631	0.600	0.615	0.723	0.722	0.662	0.685
MDA_MB_435	0.615	0.585	0.502	0.529	0.704	0.699	0.676	0.663
NCI_ADR_RES	0.570	0.609	0.451	0.594	0.679	0.677	0.651	0.658
SNB_78	0.585	0.640	0.524	0.601	0.709	0.698	0.616	0.664
Average	0.624	0.631	0.579	0.619	0.706	0.704	0.661	0.682

Table 29: Gap statistics analysis to compare REFINED-CNN model stacking, iREFINED-CNN GM/AM, and REFINED-CNN image stacking models with 4 other single REFINED CNN models in terms of PCC per each cell line paired with the null model. The wider (larger) Gap value indicates better performance.

Cell lines	Isomap	MDS	LLE	LE	iREFINED -CNN-AM	iREFINED -CNN-GM	REFINED-CNN image stacking	REFINED-CNN model stacking
786_0	0.662	0.655	0.619	0.651	0.724	0.727	0.691	0.725
A498	0.643	0.645	0.643	0.667	0.692	0.701	0.672	0.700
A549_ATCC	0.685	0.690	0.649	0.681	0.728	0.725	0.717	0.736
ACHN	0.621	0.686	0.657	0.645	0.747	0.724	0.712	0.728
BT_549	0.594	0.589	0.599	0.638	0.660	0.675	0.645	0.669
CAKI_1	0.628	0.653	0.561	0.630	0.701	0.696	0.683	0.701
CCRF_CEM	0.635	0.616	0.638	0.627	0.678	0.685	0.673	0.690
COLO_205	0.677	0.664	0.656	0.658	0.729	0.738	0.721	0.726
DLD_1	0.636	0.669	0.667	0.652	0.683	0.715	0.673	0.721
DMS_114	0.679	0.661	0.611	0.652	0.693	0.675	0.651	0.705
DMS_273	0.672	0.650	0.680	0.646	0.709	0.681	0.613	0.715
DU_145	0.643	0.643	0.602	0.620	0.688	0.703	0.688	0.695
EKVX	0.596	0.614	0.572	0.611	0.675	0.667	0.662	0.676
HCC_2998	0.654	0.649	0.637	0.652	0.706	0.711	0.694	0.715
MDA_MB_435	0.609	0.620	0.588	0.610	0.700	0.694	0.677	0.680
NCI_ADR_RES	0.615	0.610	0.560	0.598	0.682	0.666	0.656	0.670
SNB_78	0.617	0.645	0.527	0.619	0.656	0.667	0.615	0.670
Average	0.639	0.645	0.616	0.639	0.697	0.697	0.673	0.701

Table 30: Gap statistics analysis to compare REFINED-CNN model stacking, iREFINED-CNN GM/AM, and REFINED-CNN image stacking models with 4 other single REFINED CNN models in terms of Bias per each cell line paired with the null model. The wider (larger) Gap value indicates better performance.

Cell lines	Isomap	MDS	LLE	LE	iREFINED -CNN-AM	iREFINED -CNN-GM	REFINED-CNN image stacking	REFINED-CNN model stacking
786_0	0.423	0.565	0.510	0.452	0.558	0.606	0.478	0.499
A498	0.532	0.538	0.547	0.524	0.516	0.570	0.483	0.480
A549_ATCC	0.541	0.551	0.508	0.611	0.577	0.594	0.584	0.551
ACHN	0.471	0.540	0.462	0.493	0.600	0.611	0.568	0.515
BT_549	0.412	0.455	0.457	0.530	0.508	0.575	0.460	0.466
CAKI_1	0.505	0.528	0.463	0.424	0.522	0.536	0.525	0.482
CCRF_CEM	0.443	0.412	0.480	0.418	0.494	0.524	0.486	0.473
COLO_205	0.582	0.581	0.565	0.507	0.537	0.617	0.576	0.534
DLD_1	0.426	0.513	0.433	0.502	0.495	0.538	0.410	0.526
DMS_114	0.506	0.493	0.426	0.484	0.528	0.430	0.536	0.512
DMS_273	0.514	0.456	0.568	0.543	0.514	0.460	0.412	0.520
DU_145	0.535	0.487	0.465	0.471	0.479	0.543	0.507	0.503
EKVX	0.400	0.488	0.472	0.502	0.514	0.540	0.496	0.471
HCC_2998	0.494	0.540	0.503	0.565	0.528	0.538	0.560	0.493
MDA_MB_435	0.437	0.496	0.461	0.499	0.531	0.574	0.497	0.454
NCI_ADR_RES	0.567	0.465	0.472	0.364	0.490	0.473	0.468	0.481
SNB_78	0.517	0.530	0.340	0.486	0.539	0.504	0.486	0.514
Average	0.489	0.508	0.478	0.493	0.525	0.543	0.502	0.498

Table 31: Robustness analysis to compare iREFINED-CNN AM (integrated REFINED with arithmetic mean) with 7 other competing models per NRMSE and each cell line of the NCI-ALMANAC dataset. Each cell of the table represents the percentage for which iREFINED-CNN AM outperforms the paired competing model.

Cell lines	MDS	Isomap	LLE	LE	REFINED-CNN model stacking	iREFINED-CNN GM	REFINED-CNN image stacking
DU-145	33.32	80.75	58.39	38.59	12.65	61.38	92.89
CCRF-CEM	52.89	68.97	52.04	65.53	24.08	44.25	80.53
HCC-2998	13.77	7.06	85.71	8.51	0.36	7.85	100
HOP-62	92.86	53.26	62.88	32.75	15.24	35.26	94.32
HCT-116	100	95.26	96.48	27.63	3.96	49.98	100
KM12	14.19	93.5	91	75.49	4.89	30.92	98.96
786-0	100	100	100	100	2.43	53.02	100
K-562	52.73	93.39	53.98	60.37	9.43	65.71	93.47
EKVX	59.28	54.22	64.08	59.77	8.97	42.47	100
ACHN	76.67	56.88	66.67	79.5	39.18	58.68	74.82
HL-60(TB)	71.51	93.35	76.41	78.88	16.04	71.68	94.41
HCT-15	73.71	41.59	78.39	91.25	19.65	99.1	98.62
HT29	58.35	64.9	77.35	59.76	7.88	92.06	100
A498	8.24	39.46	6.53	30.26	2.29	17.22	97.41
IGROV1	62.25	42.53	52.73	56.19	44.98	58.49	94.18
A549_ATCC	68.12	48.11	81.27	58.49	29.16	53.37	72.06
COLO 205	49.18	34.55	92.54	49.87	10.07	23.57	96.32
LOX IMVI	25.82	46.49	42.09	52.44	22.17	21.12	100
HOP-92	3.82	0.47	4.88	3.12	0.22	5.53	100
MALME-3M	61.08	57.35	59.25	74.42	16.49	59.54	95.62
Average	53.89	58.60	65.13	55.14	14.51	47.56	94.18

Table 32: Robustness analysis to compare iREFINED-CNN AM (integrated REFINED with arithmetic mean) with 7 other competing models per NMAE and each cell line of the NCI-ALMANAC dataset. Each cell of the table represents the percentage for which iREFINED-CNN AM outperforms the paired competing model.

Cell lines	MDS	Isomap	LLE	LE	REFINED-CNN model stacking	iREFINED-CNN GM	REFINED-CNN image stacking
DU-145	27.76	82.90	66.43	37.39	4.80	53.01	96.04
CCRF-CEM	48.09	72.04	46.09	74.86	15.22	47.64	85.84
HCC-2998	9.60	5.44	92.23	4.42	1.03	5.43	98.97
HOP-62	98.34	45.06	67.42	27.33	5.87	25.66	99.96
HCT-116	100.00	97.49	98.09	11.77	0.19	35.45	98.77
KM12	9.22	97.81	91.72	73.29	0.29	32.57	99.77
786-0	100.00	99.92	100.00	100.00	1.34	71.41	100.00
K-562	51.68	98.80	59.95	68.32	3.84	69.72	100.00
EKVX	59.19	54.24	69.89	59.49	1.18	41.50	100.00
ACHN	87.71	65.48	82.85	91.32	37.10	61.77	84.50
HL-60(TB)	79.25	92.96	90.19	89.23	8.19	79.36	98.95
HCT-15	69.05	36.33	80.18	92.24	7.42	98.45	100.00
HT29	52.49	64.44	81.68	46.16	0.11	96.15	100.00
A498	2.93	34.20	6.74	7.59	1.90	6.89	97.38
IGROV1	63.00	44.82	54.97	66.88	43.40	64.59	98.84
A549_ATCC	76.93	50.91	91.73	66.41	22.23	62.87	74.81
COLO 205	35.62	27.21	97.88	48.90	5.00	15.32	100.00
LOX IMVI	11.62	33.62	20.65	40.79	2.54	8.46	100.00
HOP-92	1.55	0.54	1.98	1.03	0.88	1.63	100.00
MALME-3M	65.13	63.49	55.98	81.09	3.50	61.50	100.00
Average	52.46	58.39	67.83	54.43	8.30	46.97	96.69

Table 33: Robustness analysis to compare iREFINED-CNN AM (integrated REFINED with arithmetic mean) with 7 other competing models per PCC and each cell line of the NCI-ALMANAC dataset. Each cell of the table represents the percentage for which iREFINED-CNN AM outperforms the paired competing model.

Cell lines	MDS	Isomap	LLE	LE	REFINED-CNN model stacking	iREFINED-CNN GM	REFINED-CNN image stacking
DU-145	44.44	56.51	54.92	44.76	16.97	48.63	85.66
CCRF-CEM	41.00	62.99	48.49	52.79	22.79	46.71	79.01
HCC-2998	13.86	10.87	36.13	10.48	2.28	10.65	97.41
HOP-62	55.27	54.44	54.58	38.27	18.11	36.22	81.60
HCT-116	85.07	43.96	72.14	34.62	2.53	54.86	97.01
KM12	13.33	11.68	55.16	15.47	3.34	9.89	96.27
786-0	100.00	93.07	86.44	84.41	1.91	39.77	98.07
K-562	49.52	40.36	52.37	47.67	1.55	59.40	87.61
EKVX	58.71	50.91	64.69	60.17	2.59	37.33	94.03
ACHN	59.82	51.44	62.97	72.60	41.43	53.83	69.53
HL-60(TB)	59.02	64.21	60.41	78.34	21.52	51.18	95.11
HCT-15	71.05	39.51	47.77	66.00	16.89	69.46	95.26
HT29	50.31	49.74	63.45	57.95	0.32	58.64	98.99
A498	31.89	37.95	36.04	54.74	20.14	39.28	96.25
IGROV1	58.97	38.97	48.77	48.66	35.00	56.55	81.93
A549_ATCC	59.48	46.32	51.22	51.12	30.58	37.17	67.81
COLO 205	43.48	33.66	49.10	36.95	12.39	24.67	84.34
LOX IMVI	59.06	73.14	67.60	69.22	43.05	50.87	95.70
HOP-92	3.94	5.63	12.96	13.23	0.73	0.38	96.58
MALME-3M	56.76	49.12	59.21	63.26	11.57	57.57	90.96
Average	50.75	45.72	54.22	50.04	15.28	42.15	89.46

Table 34: Robustness analysis to compare iREFINED-CNN AM (integrated REFINED with arithmetic mean) with 7 other competing models per Bias and each cell line of the NCI-ALMANAC dataset. Each cell of the table represents the percentage for which iREFINED-CNN AM outperforms the paired competing model.

Cell lines	MDS	Isomap	LLE	LE	REFINED-CNN model stacking	iREFINED-CNN GM	REFINED-CNN image stacking
DU-145	28.45	52.78	95.81	71.39	12.86	23.34	78.67
CCRF-CEM	44.52	98.05	48.29	52.95	34.29	17.41	88.48
HCC-2998	89.4	70.56	100	74.12	45	91.07	99.99
HOP-62	2.15	31.5	66.41	21.13	1.14	15.24	99.62
HCT-116	1.84	78.24	29.6	8.27	0.07	16.05	100
KM12	11.01	4.6	99.36	3.02	0	1.86	100
786-0	100	97.42	94.3	71.94	0.28	60.95	100
K-562	94.26	41.65	91.23	90.95	42.88	77.65	96.96
EKVX	80.48	56.07	72.24	86.35	19.38	64.35	99.93
ACHN	96.56	57.64	84.62	88.07	19.49	35.27	91.61
HL-60(TB)	82.74	57.91	42.44	92.51	10	24.53	98.68
HCT-15	38.75	10.74	23.87	56.36	5.8	2.08	100
HT29	34.27	6.69	67.72	21.52	1.28	38.04	99.86
A498	16.87	28.55	54.42	8.95	2.8	57.17	99.76
IGROV1	66.58	76.28	67.29	71.39	20.12	37.29	100
A549_ATCC	66.02	56.98	99.34	83.13	14.99	39.64	94.06
COLO 205	48.02	7.34	0.37	2.69	3.3	12.6	100
LOX IMVI	1.44	0.14	15.47	22.24	1.9	3.3	100
HOP-92	71.85	5.72	99.99	54.4	16.43	33.12	99.99
MALME-3M	82.39	24.96	31.38	6.5	8.28	44.93	98.32
Average	52.88	43.19	64.21	49.39	13.01	34.79	97.30

Table 35: Robustness analysis to compare iREFINED-CNN GM (integrated REFINED with geometric mean) with 7 other competing models per NRMSE and each cell line of the NCI-ALMANAC dataset. Each cell of the table represents the percentage for which iREFINED-CNN GM outperforms the paired competing model.

Cell lines	MDS	Isomap	LLE	LE	iREFINED-CNN AM	REFINED-CNN model stacking	REFINED-CNN image stacking
DU-145	24.85	70.66	44.81	32.90	38.78	4.78	52.28
CCRF-CEM	56.67	74.57	56.08	65.43	60.06	25.82	56.93
HCC-2998	62.93	41.53	100.00	49.11	94.76	4.88	100.00
HOP-62	100.00	67.72	75.48	51.00	67.74	31.04	79.70
HCT-116	100.00	95.43	96.63	35.21	58.16	5.50	100.00
KM12	32.45	98.53	97.97	86.66	72.05	6.26	100.00
786-0	97.48	100.00	100.00	95.14	48.62	1.24	100.00
K-562	36.61	86.73	46.28	49.33	38.77	3.91	36.89
EKVX	69.49	66.24	72.42	67.92	61.92	9.35	72.87
ACHN	73.77	55.28	65.18	79.67	49.06	30.25	62.03
HL-60(TB)	57.21	85.87	59.71	70.20	35.25	13.39	64.89
HCT-15	16.77	6.63	19.34	28.02	2.82	2.81	36.95
HT29	18.06	25.90	34.09	26.42	15.36	1.77	57.71
A498	38.71	75.08	48.29	63.97	80.63	24.12	85.18
IGROV1	57.92	34.92	45.74	45.62	45.73	35.16	95.20
A549_ATCC	71.98	49.41	77.92	62.79	45.74	27.93	56.85
COLO 205	78.60	66.32	100.00	80.55	78.67	28.50	95.71
LOX IMVI	58.29	83.35	79.54	88.69	83.47	47.21	100.00
HOP-92	53.98	57.36	98.55	67.98	100.00	24.71	93.23
MALME-3M	48.92	51.35	49.59	67.99	46.42	7.11	54.76
Average	57.73	64.64	68.38	60.73	56.20	16.79	75.06

Table 36: Robustness analysis to compare iREFINED-CNN GM (integrated REFINED with geometric mean) with 7 other competing models per NMAE and each cell line of the NCI-ALMANAC dataset. Each cell of the table represents the percentage for which iREFINED-CNN GM outperforms the paired competing model.

Cell lines	MDS	Isomap	LLE	LE	iREFINED-CNN AM	REFINED-CNN model stacking	REFINED-CNN image stacking
DU-145	24.90	79.28	71.96	35.62	50.23	8.26	51.18
CCRF-CEM	51.13	75.96	53.78	84.23	57.36	17.20	57.20
HCC-2998	62.67	39.00	99.73	46.97	95.46	1.32	100.00
HOP-62	99.00	67.94	86.95	55.06	76.86	23.07	85.11
HCT-116	99.15	100.00	100.00	22.35	65.40	2.14	100.00
KM12	21.34	100.00	97.96	85.58	67.24	1.23	100.00
786-0	100.00	100.00	100.00	98.60	30.94	0.17	100.00
K-562	29.24	100.00	40.15	51.47	35.06	0.95	33.03
EKVX	72.95	61.97	77.17	69.48	60.65	3.73	75.45
ACHN	78.95	50.00	74.32	84.82	35.63	23.50	63.92
HL-60(TB)	48.48	78.53	69.87	66.14	23.89	5.51	61.17
HCT-15	6.56	2.94	15.29	21.36	0.85	5.02	25.59
HT29	6.19	8.45	24.18	10.00	1.36	4.08	46.20
A498	19.38	84.18	38.81	51.47	94.94	13.96	82.69
IGROV1	51.01	31.32	40.37	48.02	33.85	30.14	95.53
A549_ATCC	63.40	39.15	82.07	50.94	37.00	12.61	27.73
COLO 205	77.36	71.49	99.04	86.50	86.92	24.53	100.00
LOX IMVI	56.16	81.66	71.64	89.94	90.50	29.83	100.00
HOP-92	62.98	55.14	96.63	70.97	100.00	9.07	100.00
MALME-3M	56.92	55.77	46.53	69.38	37.59	3.35	48.00
Average	54.39	64.14	69.32	59.94	54.09	10.98	72.64

Table 37: Robustness analysis to compare iREFINED-CNN GM (integrated REFINED with geometric mean) with 7 other competing models per PCC and each cell line of the NCI-ALMANAC dataset. Each cell of the table represents the percentage for which iREFINED-CNN GM outperforms the paired competing model.

Cell lines	MDS	Isomap	LLE	LE	iREFINED-CNN AM	REFINED-CNN model stacking	REFINED-CNN image stacking
DU-145	45.47	60.88	56.22	46.82	51.58	21.51	59.65
CCRF-CEM	56.38	68.92	58.10	63.93	59.97	30.65	61.86
HCC-2998	69.08	54.83	88.63	58.18	94.34	8.25	89.66
HOP-62	70.60	71.47	67.32	53.80	63.50	33.52	72.16
HCT-116	88.31	36.55	72.27	29.45	43.50	3.00	100.00
KM12	60.76	56.87	93.59	62.80	89.58	2.80	100.00
786-0	99.66	100.00	95.62	90.44	59.68	5.60	100.00
K-562	41.95	33.37	44.49	41.44	41.20	5.74	45.02
EKVX	65.72	65.33	70.45	63.55	59.35	7.71	66.92
ACHN	59.59	49.70	59.08	69.68	47.80	35.03	57.13
HL-60(TB)	60.18	64.56	62.12	74.75	47.28	19.95	73.93
HCT-15	52.90	23.61	31.25	46.72	29.97	11.09	59.32
HT29	46.54	47.59	58.27	48.07	41.61	3.97	55.22
A498	44.84	49.11	51.11	67.41	63.87	34.11	91.61
IGROV1	56.99	37.02	44.88	49.19	44.29	39.83	73.31
A549_ATCC	71.76	61.14	67.48	65.07	61.99	41.62	60.16
COLO 205	72.77	60.12	77.62	66.56	78.85	30.40	73.92
LOX IMVI	58.11	73.72	68.31	76.57	50.11	44.29	100.00
HOP-92	69.56	65.82	86.74	83.27	97.74	36.56	95.51
MALME-3M	50.42	40.78	52.66	56.60	42.84	13.02	51.29
Average	62.08	56.07	65.31	60.71	58.45	21.43	74.33

Table 38: Robustness analysis to compare iREFINED-CNN GM (integrated REFINED with geometric mean) with 7 other competing models per Bias and each cell line of the NCI-ALMANAC dataset. Each cell of the table represents the percentage for which iREFINED-CNN GM outperforms the paired competing model.

Cell lines	MDS	Isomap	LLE	LE	iREFINED-CNN AM	REFINED-CNN model stacking	REFINED-CNN image stacking
DU-145	57.12	77.52	98.8	90.03	76.66	38.49	66.25
CCRF-CEM	79.05	99.87	81.57	83.16	82.59	71.21	95.83
HCC-2998	48.53	19.57	99.96	21.68	8.93	3.62	96.61
HOP-62	13.32	68.24	91.8	57.31	84.76	10.78	92.71
HCT-116	9.96	95.73	63.53	35.57	83.95	1.41	100
KM12	76.42	60.95	100	48.33	98.14	0.04	100
786-0	100	95.97	91.65	63.49	39.05	0.1	100
K-562	78.73	15.92	72.44	71.59	22.35	12.62	67.01
EKVX	70.64	41.56	59.76	79.35	35.65	8.66	91.53
ACHN	98.52	71.1	91.98	93.47	64.73	31.94	81.21
HL-60(TB)	95.1	80.78	69.89	98.56	75.47	29.09	95.01
HCT-15	95.14	80.29	90.92	98.15	97.92	73.57	100
HT29	45.96	11.57	77.88	32.29	61.96	3	87.13
A498	13.14	22.37	46.86	7.09	42.83	2.34	80.32
IGROV1	76.57	83.86	77.63	81.05	62.71	31.49	99.94
A549_ATCC	74.92	67.27	99.68	88.5	60.36	21.67	79.75
COLO 205	86.4	37.3	4.37	18.73	87.4	24.24	99.95
LOX IMVI	37.93	15	76.99	84.27	96.7	39.54	100
HOP-92	88.51	9.06	100	72.26	66.88	26.71	99.96
MALME-3M	83.29	29.68	35.84	7.95	55.07	11.72	78.21
Average	66.46	54.18	76.58	61.64	65.21	22.11	90.57

Table 39: Robustness analysis to compare REFINED-CNN model stacking with 7 other competing models per NRMSE and each cell line of the NCI-ALMANAC dataset. Each cell of the table represents the percentage for which stacked CNNs outperforms the paired competing model.

Cell lines	MDS	Isomap	LLE	LE	iREFINED-CNN AM	iREFINED-CNN GM	REFINED-CNN image stacking
DU-145	79.4	98.25	93.61	85.52	90.21	92.83	93.35
CCRF-CEM	77.48	89.96	79.44	88.18	79.31	72.91	80.93
HCC-2998	98.53	95.77	100	96.93	99.93	97.33	100
HOP-62	99.18	87.3	92.76	76.85	88.6	75.49	93.83
HCT-116	100	100	100	98.26	99.78	99.54	100
KM12	99.99	100	100	100	100	100	100
786-0	100	100	100	100	98.94	99.01	100
K-562	94.73	99.96	96.19	96.81	93.7	97.38	94.3
EKVX	96.98	96.42	98.05	97.07	94.62	92.74	98.48
ACHN	82.65	65.28	76.71	86.11	61.66	66.12	71.92
HL-60(TB)	91.97	98.18	95.03	96.06	81.6	91.13	94.8
HCT-15	93.71	78.46	96.01	98.04	85.13	99.38	98.9
HT29	97.58	97.74	99.43	96.14	96.51	99.86	99.89
A498	66.05	90.92	75.32	82.67	95.04	77.25	94.75
IGROV1	65.69	51.65	56.41	59.39	56.69	62.22	90.64
A549_ATCC	85.37	73.38	93	80.93	73.2	74	73.44
COLO 205	89.99	80.07	99.34	90.1	91.71	72.37	98.07
LOX IMVI	63.17	80.59	77.73	84.81	84.6	57.94	99.99
HOP-92	86.99	82.15	99.62	91.41	100	82.1	99.78
MALME-3M	94.24	90.91	90.29	96.58	89.01	92.05	94.08
Average	88.19	87.85	90.95	90.09	88.01	85.08	93.86

Table 40: Robustness analysis to compare REFINED-CNN model stacking with 7 other competing models per NMAE and each cell line of the NCI-ALMANAC dataset. Each cell of the table represents the percentage for which stacked CNNs outperforms the paired competing model.

Cell lines	MDS	Isomap	LLE	LE	iREFINED-CNN AM	iREFINED-CNN GM	REFINED-CNN image stacking
DU-145	87.28	99.62	98.76	93.02	95.98	96.27	95.78
CCRF-CEM	84.3	96.23	83.15	96.07	84.76	82.73	86.2
HCC-2998	99.87	99.03	100	99.65	100	99.43	100
HOP-62	99.98	92.25	97.9	83.46	94.27	81.38	97.38
HCT-116	100	100	100	99.54	99.98	99.96	100
KM12	100	100	100	100	100	100	100
786-0	100	100	100	100	99.76	99.92	100
K-562	98.51	100	99.03	99.52	98.09	99.64	98.6
EKVX	99.64	99.29	99.85	99.65	99.24	98.6	99.75
ACHN	94.07	79.33	91.35	95.76	66.64	76.83	85.25
HL-60(TB)	98.8	99.75	99.66	99.57	92.44	98.62	99.33
HCT-15	97.15	86.34	98.98	99.81	93.31	99.97	99.92
HT29	99.47	99.55	99.98	99.04	99.29	100	99.99
A498	68.92	98.49	84.04	88.23	99.49	89.22	97.84
IGROV1	66.19	50.1	59.87	68.14	55.62	67.9	97.83
A549_ATCC	94.11	81.7	98.67	91.05	79.51	88.2	73.64
COLO 205	92.77	87.16	100	96.16	96.97	78.11	99.88
LOX IMVI	75.77	92.93	87.25	95.95	97.34	70.95	100
HOP-92	96.04	90.24	99.99	97	100	90.89	100
MALME-3M	99.01	98.47	97.48	99.68	96.59	98.05	98.72
Average	92.59	92.52	94.80	95.07	92.46	90.83	96.51

Table 41: Robustness analysis to compare REFINED-CNN model stacking with 7 other competing models per PCC and each cell line of the NCI-ALMANAC dataset. Each cell of the table represents the percentage for which stacked CNNs outperforms the paired competing model.

Cell lines	MDS	Isomap	LLE	LE	iREFINED-CNN AM	iREFINED-CNN GM	REFINED-CNN image stacking
DU-145	79.26	87.07	86.28	80.86	82.75	81.76	87.45
CCRF-CEM	75.87	85.42	76.94	81.23	78.79	72.59	79.13
HCC-2998	97.41	95.46	99.42	96.23	99.73	94.87	99.62
HOP-62	83.91	84.18	83.07	73.8	80	70.11	86.28
HCT-116	99.98	98.34	99.92	96.82	98.87	99.47	100
KM12	100	100	100	99.99	100	99.98	100
786-0	100	100	99.94	99.89	97.37	96.17	100
K-562	93.04	89.67	93.81	91.53	92.48	95.31	93.12
EKVX	96.41	96.23	97.64	96.05	94.59	92.1	96.65
ACHN	71.21	63.09	71.16	79.46	59.94	65.71	68.83
HL-60(TB)	85.84	87.83	87.54	94.09	79.36	82.54	92.87
HCT-15	93.14	78.11	83.89	90.99	83.22	92.36	94.92
HT29	96.29	95.36	98.37	95.7	95.3	97.5	97.69
A498	60.18	66.05	68.41	81.89	76.27	68.16	96
IGROV1	66.89	48.85	56.56	59.03	58.46	62.94	80.84
A549_ATCC	78.67	69.14	74.99	71.63	70.49	60.38	66.29
COLO 205	85.87	78.14	90.39	81.18	88.39	71.08	86.43
LOX IMVI	64.05	75.84	71.42	75.77	55.55	58.07	99.59
HOP-92	81.74	75.78	92.71	90.2	99.07	64.48	98.11
MALME-3M	88.46	82.95	89.98	92.95	84.57	88.8	90.58
Average	84.911	82.8755	86.122	86.4645	83.76	80.719	90.72

Table 42: Robustness analysis to compare REFINED-CNN model stacking with 7 other competing models per Bias and each cell line of the NCI-ALMANAC dataset. Each cell of the table represents the percentage for which stacked CNNs outperforms the paired competing model.

Cell lines	MDS	Isomap	LLE	LE	iREFINED-CNN AM	iREFINED-CNN GM	REFINED-CNN image stacking
DU-145	69.47	88.04	99.84	96.13	87.14	61.51	78.67
CCRF-CEM	60.82	99.39	64.2	67.81	65.71	28.79	88.48
HCC-2998	94.97	78.81	100	82.65	55	96.38	99.99
HOP-62	51.83	95	99.43	91.78	98.86	89.22	99.62
HCT-116	64.91	100	99.17	96.77	99.93	98.59	100
KM12	100	99.98	100	99.9	100	99.96	100
786-0	100	100	100	99.9	99.72	99.9	100
K-562	98.95	47.6	97.89	97.43	57.12	87.38	96.96
EKVX	97.79	86.24	94.84	99.24	80.62	91.34	99.93
ACHN	99.57	84.4	96.89	97.69	80.51	68.06	91.61
HL-60(TB)	98.74	92.39	86.29	99.65	90	70.91	98.68
HCT-15	87.7	60.95	79.55	94.84	94.2	26.43	100
HT29	95.98	71.33	99.56	90.19	98.72	97	99.86
A498	81.87	91.42	97.82	62.92	97.2	97.66	99.76
IGROV1	90.08	95.22	92.07	93.65	79.88	68.51	100
A549_ATCC	91.88	89.13	99.98	97.25	85.01	78.33	94.06
COLO 205	96.41	64.83	13.36	40.62	96.7	75.76	100
LOX IMVI	48.28	21.27	84.21	89.99	98.1	60.46	100
HOP-92	96.57	23.12	100	87.86	83.57	73.29	99.99
MALME-3M	99.2	72.13	77.74	31.83	91.72	88.28	98.32
Average	86.25	78.06	89.14	85.91	86.99	77.89	97.30

Table 43: Gap statistics analysis to compare stacked CNNs, iREFINED-CNN GM/AM, and stacked images models with 4 other single REFINED CNN models in terms of NRMSE per each cell line paired with the null model. The wider (larger) Gap value indicates better performance.

Cell lines	Isomap	MDS	LLE	LE	iREFINED -CNN AM	iREFINED -CNN GM	REFINED-CNN image stacking	REFINED-CNN model stacking
786-0	0.648	0.598	0.526	0.697	0.887	0.887	0.659	0.945
A498	0.911	0.809	0.892	0.858	0.781	0.877	0.793	1.047
A549_ATCC	0.978	1.009	0.959	0.992	1.006	1.002	1.003	0.834
ACHN	0.983	1.019	0.995	0.965	1.025	1.018	1.005	1.027
CCRF-CEM	0.995	0.961	0.989	0.965	0.993	1.006	0.989	1.046
COLO 205	0.967	0.989	0.878	0.964	0.959	1.008	0.922	1.022
DU-145	0.993	0.904	0.951	0.980	0.962	0.944	0.941	1.018
EKVX	0.911	0.919	0.899	0.911	0.925	0.941	0.897	0.947
HCC-2998	0.893	0.930	0.697	0.922	0.776	0.914	0.693	0.946
HCT-116	0.195	0.713	0.704	0.895	0.847	0.853	0.276	0.935
HCT-15	0.938	0.991	0.927	0.907	0.978	0.861	0.893	1.008
HL-60(TB)	0.952	0.887	0.942	0.933	0.981	0.957	0.934	1.011
HOP-62	0.883	0.972	0.956	1.001	0.970	1.001	0.949	1.034
HOP-92	0.977	0.973	0.884	0.950	0.730	0.982	0.865	1.021
HT29	0.903	0.884	0.863	0.901	0.913	0.830	0.821	0.926
IGROV1	0.913	0.946	0.933	0.930	0.935	0.920	0.840	0.978
K-562	0.932	0.812	0.921	0.905	0.929	0.899	0.933	0.972
KM12	0.869	0.654	0.843	0.785	0.731	0.696	0.961	0.625
LOX IMVI	0.403	0.371	0.397	0.413	0.413	0.363	0.595	0.335
MALME-3M	0.448	0.457	0.442	0.477	0.44	0.459	0.455	0.405
Average	0.835	0.840	0.830	0.868	0.859	0.871	0.821	0.904

Table 44: Gap statistics analysis to compare stacked CNNs, iREFINED-CNN GM/AM, and stacked images models with 4 other single REFINED CNN models in terms of NMAE per each cell line paired with the null model. The wider (larger) Gap value indicates better performance.

Cell lines	Isomap	MDS	LLE	LE	iREFINED -CNN AM	iREFINED -CNN GM	REFINED-CNN image stacking	REFINED-CNN model stacking
786-0	0.659	0.573	0.527	0.738	0.950	0.916	0.656	1.002
A498	0.970	0.828	0.938	0.916	0.790	0.915	0.843	1.099
A549_ATCC	1.019	1.056	0.996	1.037	1.058	1.038	1.070	0.897
ACHN	1.030	1.066	1.039	1.016	1.085	1.068	1.054	1.087
CCRF-CEM	1.046	1.012	1.050	1.009	1.045	1.051	1.044	1.105
COLO 205	1.026	1.040	0.899	1.008	1.005	1.061	0.956	1.080
DU-145	1.043	0.961	0.986	1.028	1.009	1.007	1.008	1.072
EKVX	0.951	0.963	0.932	0.953	0.966	0.980	0.947	0.991
HCC-2998	0.927	0.971	0.691	0.957	0.817	0.952	0.711	0.992
HCT-116	0.131	0.740	0.745	0.958	0.885	0.910	0.259	1.001
HCT-15	1.003	1.049	0.978	0.950	1.031	0.895	0.936	1.070
HL-60(TB)	0.993	0.948	0.963	0.969	1.033	0.989	0.975	1.056
HOP-62	0.909	1.027	0.998	1.052	1.020	1.055	1.000	1.093
HOP-92	1.010	1.026	0.913	0.998	0.726	1.027	0.895	1.070
HT29	0.966	0.949	0.913	0.973	0.971	0.862	0.872	1.007
IGROV1	0.975	1.003	0.986	0.973	0.995	0.970	0.868	1.044
K-562	0.985	0.795	0.972	0.952	0.982	0.950	0.980	1.030
KM12	0.89	0.612	0.848	0.774	0.721	0.683	0.979	0.578
LOX IMVI	0.373	0.331	0.353	0.382	0.394	0.325	0.552	0.296
MALME-3M	0.421	0.422	0.406	0.453	0.399	0.418	0.412	0.36
Average	0.866	0.869	0.857	0.905	0.894	0.904	0.851	0.946

Table 45: Gap statistics analysis to compare stacked CNNs, iREFINED-CNN GM/AM, and stacked images models with 4 other single REFINED CNN models in terms of PCC per each cell line paired with the null model. The wider (larger) Gap value indicates better performance.

Cell lines	Isomap	MDS	LLE	LE	iREFINED -CNN AM	iREFINED -CNN GM	REFINED-CNN image stacking	REFINED-CNN model stacking
786-0	0.725	0.777	0.802	0.817	0.869	0.877	0.754	0.893
A498	0.882	0.872	0.874	0.851	0.858	0.871	0.797	0.937
A549_ATCC	0.915	0.924	0.920	0.922	0.922	0.929	0.924	0.834
ACHN	0.919	0.926	0.919	0.907	0.928	0.924	0.921	0.929
CCRF-CEM	0.917	0.905	0.916	0.911	0.915	0.921	0.914	0.935
COLO 205	0.908	0.915	0.900	0.913	0.904	0.922	0.905	0.928
DU-145	0.914	0.905	0.907	0.913	0.912	0.910	0.903	0.925
EKVX	0.874	0.878	0.868	0.876	0.881	0.890	0.873	0.892
HCC-2998	0.874	0.885	0.849	0.883	0.834	0.888	0.845	0.893
HCT-116	0.782	0.860	0.815	0.872	0.855	0.842	0.695	0.886
HCT-15	0.890	0.913	0.907	0.896	0.909	0.891	0.884	0.921
HL-60(TB)	0.903	0.898	0.904	0.891	0.911	0.910	0.890	0.922
HOP-62	0.908	0.908	0.912	0.921	0.914	0.922	0.907	0.931
HOP-92	0.913	0.915	0.893	0.896	0.852	0.926	0.866	0.926
HT29	0.874	0.872	0.859	0.869	0.876	0.867	0.862	0.882
IGROV1	0.875	0.896	0.887	0.886	0.887	0.880	0.856	0.908
K-562	0.888	0.898	0.884	0.888	0.887	0.875	0.886	0.904
KM12	0.81	0.805	0.726	0.801	0.737	0.82	0.517	0.83
LOXIMVI	0.969	0.977	0.972	0.968	0.982	0.981	0.893	0.99
MALME-3M	0.956	0.947	0.945	0.94	0.953	0.944	0.945	0.962
Average	0.885	0.894	0.883	0.891	0.889	0.899	0.852	0.911

Table 46: Gap statistics analysis to compare stacked CNNs, iREFINED-CNN GM/AM, and stacked images models with 4 other single REFINED CNN models in terms of Bias per each cell line paired with the null model. The wider (larger) Gap value indicates better performance.

Cell lines	Isomap	MDS	LLE	LE	iREFINED -CNN AM	iREFINED -CNN GM	REFINED-CNN image stacking	REFINED-CNN model stacking
786-0	0.666	0.438	0.421	0.353	0.311	0.329	0.677	0.157
A498	0.222	0.249	0.296	0.184	0.287	0.299	0.361	0.156
A549_ATCC	0.229	0.216	0.331	0.261	0.209	0.196	0.240	0.352
ACHN	0.282	0.191	0.239	0.247	0.181	0.160	0.207	0.095
CCRF-CEM	0.148	0.273	0.154	0.159	0.155	0.101	0.200	0.132
COLO 205	0.254	0.175	0.087	0.141	0.258	0.192	0.364	0.170
DU-145	0.164	0.205	0.303	0.234	0.200	0.152	0.181	0.174
EKVX	0.274	0.210	0.248	0.294	0.199	0.228	0.341	0.176
HCC-2998	0.242	0.186	0.466	0.193	0.142	0.246	0.367	0.162
HCT-116	0.163	0.385	0.300	0.250	0.336	0.272	0.534	0.196
HCT-15	0.206	0.150	0.181	0.232	0.223	0.090	0.379	0.124
HL-60(TB)	0.264	0.220	0.195	0.293	0.208	0.164	0.263	0.121
HOP-62	0.136	0.231	0.281	0.213	0.259	0.203	0.285	0.151
HOP-92	0.251	0.112	0.422	0.222	0.215	0.188	0.385	0.119
HT29	0.241	0.167	0.297	0.215	0.267	0.246	0.318	0.194
IGROV1	0.256	0.272	0.254	0.260	0.221	0.197	0.433	0.168
K-562	0.285	0.131	0.272	0.268	0.149	0.221	0.256	0.167
KM12	0.605	0.572	0.326	0.63	0.483	0.628	0.13	0.638
LOX IMVI	0.89	0.847	0.79	0.777	0.738	0.83	0.558	0.89
MALME-3M	0.806	0.703	0.796	0.876	0.76	0.77	0.713	0.851
Average	0.329	0.297	0.333	0.315	0.290	0.286	0.360	0.260

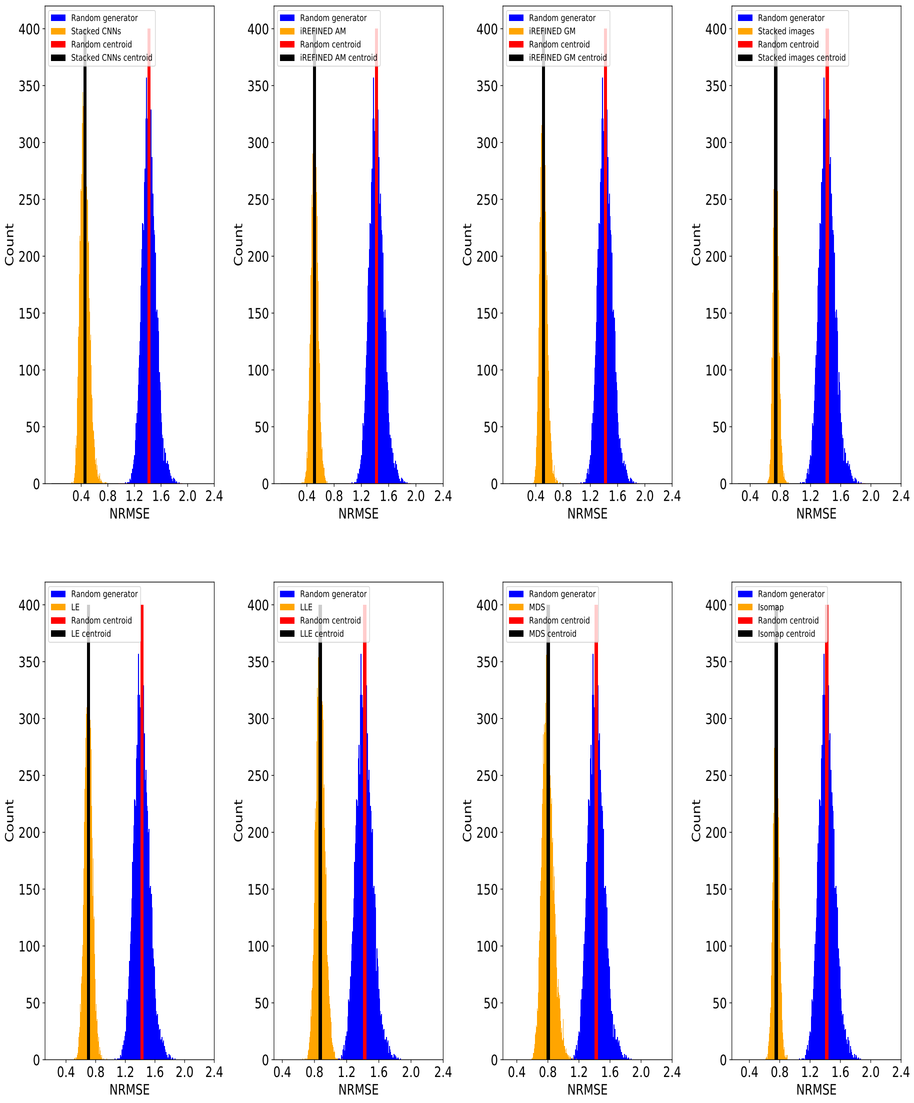

Figure 23: Distribution of NRMSE of all eight models drawn from the Gap statistics test for the 786-0 cell line of the NCI-ALMANAC dataset. The distributions clustered into two groups and their associated cluster centroids are shown with a vertical bar on the histogram plots.

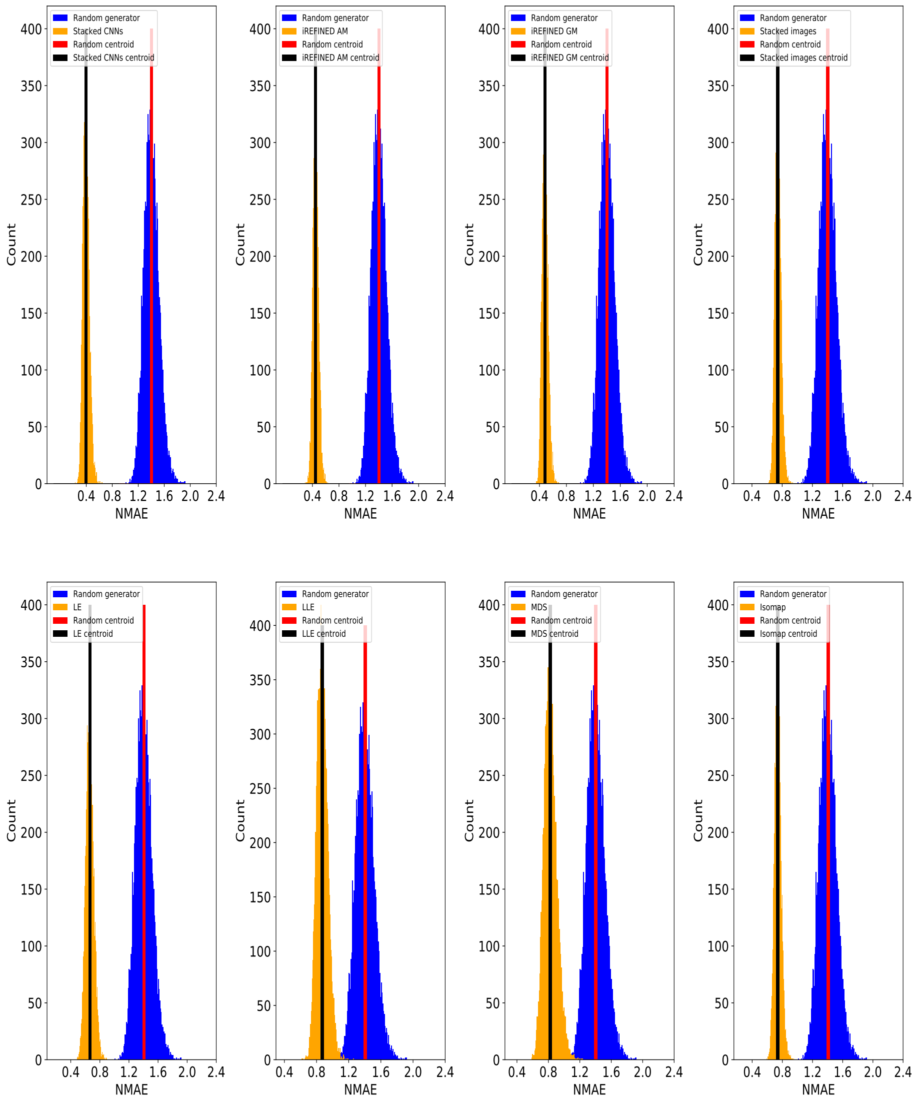

Figure 24: Distribution of NMAE of all eight models drawn from the Gap statistics test for the 786-0 cell line of the NCI-ALMANAC dataset. The distributions clustered into two groups and their associated cluster centroids are shown with a vertical bar on the histogram plots.

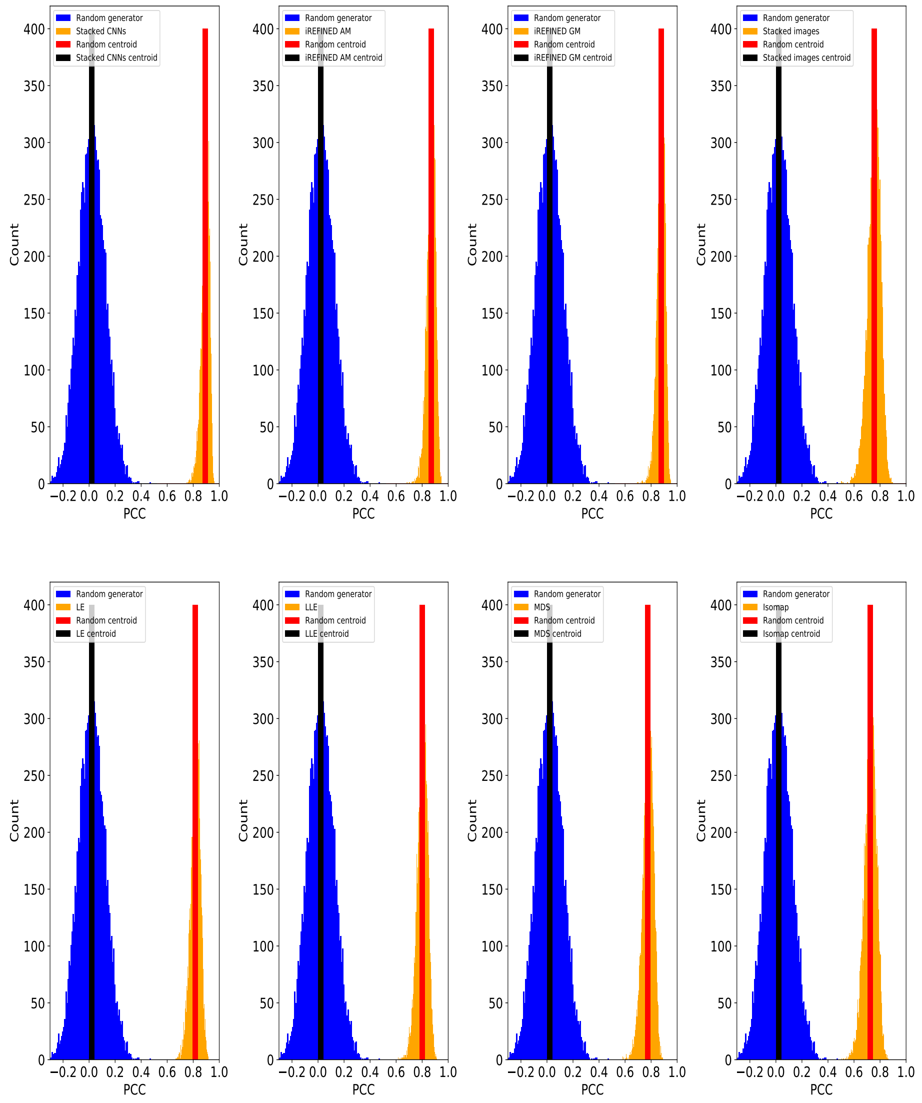

Figure 25: Distribution of PCC of all eight models drawn from the Gap statistics test for the 786-0 cell line of the NCI-ALMANAC dataset. The distributions clustered into two groups and their associated cluster centroids are shown with a vertical bar on the histogram plots.

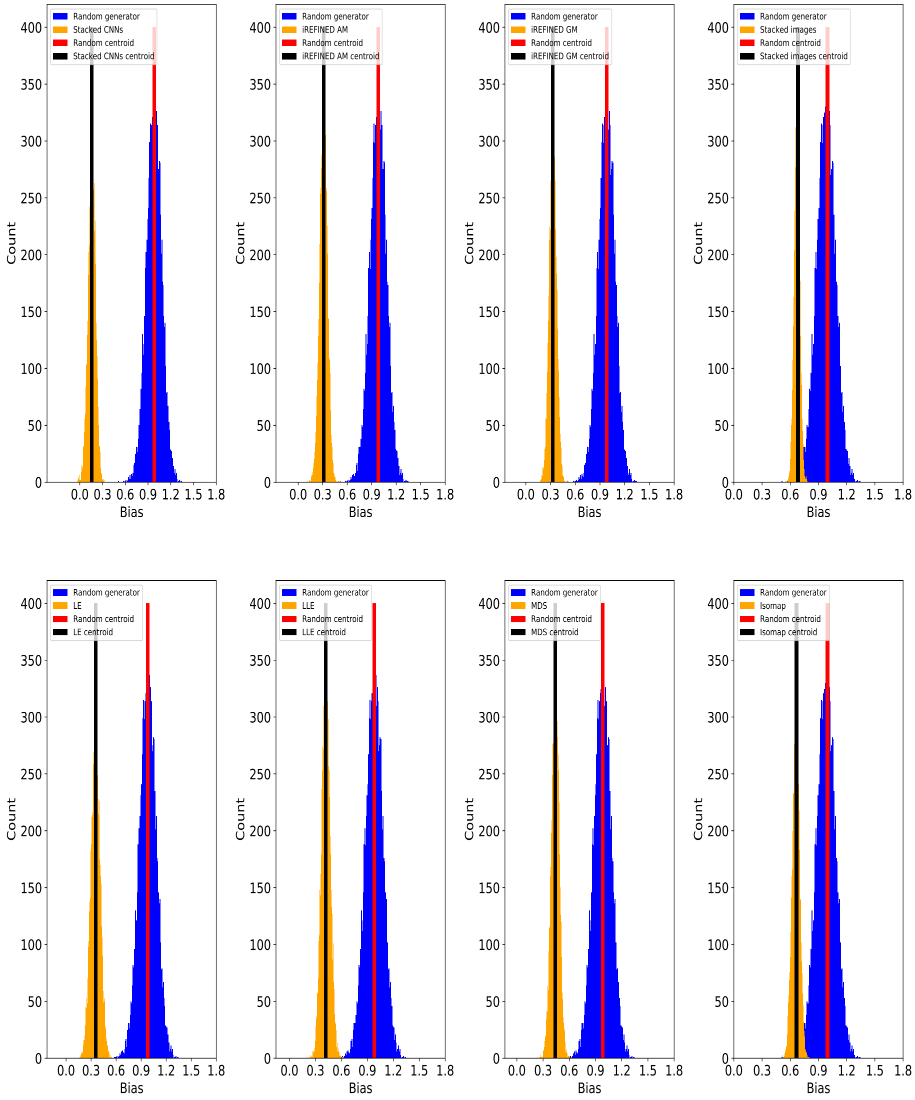

Figure 26: Distribution of Bias of all eight models drawn from the Gap statistics test for the 786-0 cell line of the NCI-ALMANAC dataset. The distributions clustered into two groups and their associated cluster centroids are shown with a vertical bar on the histogram plots.

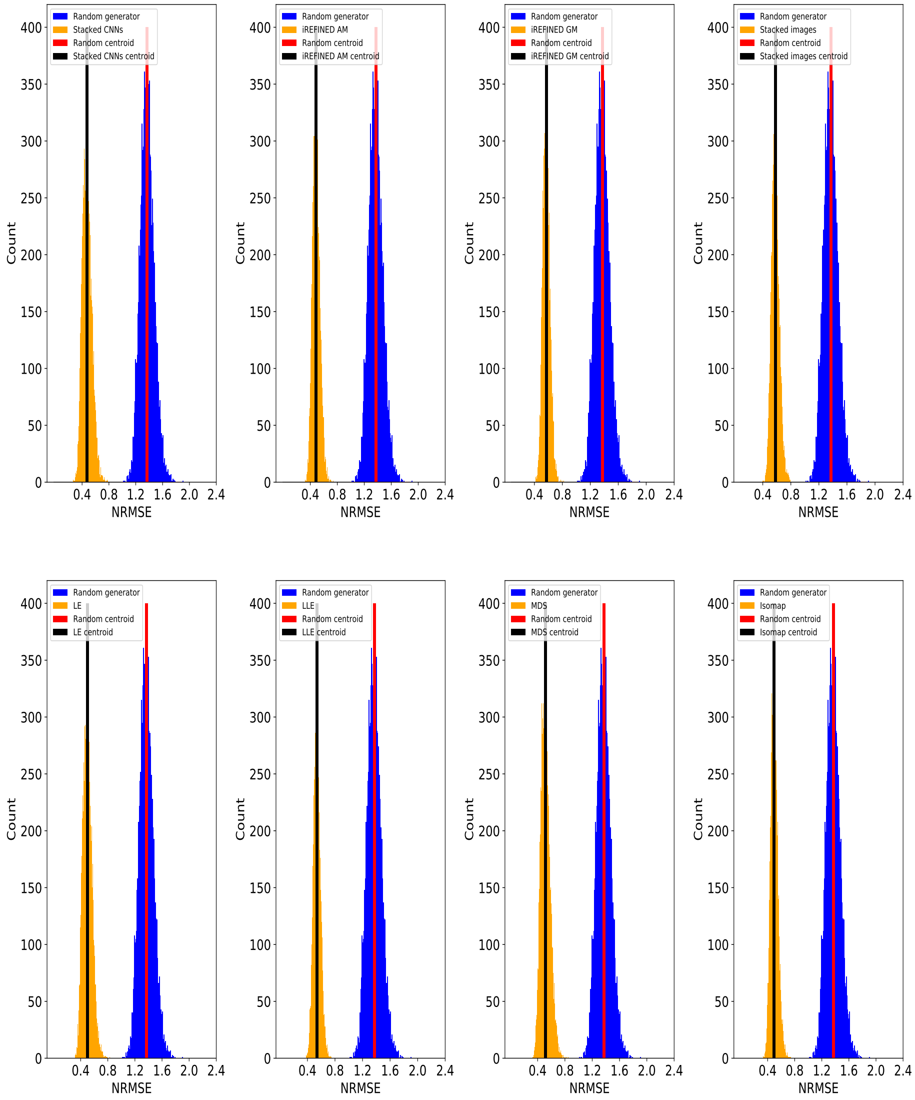

Figure 27: Distribution of NRMSE of all eight models drawn from the Gap statistics test for the HT29 cell line of the NCI-ALMANAC dataset. The distributions clustered into two groups and their associated cluster centroids are shown with a vertical bar on the histogram plots.

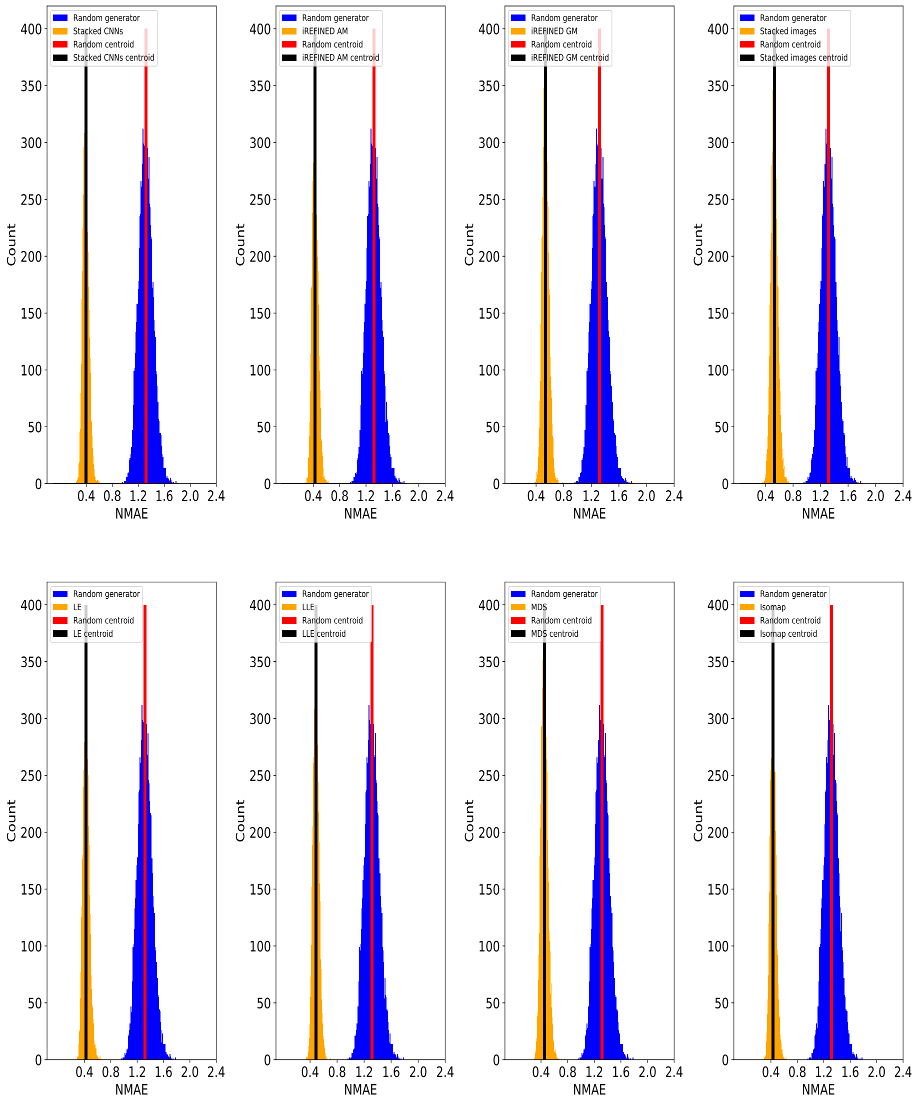

Figure 28: Distribution of NMAE of all eight models drawn from the Gap statistics test for the HT29 cell line of the NCI-ALMANAC dataset. The distributions clustered into two groups and their associated cluster centroids are shown with a vertical bar on the histogram plots.

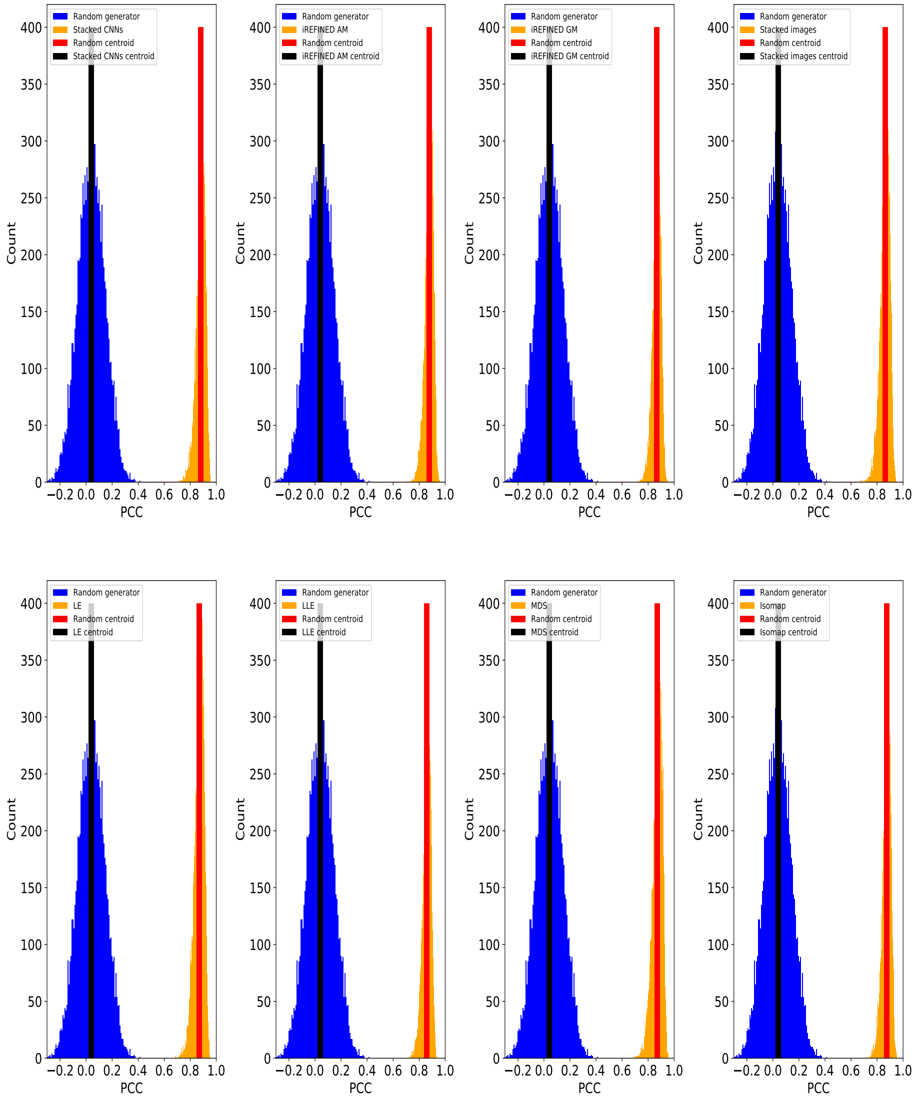

Figure 29: Distribution of PCC of all eight models drawn from the Gap statistics test for the HT29 cell line of the NCI-ALMANAC dataset. The distributions clustered into two groups and their associated cluster centroids are shown with a vertical bar on the histogram plots.

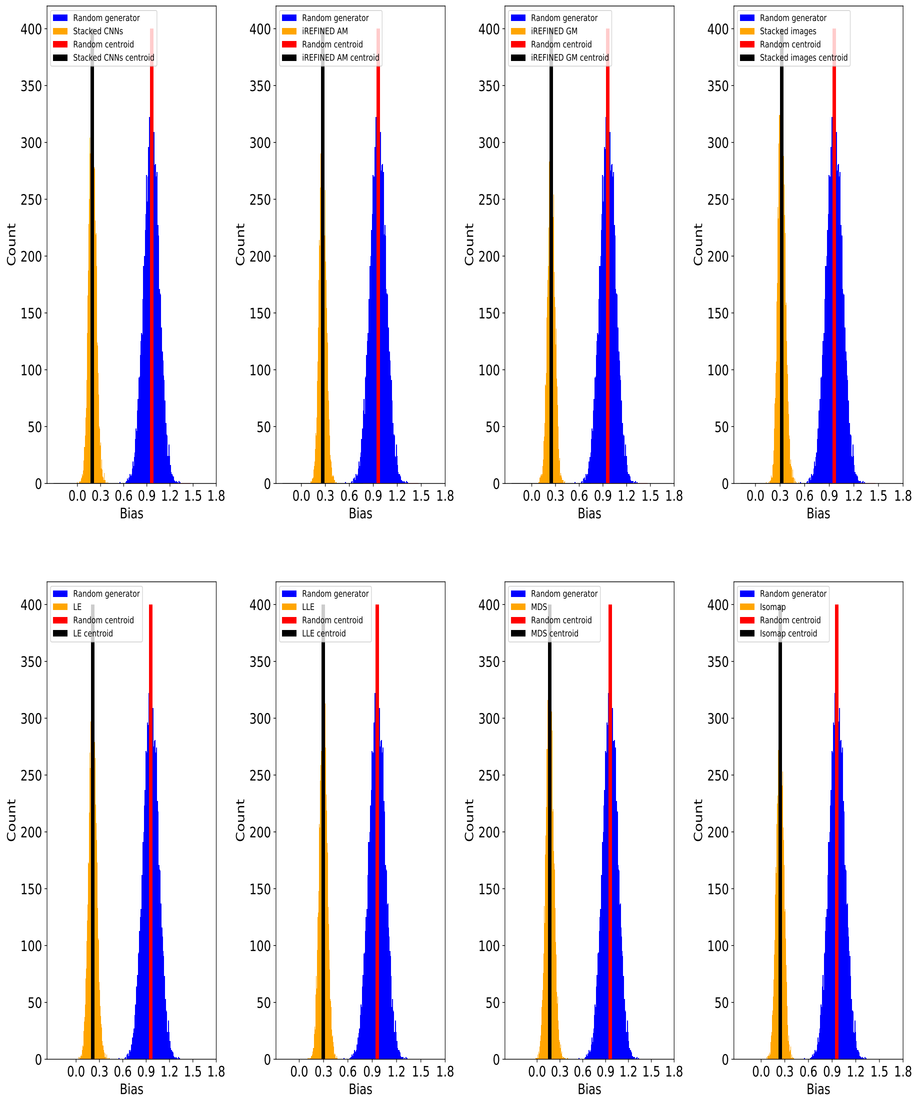

Figure 30: Distribution of Bias of all eight models drawn from the Gap statistics test for the HT29 cell line of the NCI-ALMANAC dataset. The distributions clustered into two groups and their associated cluster centroids are shown with a vertical bar on the histogram plots.

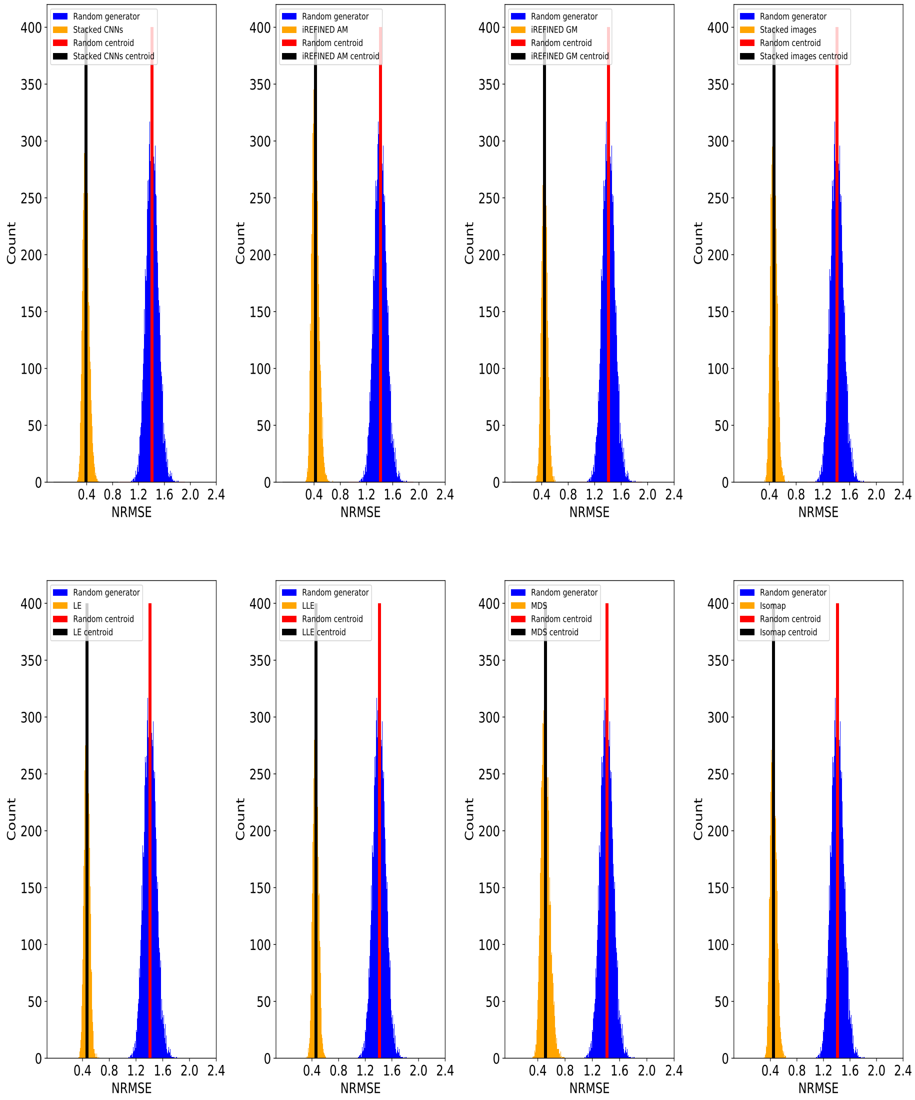

Figure 31: Distribution of NRMSE of all eight models drawn from the Gap statistics test for the HL_60_TB cell line of the NCI-ALMANAC dataset. The distributions clustered into two groups and their associated cluster centroids are shown with a vertical bar on the histogram plots.

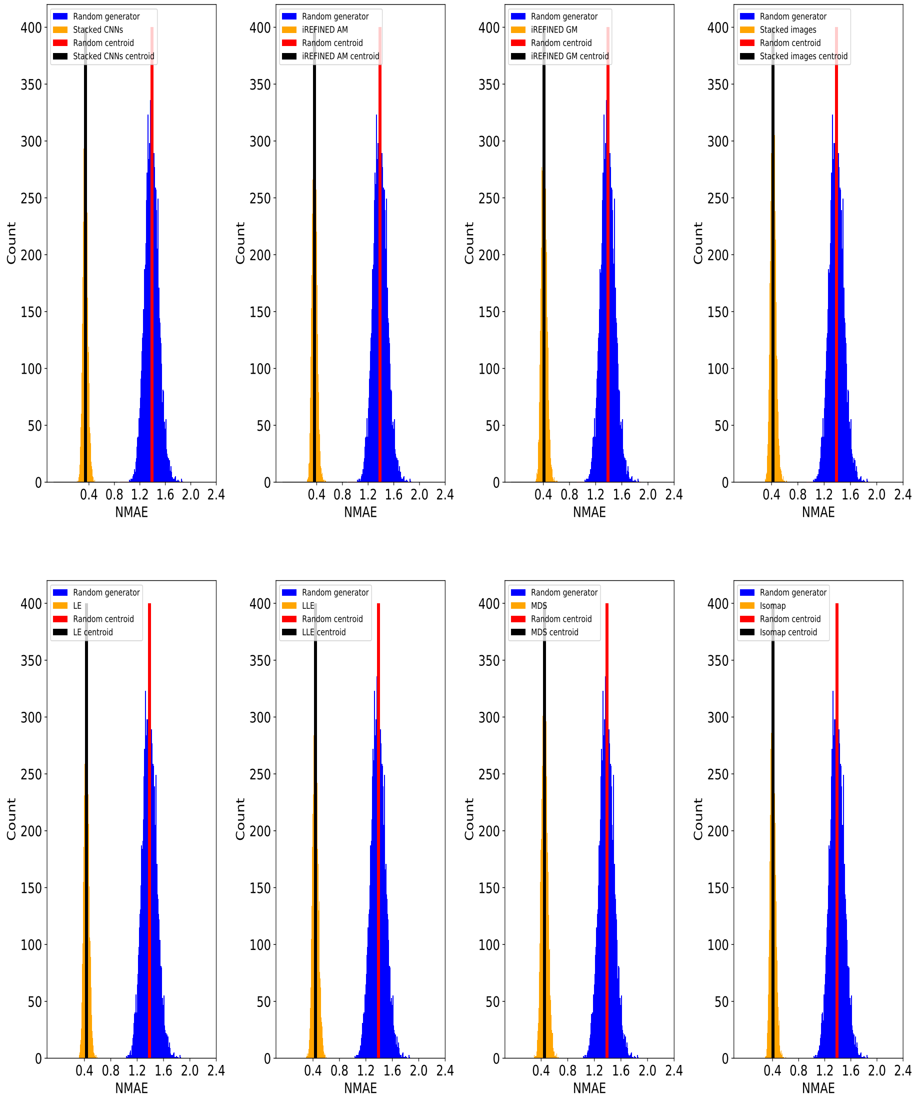

Figure 32: Distribution of NMAE of all eight models drawn from the Gap statistics test for the HL_60_TB cell line of the NCI-ALMANAC dataset. The distributions clustered into two groups and their associated cluster centroids are shown with a vertical bar on the histogram plots.

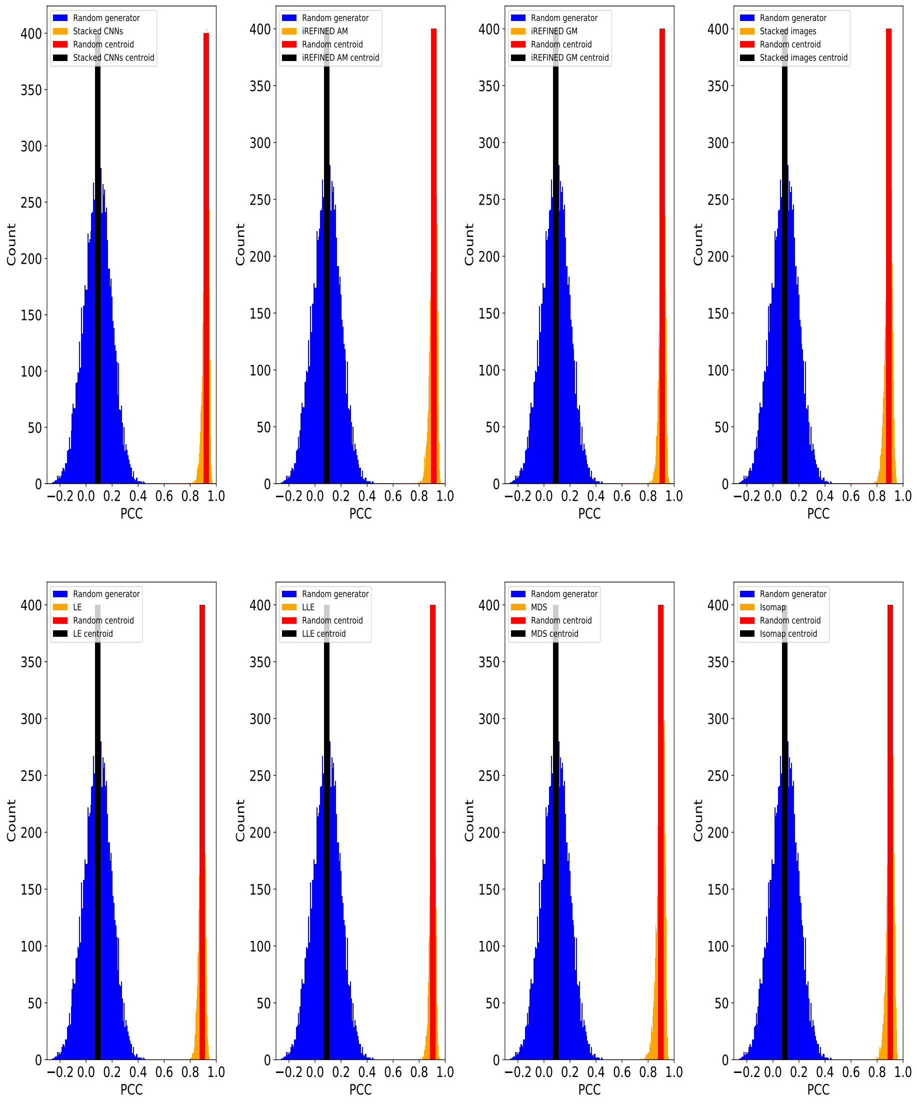

Figure 33: Distribution of PCC of all eight models drawn from the Gap statistics test for the HL_60_TB cell line of the NCI-ALMANAC dataset. The distributions clustered into two groups and their associated cluster centroids are shown with a vertical bar on the histogram plots.

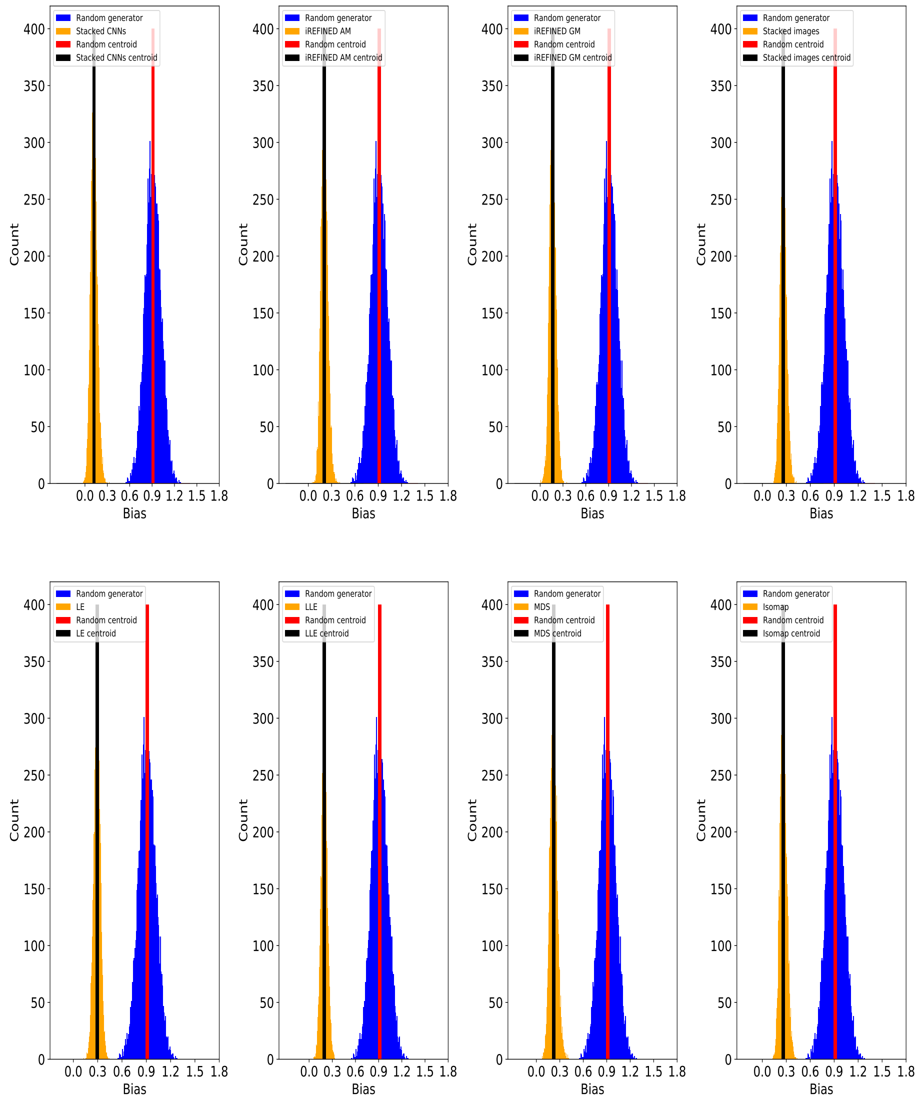

Figure 34: Distribution of Bias of all eight models drawn from the Gap statistics test for the HL_60_TB cell line of the NCI-ALMANAC dataset. The distributions clustered into two groups and their associated cluster centroids are shown with a vertical bar on the histogram plots.
